# Interfacial Energetics of C_2_–C_18_ Aliphatic Moieties on Hydrogenated Si(111) and Si(110) Surfaces: A DFT Study

**DOI:** 10.1021/acs.langmuir.6c00727

**Published:** 2026-05-03

**Authors:** Sara Marchio, Francesco Buonocore, Simone Giusepponi, Massimo Celino

**Affiliations:** 18114 Italian National Agency for New Technologies, Energy and Sustainable Economic Development (ENEA) − C. R. Casaccia, Via Anguillarese 301, Rome 00123, Italy

## Abstract

We present a computational study based on density functional theory to systematically investigate how aliphatic moiety functionalization affects the interfacial electronic structure of H-terminated Si(111) and Si(110) surfaces. We explore the energetics, dipole formation, and charge transfer mechanisms for alkyl, alkenyl, and 1-alkynyl chains containing from 2 to 18 carbon atoms chemisorbed on both crystallographic orientations. Our analysis reveals that alkenyl moieties exhibit pronounced chain-length dependence of surface dipoles and tunneling barriers, whereas alkyl and 1-alkynyl chains show saturation effects for longer chains. We found that H–Si(111) exhibits surface dipoles up to 33% larger than H–Si(110), due to differences in atomic packing density and Si–H bond orientation. The resulting charge injection barriers for both thermionic and tunneling transport are quantified and discussed. The tilted geometry adopted by alkenyl moieties on Si(110) is rationalized through analysis of molecular orbital hybridization with surface states. These results provide quantitative guidelines for engineering interface energetics in silicon-based molecular electronic devices through rational choice of molecular termination and substrate orientation.

## Graphical abstract

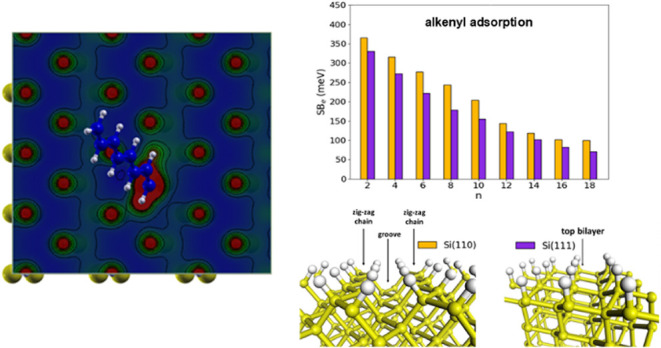

## Introduction

High surface coverage and ordered molecular geometry are crucial for enabling the fabrication of devices based on organically functionalized silicon surfaces, which find many applications including electronic microdevices,^1^ biosensors,^2,3^ and solar cells.^4–6^ This broad range of applicability arises from the distinctive chemical and electronic properties that emerge from the formation of covalent Si–C bonds. The covalent grafting of organic molecules to hydrogenated silicon surfaces can be achieved by several well-established routes. Hydrosilylation of alkenes and alkynes is among the most widely studied approaches. It can be applied to long-chain molecules (>C_8_) under wet-chemical conditions.^7–9^ Hydrosilylation can also be performed in the gas phase, particularly for short-chain (C_3_–C_6_).^10,11^ Another possible method is the two-step halogenation/alkylation approach,^12–16^ which has proven effective in producing highly dense monolayers. There are several examples illustrating the advantages of carbon grafting on silicon surfaces. In photovoltaic devices exposed to air and humidity, passivation with organic monolayers is more effective than hydrogen termination in preventing surface oxidation.^17,18^ In addition, molecular adsorption modifies key interfacial properties such as the work function and the surface dipole,^19,20^ thereby enabling the tuning of charge transport across interfaces, for instance in metal–semiconductor junctions. When addressing the functionalization of silicon, the crystallographic orientation of the surface is an important aspect to consider. Different surface cuts exhibit distinct structural characteristics, including variations in atomic density and in the arrangement of dangling bonds.^21–26^ The influence of the silicon substrate on overlying materials has been clearly demonstrated by Sun et al.,^27^ who showed that graphene grown on Si(111), Si(100), and Si(110) exhibits significant differences in electronic properties, such as work function and carrier density. In the context of functionalization through organic monolayers, the local properties of silicon surfaces arising from their different crystallographic orientations are therefore expected to influence both the structural arrangement of the adsorbed molecules and the electronic transport properties at the interface.

Among the possible crystallographic orientations, we focus here on Si(111) and Si(110) surfaces. Previous studies have shown that these facets are exposed in nanowires grown along the Si(112) direction,^28,29^ providing well-defined sites for covalent functionalization with organic molecules. The high surface-to-volume ratio of nanowires enhances these adhesion sites, making functionalization effects stronger than on bulk surfaces. In the following, we use slab models to describe these facets, as for nanowires larger than ∼10 nm quantum confinement effects are negligible. For smaller nanowires, full nanowire models are required to accurately capture confinement effects.^30,31^ The Si(111) surface is the most extensively studied: it consists of a bilayer structure with dangling bonds that can be passivated by hydrogen, forming bonds perpendicular to the surface.^25^ On Si(110),^23,24^ hydrogen passivation produces tilted bonds aligned with the underlying Si–Si bonds. This surface has attracted attention due to its anisotropy and the formation of one-dimensional structures at the interface. Since the first report of densely packed alkyl monolayers,^7^ functionalization of Si(111) has been widely investigated.^32–35^ The functionalization with alkenes and alkynes of hydrogenated Si(111) forms alkyl and alkenyl monolayers, respectively.^36^ Methyl-terminated Si(110) surfaces have been studied by Gupta et al.^37^ who demonstrated long-term stability against oxidation. Additionally, Zhang et al.^38^ investigated Si(112), Si(111), and Si(110) crystallographic orientations of silicon functionalized with alkyl monolayers in the context of electrode fabrication.

Here, we extend the previous density functional theory (DFT) study of Si(111) functionalized with C_2_–C_10_ aliphatic chains to longer chains (C_12_–C_18_) and to the Si(110) surface. The adhesion geometries used in this work correspond to the minimum-energy configurations identified through the screening performed in our previous study.^39^ In the present work, we analyze how chain length and carbon–carbon bond type (single, double, or triple) affect the structure of the adsorbed molecules on both surfaces. Moreover these configurations are characterized computationally to clarify how the interfacial electronic structure of H-terminated Si(111) and Si(110) surfaces are affected by aliphatic moiety functionalization, with particular attention to the interface dipole and charge transfer, and how these quantities depend on the local properties of each surface orientation. Finally, the charge injection barriers for both thermionic and tunneling transport are calculated and discussed.

## Theoretical Methods

We performed ab initio calculations using the Plane-Wave Self-Consistent Field (*PWscf)* code implemented in the Quantum ESPRESSO package.^40–42^ Vanderbilt pseudopotentials were adopted for the electron–ion interactions,^43^ and the vdW-DF-cx functional was chosen. The choice of the vdW-DF-cx functional is motivated by its proven ability to accurately account for van der Waals interactions, as well as its previous application in the study of metal–organic interfaces.^44–47^ Dipole corrections were applied following the protocol described by Bengtsson.^48^ The kinetic energy cutoff was set to 40 Ry for wave functions and 400 Ry for charge density. A 2 × 2 × 1 Monkhorst–Pack^49^ grid was used for the self-consistent calculations, with a convergence threshold of 1 × 10^–9^ Ry. The electronic structure, in terms of density of states (DOS), was computed using an 8 × 8 × 1 k-point grid.

The H–Si(110) and H–Si(111) surfaces differ significantly in symmetry and local atomic arrangements, as shown in Figure 1. The H–Si(110) surface has a rectangular lattice with p2mg symmetry. The first Si layer forms quasi-one-dimensional zigzag chains, while the second layer forms zigzag grooves, as described by Matsushita et al.^23^ Hydrogen passivation on Si(110) produces tilted Si–H bonds that follow the orientation of the underlying Si–Si bonds. In contrast, the H–Si(111) surface has a bilayer structure with Si–H bonds perpendicular to the surface and a hexagonal lattice with p3m1 symmetry.^50^ The H atoms are more closely spaced on Si(110) than on Si(111), while the surface Si atom density is higher on Si(111).^27^

**Figure 1:**
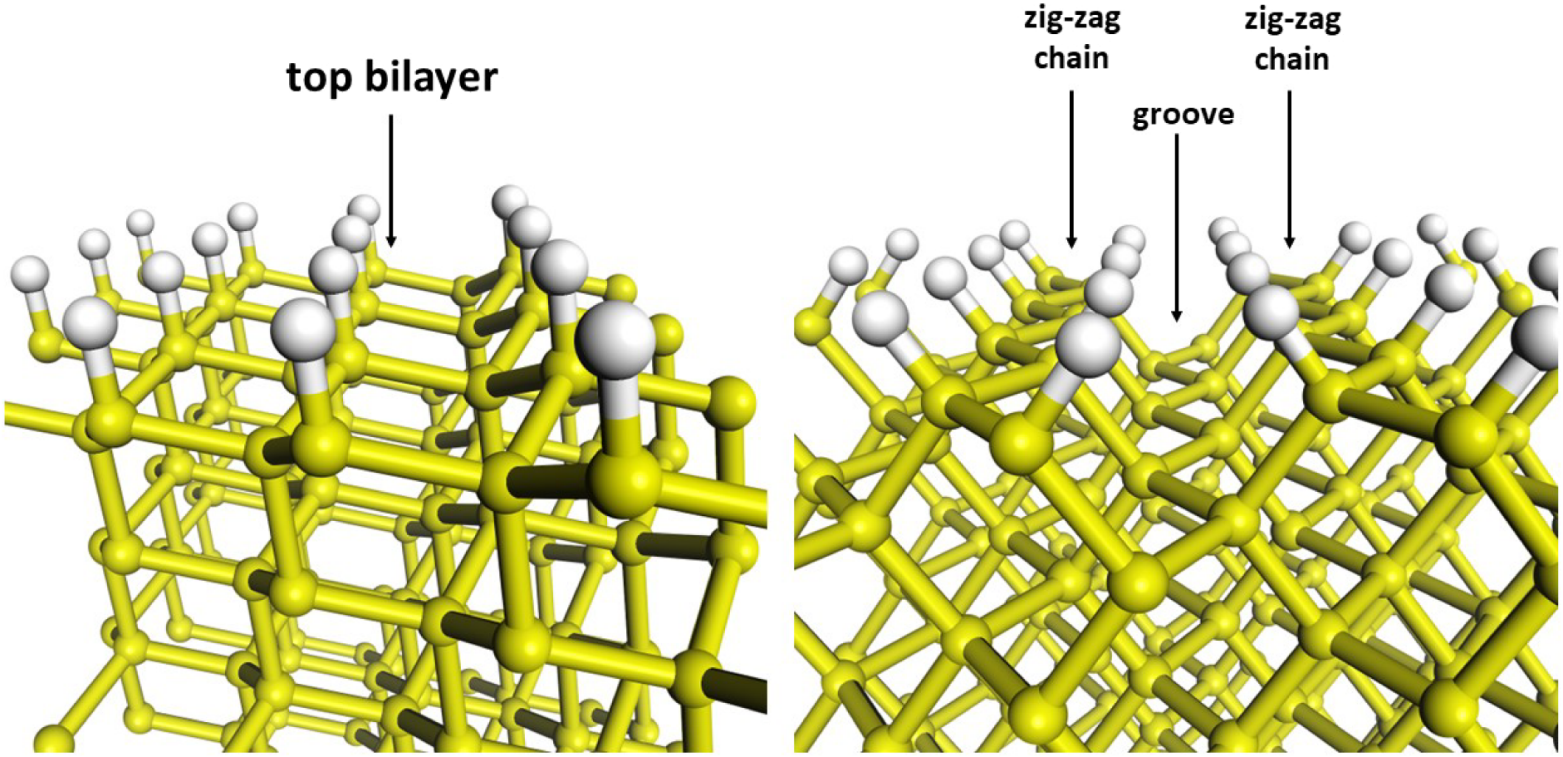
Views of the hydrogenated Si(111) (left panel) and Si(110) (right panel) surfaces.

The H–Si(110) and H–Si(111) surfaces were modeled using slab geometries in which the dangling bonds of the top layer are passivated by hydrogen. Representative geometries are shown in Figure 2. The H–Si(110) slab (Figure 2a) is 13.5 Å thick and is made of eight atomic layers. The periodic supercell contains 192 Si atoms and 24 hydrogen atoms, with lateral dimensions of L_*x*_ = 16.40 Å and L_*y*_ = 15.47 Å. The H–Si(111) (Figure 2b) slab is 15 Å thick and consists of five bilayers. Its supercell includes 160 Si atoms and 16 hydrogen atoms, with lateral dimensions of L_*x*_ = 15.47 Å and L_*y*_ = 13.39 Å. The supercell area in the *xy*-plane is *A*_*Si*(110)_ = 254 Å^2^ and *A*_*Si*(111)_ = 207 Å^2^, corresponding to coverages of 0.39 and 0.48 moieties/nm^2^, respectively. On each surface, we studied the adsorption of C_2_–C_18_ aliphatic moieties, namely alkyl derived from *alkane* (C_n_H_2n+2_) chains with only single C–C bonds, alkenyl derived from *alkene* (C_n_H_n+2_) chains with alternating single and double carbon–carbon bonds, and 1-alkynyl derived from *alkyne* (C_n_H_2n–2_) chains containing a carbon–carbon triple bond attached to an alkyl radical. In both Si(110) and Si(111) systems the length of the supercell along the *z* direction (L_*z*_) is approximately 50 Å for chemisorbed C_2_–C_10_ chains and 65 Å for C_12_–C_18_ chains, ensuring a sufficient vacuum space to prevent interaction among supercell replicas.

**Figure 2:**
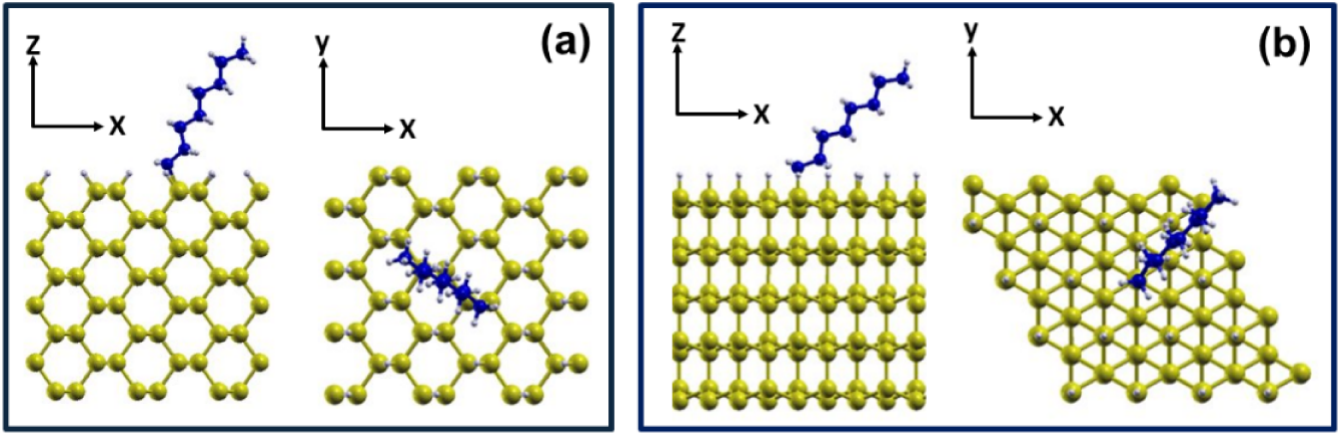
Side (left) and top (right) views of (a) the Si(110) surface and (b) the Si(111) surface with the adsorbed C_8_ alkyl moiety.

The grafting of the molecular moieties is achieved by removing one hydrogen atom from the passivation layer and replacing it with the C_1_ atom of the moiety, thereby forming a bond with the exposed Si atom, as shown in Figure 3. Multiple adhesion configurations were explored in order to select the ones with minimum energy.^39^ Specific examples of adsorption geometries for alkyl, alkenyl and 1-alkynyl moieties on hydrogenated Si(110) and Si(111) surfaces are shown in Figure S1 of the Supporting Information.

**Figure 3:**
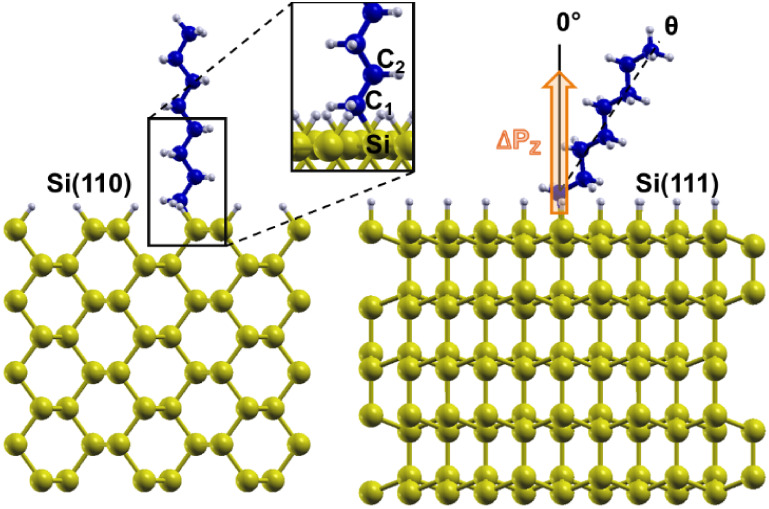
Graphical sketch of the structural parameters analyzed. Left panel: Si, C_1_, and C_2_ atoms. Right panel: tilting angle θ and schematic representation of the z-oriented dipole.

All geometries were relaxed using the Broyden–Fletcher–Goldfarb–Shanno (BFGS) algorithm. We allowed a convergence of the total energy below 0.0001 Ry. The systems were relaxed with a convergence threshold of 0.001 Ry/Bohr on the interatomic forces. In the five bilayers of Si(111) (the eight layers of the Si(110)) slab, the H atoms and the moiety were only adsorbed over the top layer, and the two bottom bilayers (three bottom layers) were fixed in the bulk positions.

The stability of the system was evaluated through the bond dissociation energy *E*_*D*_ of the moiety from the surface, defined as

1 $$E_{D}=E_{moiety}+E_{H^{*}-Si}-E_{tot}$$

where *E*_*moiety*_ is the energy of the isolated moiety, $E_{H^{*}-Si}$ is the energy of the silicon slab with a hydrogen vacancy, and *E*_*tot*_ is the total energy of the system with the moiety chemisorbed on the slab.

The system dipole along the *z* direction can be calculated as

2 $$P_{z}^{tot}=\int_{0}^{L_{z}}\rho_{xy}\left(z\right)\times zdz$$

where *L*_*z*_ is the supercell dimension perpendicular to the surface, and *ρ*_*xy*_(*z*) is the charge density integrated over the *xy* plane at each *z*. The contribution of molecular adsorption to the dipole, Δ*P*_*z*_, can be isolated as the difference between the total dipole of the system and that of the fully passivated slab $P_{z}^{\left(H-Si\right)}$ :

3 $$\Delta P_{z}=P_{z}^{tot}-P_{z}^{\left(H-Si\right)}$$

The corresponding electron energy shift is defined as

4 Ψ=eΔV

where Δ*V* = 4πΔ*P*_*z*_/*A* is the electrostatic potential step and *A* is the area of the supercell in the *xy*-plane. This energy allows the estimation of the effect of the moiety adsorption on charge transfer in a silicon/organic molecule/metal heterojunction. Indeed, the surface dipoles modulate the Schottky barrier height for electrons (*SB*_*e*_) and holes (*SB*_*h*_) according to the following expressions:^51,52^

5.a $$SB_{e}=\Phi -\chi -\Psi$$

5.b $$SB_{h}=E_{g}-\Phi +\chi +\Psi$$

where Φ is the metal work function of the metal, *χ* is the electronic affinity of the semiconductor, and *E*_*g*_ is the silicon band gap. The sign of Ψ is positive when the molecular dipole points outward from the surface to which it is adsorbed, with its positive end toward the moiety and negative end toward the semiconductor. In this configuration, the Schottky barrier for electrons is reduced, lowering the energy step at the interface. The opposite effect occurs when the dipole points toward the semiconductor. Equations 5 and 6 provide a quantitative evaluation of the charge injection barriers at the interface.

We also analyzed the energetics at the H–Si(110)/molecule and H–Si(111)/molecule interfaces to estimate the tunneling barriers for electrons and holes. In Figures S2–S55 the projected density of states (PDOS) are reported. For each molecule, we determined the HOMO and LUMO levels from the main peaks of the molecular PDOS near the Fermi level (as in Figure S56). These levels were then compared with the silicon band edges. To make this comparison consistent, the energy scales were aligned by superimposing the plane-averaged electrostatic potential of bulk silicon with that of the central layers of the slab. We estimated the vacuum level of each adsorption configuration as the constant plane-averaged electrostatic energy in the vacuum gap far away from the top atomic layer, as shown in Figure S57.

The tunneling barriers were then calculated as

6.a $$\Delta LUMO=LUMO-E_{c}$$

6.b $$\Delta HOMO=E_{v}-HOMO$$

where *E*_*c*_ and *E*_*v*_ are the conduction- and valence-band edge energies, respectively.

## Results and Discussion

### Structural Properties

The adhesion geometries of the C_2_–C_18_ molecules on the Si(111) and Si(110) slabs were selected as the most stable configurations, i.e., those with the highest bond dissociation energy, after exploring several possible adsorption geometries. All screened structures are available in a data set published on the Materials Cloud platform^53^ and described in our previous work.^39^ Tables 1–3 report the bond dissociation energies (as defined by eq 1 in the Methods section) for all selected configurations, along with the tilting angle, the Si–C bond length, and the length of the C–C bond closest to the surface. These structural parameters are sketched in Figure 3. Among the investigated groups, the 1-alkynyl moieties exhibit the highest bond dissociation energies (*E*_*D*_ ≈ 6 eV), followed by the alkenyl group (*E*_*D*_ ≈ 4.5 eV) and the alkyl group (*E*_*D*_ ≈ 4.0 eV). For alkyl chains, the tilting angle θ decreases as the number of carbon atoms increases from C_2_ to C_8_. On the Si(110) surface, the most stable configurations of alkyl chains longer than C_4_ correspond to molecules that are nearly perpendicular to the surface, whereas on Si(111) the angle θ stabilizes at values >30°. The molecular tilt is a key parameter since it directly affects the surface dipole, as well as the stability and quality of the resulting organic monolayer. For example, previous studies have shown that the tilt angle decreases as the molecular coverage on the surface increases.^54^

**Table 1:** Bond Dissociation Energy and Structural Parameters of Alkyl Chains Grafted on Si(111) and Si(110) Surfaces

	*E*_*D*_ (eV)	θ (°)	d(C_1_–C_2_) (Å)	d(Si–C_1_) (Å)
H–Si(111)–C_2_	3.923	57.792	1.529	1.909
H–Si(111)–C_4_	4.036	42.340	1.528	1.907
H–Si(111)–C_6_	4.031	37.941	1.528	1.904
H–Si(111)–C_8_	4.051	36.406	1.529	1.905
H–Si(111)–C_10_	4.046	36.320	1.526	1.904
H–Si(111)–C_12_	4.062	34.987	1.527	1.902
H–Si(111)–C_14_	4.053	48.136	1.532	1.910
H–Si(111)–C_16_	4.055	47.906	1.532	1.910
H–Si(111)–C_18_	4.062	32.084	1.527	1.902
H–Si(110)–C_2_	3.878	38.051	1.532	1.910
H–Si(110)–C_4_	3.968	21.796	1.531	1.907
H–Si(110)–C_6_	3.990	5.515	1.530	1.898
H–Si(110)–C_8_	4.002	4.185	1.530	1.897
H–Si(110)–C_10_	4.000	4.889	1.528	1.894
H–Si(110)–C_12_	3.974	4.364	1.530	1.905
H–Si(110)–C_14_	3.975	4.065	1.531	1.905
H–Si(110)–C_16_	3.983	3.674	1.530	1.905
H–Si(110)–C_18_	3.983	2.870	1.530	1.904

**2:** Bond Dissociation Energy and Structural Parameters of Alkenyl Chains Grafted on Si(111) and Si(110) Surfaces

	*E*_*D*_ (eV)	θ (°)	d(C_1_–C_2_) (Å)	d(Si–C_1_) (Å)
H–Si(111)–C_2_	4.446	50.370	1.339	1.872
H–Si(111)–C_4_	4.663	40.865	1.352	1.864
H–Si(111)–C_6_	4.694	41.153	1.357	1.864
H–Si(111)–C_8_	4.699	34.952	1.358	1.858
H–Si(111)–C_10_	4.709	34.764	1.360	1.860
H–Si(111)–C_12_	4.738	33.447	1.361	1.859
H–Si(111)–C_14_	4.720	32.960	1.361	1.861
H–Si(111)–C_16_	4.747	32.493	1.361	1.861
H–Si(111)–C_18_	4.748	32.189	1.361	1.861
H–Si(110)–C_2_	4.386	39.303	1.340	1.874
H–Si(110)–C_4_	4.621	31.122	1.353	1.864
H–Si(110)–C_6_	4.676	36.933	1.356	1.858
H–Si(110)–C_8_	4.696	34.722	1.359	1.857
H–Si(110)–C_10_	4.694	35.916	1.360	1.860
H–Si(110)–C_12_	4.699	34.672	1.360	1.860
H–Si(110)–C_14_	4.704	33.736	1.360	1.859
H–Si(110)–C_16_	4.704	33.005	1.360	1.858
H–Si(110)–C_18_	4.685	32.536	1.361	1.859

**Table 3:** Bond Dissociation Energy and Structural Parameters of 1-Alkynyl Chains Grafted on Si(111) and Si(110) Surfaces

	*E*_*D*_ (eV)	θ (°)	d(C_1_–C_2_) (Å)	d(Si–C_1_) (Å)
H–Si(111)–C_2_	5.873	1.983	1.219	1.824
H–Si(111)–C_4_	5.782	35.479	1.223	1.812
H–Si(111)–C_6_	5.801	37.741	1.223	1.811
H–Si(111)–C_8_	5.827	58.784	1.224	1.813
H–Si(111)–C_10_	5.913	67.188	1.225	1.816
H–Si(111)–C_12_	5.943	65.597	1.225	1.820
H–Si(111)–C_14_	5.935	65.086	1.226	1.820
H–Si(111)–C_16_	5.976	65.552	1.226	1.820
H–Si(111)–C_18_	6.018	65.548	1.225	1.820
H–Si(110)–C_2_	5.822	26.090	1.219	1.824
H–Si(110)–C_4_	5.777	36.607	1.223	1.813
H–Si(110)–C_6_	5.838	44.352	1.223	1.812
H–Si(110)–C_8_	5.862	48.728	1.223	1.812
H–Si(110)–C_10_	5.887	52.491	1.223	1.811
H–Si(110)–C_12_	6.027	66.023	1.225	1.819
H–Si(110)–C_14_	5.855	65.523	1.226	1.824
H–Si(110)–C_16_	5.913	66.151	1.227	1.823
H–Si(110)–C_18_	6.089	68.275	1.224	1.813

For the alkenyl group, the decrease in θ with increasing carbon chain length from C_2_ to C_8_ is less pronounced than for the alkyl group. For chains longer than C_8_, the most stable configurations on both the Si(110) and Si(111) surfaces show a tilt angle of approximately θ ≈ 35°. The 1-alkynyl group exhibits a distinct trend in molecular tilt. For chains shorter than C_10_, the tilt increases with chain length. For chains longer than C_10_ both the Si(110) and Si(111) surfaces exhibit stable configuration with θ ≈ 65°. Experimental tilt angle data are available for alkyl and alkenyl C_12_–C_18_ monolayers grafted on H–Si(111) surfaces, as reported by L. Scheres et al.^55^ The values obtained in this work differ by less than 10° from the experimental ones, indicating good agreement with the observed adsorption geometries.

As shown in Figure S1, the alkyl and 1-alkynyl moieties adopt a characteristic orientation in which the first two hydrogen atoms point upward, the next two downward, and so on, so that the molecular bisector plane is approximately perpendicular to the slab. For the alkenyl group, the molecular plane on the Si(111) surface is perpendicular to the surface, while on the Si(110) surface it is inclined. To understand why this peculiar tilt is energetically favored, we analyzed the molecular orbitals of the alkenyl moiety on Si(110). The results are discussed in the charge density analysis section.

For alkyl and 1-alkynyl moieties, no clear trends are observed in the C–C bond lengths near the surface. The C_1_–C_2_ distance is ≈ 1.53 Å for alkyl chains, consistent with a single C–C bond, and ≈1.22 Å for 1-alkynyl chains, which contain a triple C–C bond. The Si–C_1_ distance is ≈1.91 Å for alkyl chains and ≈1.81 Å for 1-alkynyl chains. The alkenyl group shows a distinct behavior: the C_1_–C_2_ bond length increases for chains shorter than C_10_ and converges to ≈1.36 Å for chains longer than C_10_. Similarly, the Si–C_1_ distance decreases for chains shorter than C_10_ and stabilizes at around 1.86 Å for chains longer than C_10_ on both surfaces.

### Surface Dipoles and Schottky Barrier

We compare the electronic response of the Si(111) and Si(110) surfaces to the removal of one hydrogen atom from the passivation layer and to the subsequent adsorption of each moiety. This process induces a charge redistribution in the material, resulting in a positive surface dipole. Indeed, we demonstrated that when the Si–C bond is formed negative charges move from the surface to the carbon, while the charge localized on the moiety’s terminal group furthest from the surface is positive, resulting in a dipole pointing outward.^56^ This mechanism is quantified by computing the dipole, Δ*P*_*z*_, as described in eq 3 of the Methods section. The values depend only weakly on surface coverage, as demonstrated by the data reported in Table S1. This quantity therefore allows us to identify the effects arising from the orientation of the silicon surface. The results are shown in Figure 4. Extending our previous analysis of C_2_–C_10_ chains adsorbed on Si(111)^56^ to longer chains (C_12_–C_18_) and to the Si(110) surface, we find that the overall trends are preserved and confirmed for both surface orientations. In the following the surface dipole is expressed in Debye (D). For alkyl moieties, the surface dipole stabilizes for chains longer than C_4_, reaching values in the range of 0.93–0.97 D (energy step of 160–180 meV at the given Si(111) surface coverage) on Si(111) and 0.69–0.76 D (energy step of 100–112 meV at the given Si(110) surface coverage) on Si(110). A similar behavior is observed for 1-alkynyl moieties, with dipole values stabilizing at 1.5–1.75 D (270–320 meV) on Si(111) and 1.35–1.52 D (190–230 meV) on Si(110). In contrast, alkenyl chains show a monotonic increase in dipole with chain length, from 0.67 to 2.26 D (120 to 411 meV) on Si(111) and from 0.46 to 2.08 D (68 to 309 meV) on Si(110). In all cases, the dipole on Si(110) is systematically lower than on Si(111), highlighting a clear surface orientation-dependent response. The origin of this different surface response is further analyzed in the section dedicated to the charge density analysis.

**Figure 4:**
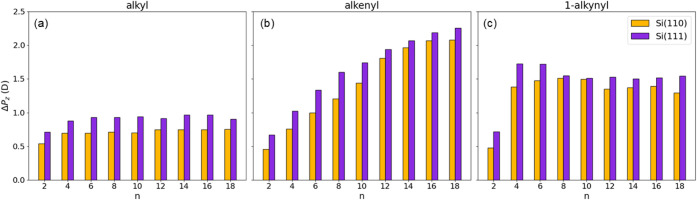
Dipole variation for (a) alkyl, (b) alkenyl, and (c) 1-alkynyl moieties adsorbed on Si(111) (violet bars) and Si(110) (yellow bars) as a function of the number of carbon atoms in the chains.

The observed variations in the surface dipole directly affect the energetics of the interface and can be used to estimate the Schottky barrier heights for electrons and holes, as described in the Methods section (eqs 5 and 6). Due to the constraints imposed by periodic boundary conditions, it is not possible to achieve identical surface coverage on the two crystal facets. To enable a meaningful comparison at equivalent coverage, the potential ψ used in the Schottky barrier evaluation was rescaled. Specifically, a rescaled energy step was defined as $\psi_{res}=\psi A_{Si\left(110\right)/Si\left(111\right)}/A_{eff}$ , where *A*_*eff*_ is an intermediate value between the supercell areas of the two surfaces. We consider a Si–H/molecule/metal heterojunction with Hg as the metal, assuming Φ_Hg_ = 4.5 eV, χ_Si_ = 4.06 eV^57^ and E_g_ = 1.12 eV. The results are shown in Figure 5. For alkyl chains on Si(110), the barrier heights stabilize at approximately 320 meV for electrons and 800 meV for holes. On Si(111), the barriers follow a similar trend but are systematically ∼30 meV lower (higher) for electrons (holes). For alkenyl chains on Si(110), the electron barrier decreases monotonically from 365 to 99 meV, while the hole barrier increases from 755 to 1020 meV. On Si(111), the barriers exhibit similar behavior but are consistently 15–65 meV lower (higher) for electrons (holes). For 1-alkynyl chains, the electron (hole) barrier decreases (increases) rapidly up to C_6_ and then stabilizes. On Si(110), the stabilized values are ∼215 meV for electrons and ∼900 meV for holes, whereas on Si(111) they are, respectively, ∼190 meV and ∼930 meV. As observed for the surface dipoles, the Schottky barrier heights confirm the trends previously reported in Buonocore et al.,^56^ extending their validity to longer chains (C_12_–C_18_). Moreover, the analysis quantifies the dependence of the barrier heights on the surface orientation, revealing systematically higher electron barriers and lower hole barriers on Si(110) compared to Si(111). This behavior highlights the interplay between surface orientation and the electronic structure of the molecular end group, which is further analyzed in the charge density section of the results.

**Figure 5:**
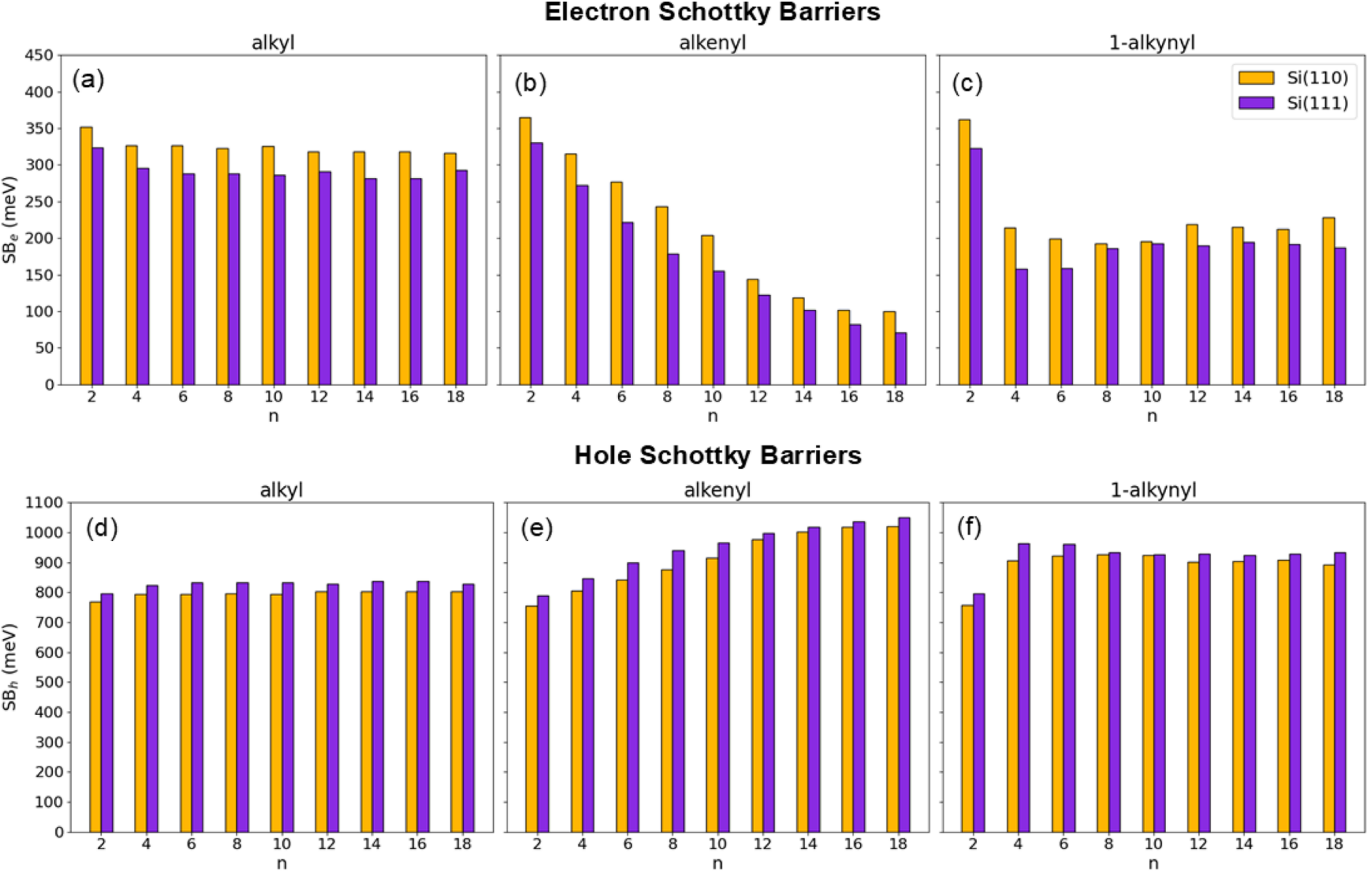
Electron (a–c) and hole (d–f) Schottky barriers for (a, d) alkyl, (b, e) alkenyl, and (c, f) 1-alkynyl moieties adsorbed on Si(111) (violet bars) and Si(110) (yellow bars) as a function of the number of carbon atoms in the chains.

### Tunneling Barriers

We characterized the H–Si(111)–aliphatic chain and H–Si(110)–aliphatic chain interfaces by analyzing the charge transport in terms of the tunneling barriers for electrons and holes. The electron tunneling barrier ΔLUMO is evaluated as the energy difference between the molecular LUMO level and the silicon conduction band edge (eq 7). Similarly, the hole tunneling barrier ΔHOMO is the difference between the valence band edge and the molecular HOMO level (eq 8). HOMO and LUMO levels are determined by the PDOS analysis (see Figure S56). This approach provides direct insight into how molecular adsorption and surface orientation influence charge injection across the organic/inorganic heterointerface. The results are reported in Figure 6 for alkyl, alkenyl, and 1-alkynyl groups.

**Figure 6:**
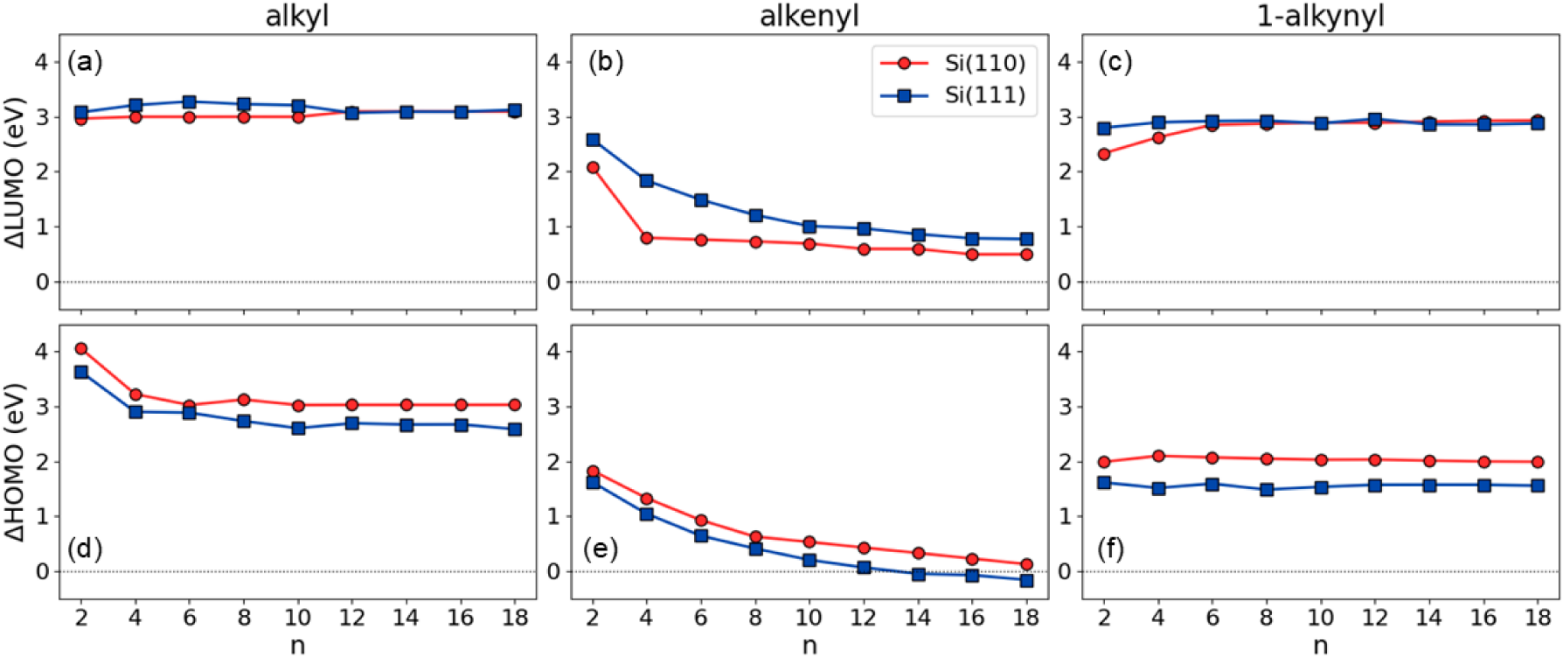
ΔLUMO (upper plots) and ΔHOMO (lower plots) tunneling barriers for alkyl (a, d), alkenyl (b, e), and 1-alkynyl (c, f) aliphatic chains adsorbed on Si(110) (red circles) and Si(111) (blue squares).

For alkyl moieties, ΔHOMO decreases by ∼1 eV for alkyl chains from C_2_ to C_4_. ΔLUMO increases by roughly 0.5 eV for 1-alkynyl chains from C_2_ to C_8_ only for adsorption on the Si(110) surface. The tunneling barriers remain nearly constant for chains longer than C_8_. Additionally, a systematic upward shift of slightly less than 0.5 eV is observed in the ΔHOMO of alkyl and 1-alkynyl chains adsorbed on Si(110) compared to the same chains on Si(111).

In the case of alkenyl adsorption, the tunneling barriers show a clear dependence on the chain length, which tends to saturate as the chain length increases. The ΔHOMO (ΔLUMO) barrier decreases by ∼1.6–1.8 eV as the chain length increases from C_2_ to C_18_, ranging from 1.62 to −0.16 (2.60 to 0.77) eV for Si(111) and from 1.84 to 0.13 (2.10 to 0.50) eV for Si(110). ΔLUMO exhibits a positive shift of approximately 0.5 eV on Si(111) compared to Si(110), except for the ΔLUMO of C_4_, where the shift is more pronounced, while ΔHOMO exhibits an approximately constant shift of 0.5 eV in the opposite direction,

These results confirm our previous findings: for alkenyl moieties, electron and hole tunneling barriers decrease with chain length, also on Si(110). The HOMO level of alkenyl carbon chains longer than C_10_ is close to the valence band edge. The negative value of ΔHOMO for C_18_ means that the HOMO energy level is in the bulk silicon band gap. Therefore, the coupling between the moiety and the surface is enhanced with increasing alkenyl chain length due to improved energy level alignment. Examples of energy levels schematics are reported in Figures S58 and S59 for C_18_ moieties on hydrogenated Si(111) and Si(110) surfaces. Surface orientation also influences the barrier heights, though to a lesser extent. Overall, both molecular structure and surface can be exploited to control charge injection, providing useful guidelines for the design of optimized electronic devices. While nonlocal functionals cannot directly predict quasiparticle levels, the experimental electron tunneling barrier of the C_9_ alkenyl moiety found to be around 1 eV^58^ shows remarkable consistency with our calculated barriers for the C_8_ and C_10_ homologues (1.2 eV and 1.0 eV, respectively).

### Charge Density Analysis

To gain deeper insight into the interaction between the substrate and the aliphatic moiety, we computed the Löwdin charge distribution. The analysis reveals an electronic charge transfer from the substrate to the molecular moiety, in line with our previous findings on the Si(111) surface.^56^ A positive charge accumulation is observed on the topmost Si atom of the surface bilayer, while a corresponding negative charge resides on the molecular atoms in proximity to the interface. Conversely, the terminal group farthest from the surface exhibits a small positive charge, yielding an overall dipole moment oriented away from the surface.

In order to understand the origin of the interfacial energetic results, we analyzed the charge density rearrangement induced by moiety adsorption. We focused on the systems containing C_8_ moieties adsorbed on Si(111) and Si(110), and evaluated the charge density difference obtained by subtracting the charge densities of the isolated slab and isolated moiety, each frozen in the adsorption geometry, from the charge density of the combined system. The plane averaged quantity was computed as a function of the z-coordinate. The results are shown in Figure 7.

**Figure 7:**
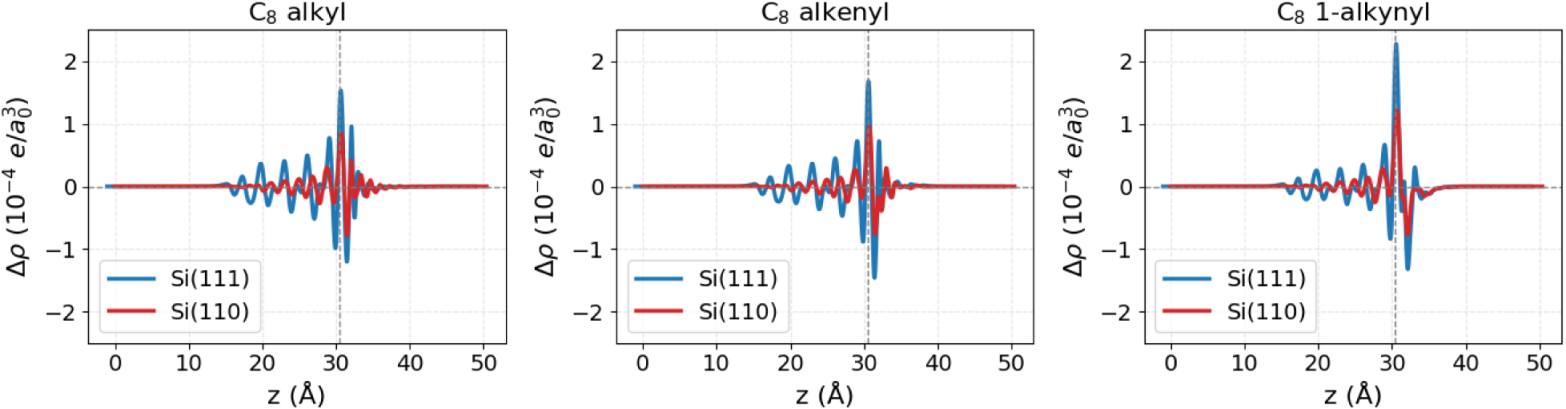
Difference in charge density between the combined system and the sum of the isolated moiety and slab with hydrogen vacancy for C_8_ alkyl (left), alkenyl (center), and 1-alkynyl (right). Results for H–Si(111) are shown in blue and for H–Si(110) in red (e is the electron charge and a_0_ is the Bohr radius).

For all three moieties, the larger peak amplitudes observed on Si(111) compared to Si(110) indicate a more pronounced charge displacement on the former surface. This behavior is reflected in the systematically larger dipoles observed on the Si(111) surface. The difference in dipolar response between the two surfaces can be attributed to their distinct geometries, as described in the structural section. The different arrangement of Si atoms is reflected in the different strength of the dipole induced in the adsorbed linear molecule. Indeed Si(110) has chains of silicon atoms passivated by tilted Si–H alternated to grooves; Si(111) has Si bilayer with top Si passivated by perpendicular Si–H bonds forming a regular and more densely packed structure.

On H–Si(110), due to the zigzag and less dense arrangement, the local electrostatic potential in the plane parallel to the surface is reduced in correspondence of the grooves as shown in Figure 8; the net dipole is diluted both by geometry and by lower density. On the other side, higher packing density and uniform orientation on Si(111) produces an equally uniform potential (also shown in Figure 8) implying that the surface dipole per area is maximized (all pointing in the same direction, with minimal cancellation). Therefore, given the same linear moiety, H–Si(111) generates the most intense overall surface dipole due to its perpendicular and densely packed Si–H bonds. H–Si(110), with its chains and grooves, yields a less intense dipole.

**Figure 8:**
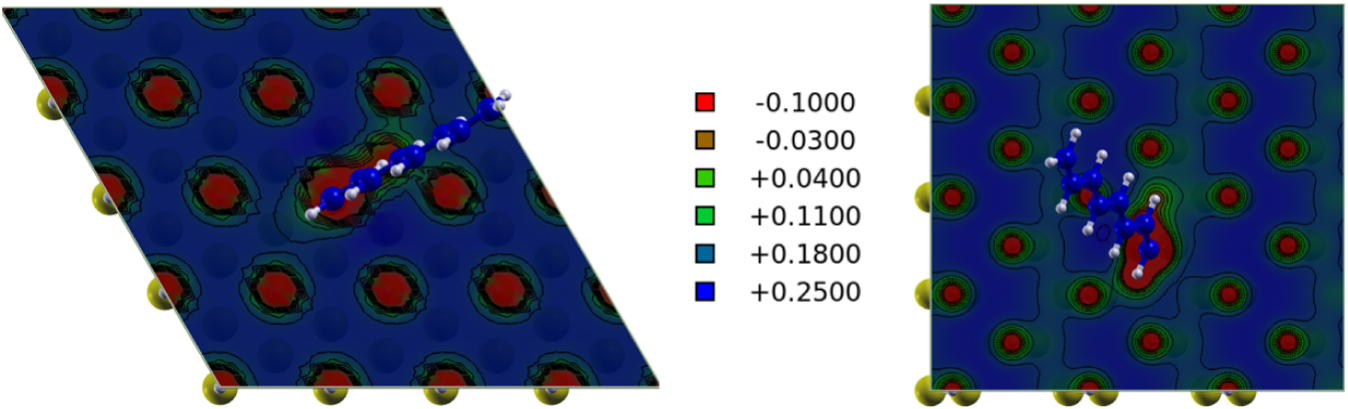
Surface map of the electrostatic potential energy in the plane including the C_1_ atom for C_8_ alkenyl moiety adsorbed on hydrogenated Si(111) (left) and Si(110) (right) surfaces. Rydberg units are used in the color map. The red (blue) colors indicate electron-rich (-poor) regions where a positive (negative) test charge is attracted.

The orbital analysis also allowed us to clarify the peculiar geometry adopted by alkenyl moieties adsorbed on Si(110), that is shown in the left panel of Figure 9 in the case of the C_6_ moiety. As described in the structural section, in this case the molecular plane is tilted with respect to the surface. To understand the origin of this configuration, we analyzed the occupied molecular orbitals of the organic/inorganic interface and observed the hybridization between the π-orbital perpendicular to the plane of the moiety and the surface orbital in the Γ point at the energy corresponding to the first PDOS resonance peaks of Si and C below the valence band edge. The hybridized molecular orbital (associated to the occupied band N_VB_-28, where N_VB_ is the number of the valence band) is shown in the right panel of Figure 9, where its bonding character may be observed between the C_3_–C_4_ bond of the moiety and one of the hydrogen atoms passivating the surface. This results in the tilted stacking of the molecular plane over the surface, confirming the charge transfer that stabilizes the tilted geometry, making it energetically favorable.

**Figure 9:**
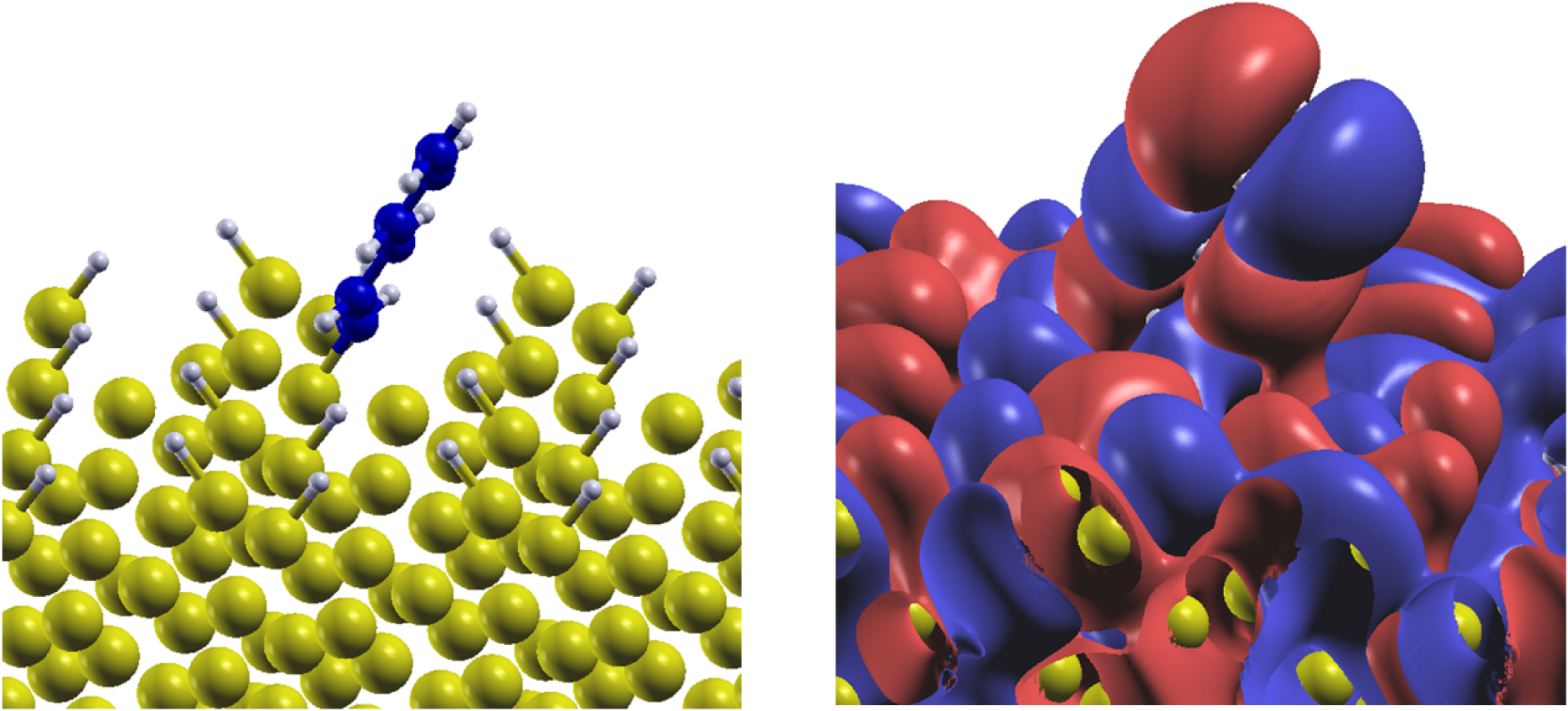
Left: adsorption geometry. Right: corresponding molecular orbitals of C_6_ moiety adsorbed on Si(110) associated to the occupied band N_VB_-28, where N_VB_ is the number of the valence band, and to the Γ point.

## Conclusions

In conclusion, we extended our previous studies of aliphatic chain functionalization on H–Si(111) surfaces to include longer chains (C_12_–C_18_) and a comparative analysis with the H–Si(110) crystallographic orientation. The systematic study of alkyl, alkenyl, and 1-alkynyl moieties revealed fundamental insights into the principles for controlling interfacial electronic properties in silicon-based molecular devices.

Among the three moiety types investigated, alkenyl chains exhibit the most pronounced sensitivity to chain length, with surface dipole energy steps varying continuously and significantly in the range 120–410 meV on Si(111) at the given coverage, making them particularly attractive candidates for systematic tuning of interface energetics. In contrast, alkyl and 1-alkynyl moieties display saturation behavior for chains longer than C_8_. This suggests that the interfacial dipole is mainly established through interactions localized near the Si–C bond and the immediate molecular environment.

Crystallographic surface orientation is a critical parameter: H–Si(111) consistently generates surface dipoles that exceed those on H–Si(110) by up to 33%. This difference is rooted in the distinct atomic arrangements: the high-density, perpendicular Si–H bond network on Si(111) produces a more coherent and intense surface dipole per unit area, whereas the corrugated geometry of Si(110) with its tilted H bonds and lower packing density generates weaker dipole contributions. This structural origin is supported by charge density analysis and should be considered in the design of the strategies for optimizing device performance through surface selection.

The analysis of tunneling barriers demonstrates that alkenyl moieties enhance coupling of molecular frontier orbitals with silicon band edges for chain lengths beyond C_10_, a feature not observed in alkyl and alkynyl terminations. The systematic energy shifts of HOMO and LUMO levels with increasing chain length for alkenyl species provide a mechanism for engineering charge injection barriers and potentially enhancing charge transport in molecular electronic devices.

Finally, the molecular orbital analysis reveals a previously undescribed orientation mechanism: on Si(110), alkenyl moieties adopt a tilted geometry stabilized by hybridization between the π-orbitals perpendicular to the molecular plane and occupied Si surface states. This hybridization-driven configuration represents an example of how molecular geometry and interface electronic structure become intimately coupled at the organic–inorganic interface.

Our results demonstrate that systematic variation of the molecular structure (moiety type and chain length) and of the substrate orientation (Si(111) vs Si(110)) allows interfacial dipoles and charge injection barriers to be tuned over ranges relevant for specific target applications. Future computational work should focus on the adsorption of multiple moieties forming monolayers at finite molecular coverage, to incorporate intermolecular interactions that are expected to further modulate the electronic properties reported here. Additionally, investigation of different terminal functional groups could expand the set of strategies available for interface engineering. Such developments would pave the way toward the rational design of silicon-based molecular electronics, biosensors, and photovoltaic devices.

## Supplementary Material



## Notes

Figures representing the minimal energy configurations of adsorption geometries for C_8_ moieties, Projected Density of States (PDOS), plane-averaged electrostatic potential, energy level schematics, and Electron Localization Function (ELF); Table reporting ab initio dipole values as a function of supercell size (PDF)
