# Supplementary material for: Interfacial Energetics of C2–C18 Aliphatic Moieties on Hydrogenated Si(111) and Si(110) Surfaces: A DFT Study
Source: Langmuir. 2026 May 3;42(19):13618–27. doi: 10.1021/acs.langmuir.6c00727 (PMC13192326; doi:10.1021/acs.langmuir.6c00727)
Supplement: Supplementary file 1 [file la6c00727_si_001.pdf]

Interfacial Energetics of C_2–C_{18} Aliphatic Moieties on Hydrogenated Si(111) and Si(110) surfaces: a DFT Study

Sara Marchio[1,$], Francesco Buonocore,[1,$,*] Simone Giusepponi[1], Massimo Celino[1]

[1] Italian National Agency for New Technologies, Energy and Sustainable Economic Development (ENEA) – C. R. Casaccia, Via Anguillarese 301, 00123 Rome, Italy

[*] Corresponding author (francesco.buonocore@enea.it).

[$] S.M. and F.B. contributed equally to this paper.

Abstract

We present a computational study based on density functional theory to systematically investigate how aliphatic moiety functionalization affects the interfacial electronic structure of H-terminated Si(111) and Si(110) surfaces. We explore the energetics, dipole formation, and charge transfer mechanisms for alkyl, alkenyl, and 1-alkynyl chains containing from 2 to 18 carbon atoms chemisorbed on both crystallographic orientations. Our analysis reveals that alkenyl moieties exhibit pronounced chain-length dependence of surface dipoles and tunneling barriers, whereas alkyl and 1-alkynyl chains show saturation effects for longer chains. We found that H–Si(111) exhibits surface dipoles up to 33% larger than H–Si(110), due to differences in atomic packing density and Si-H bond orientation. The resulting charge injection barriers for both thermionic and tunneling transport are quantified and discussed. The tilted geometry adopted by alkenyl moieties on Si(110) is rationalized through analysis of molecular orbital hybridization with surface states. These results provide quantitative guidelines for engineering interface energetics in silicon-based molecular electronic devices through rational choice of molecular termination and substrate orientation.

Supporting Information

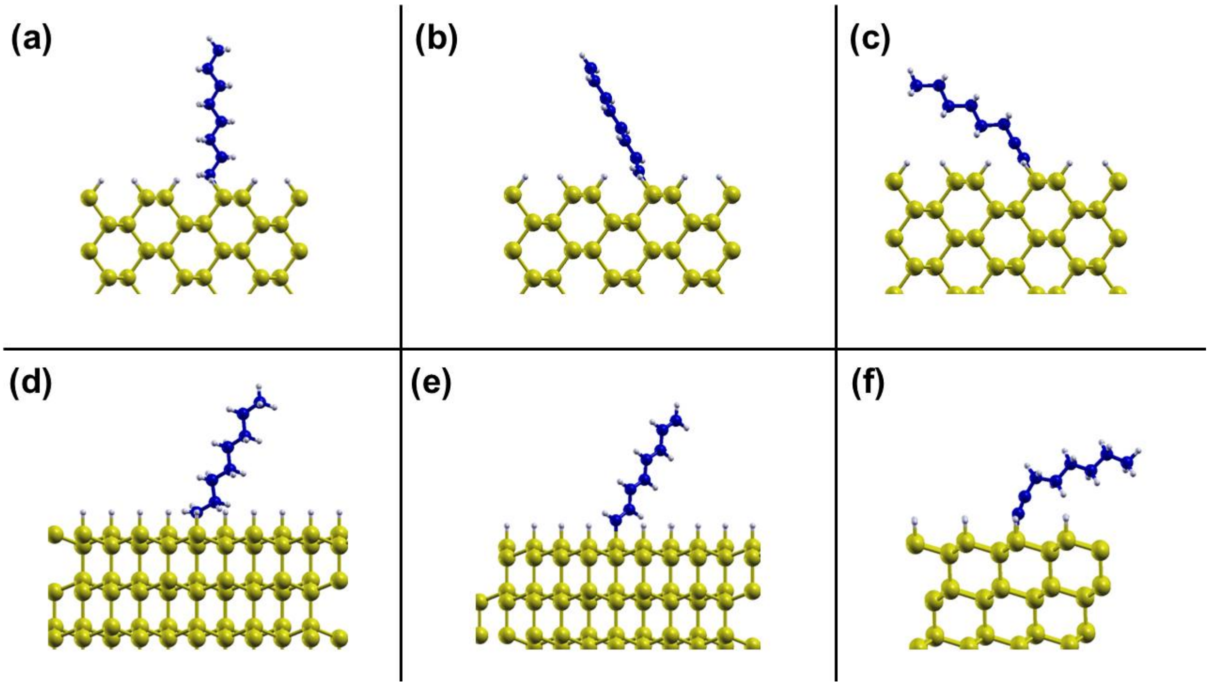

Figure S1. Adsorption geometries for C_8 alkyl (a,d), C_8 alkenyl (b,e), C_8 1-alkynyl (c,f) moieties on Si(110) (a-c) and Si(111) (d-f).

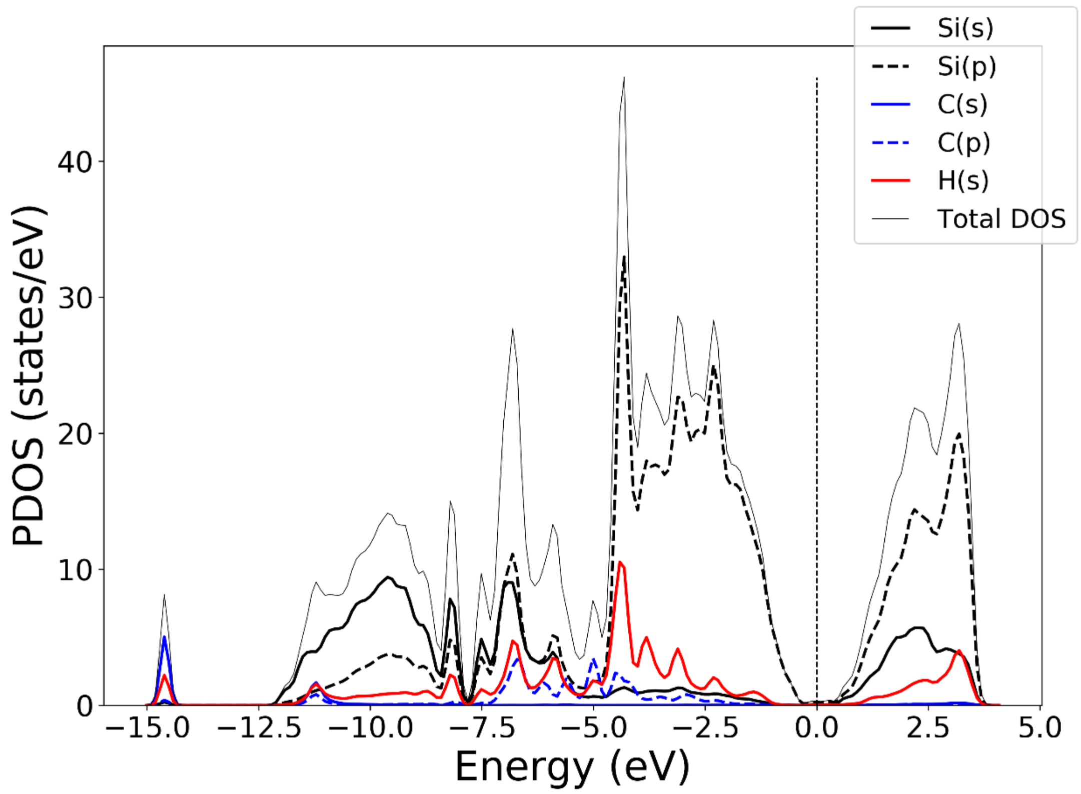

Figure S2. Projected density of states (PDOS) of C_2 alkyl moiety adsorbed on H−Si(111) slab.

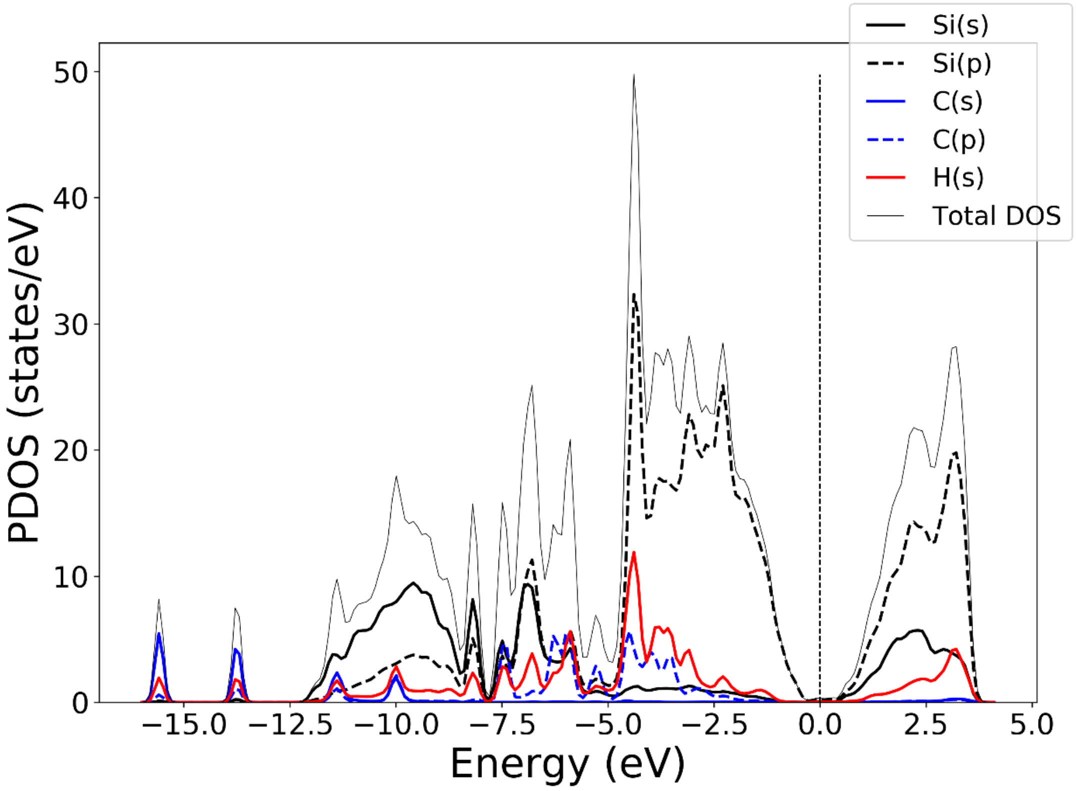

Figure S3. Projected density of states (PDOS) of C_4 alkyl moiety adsorbed on H−Si(111) slab.

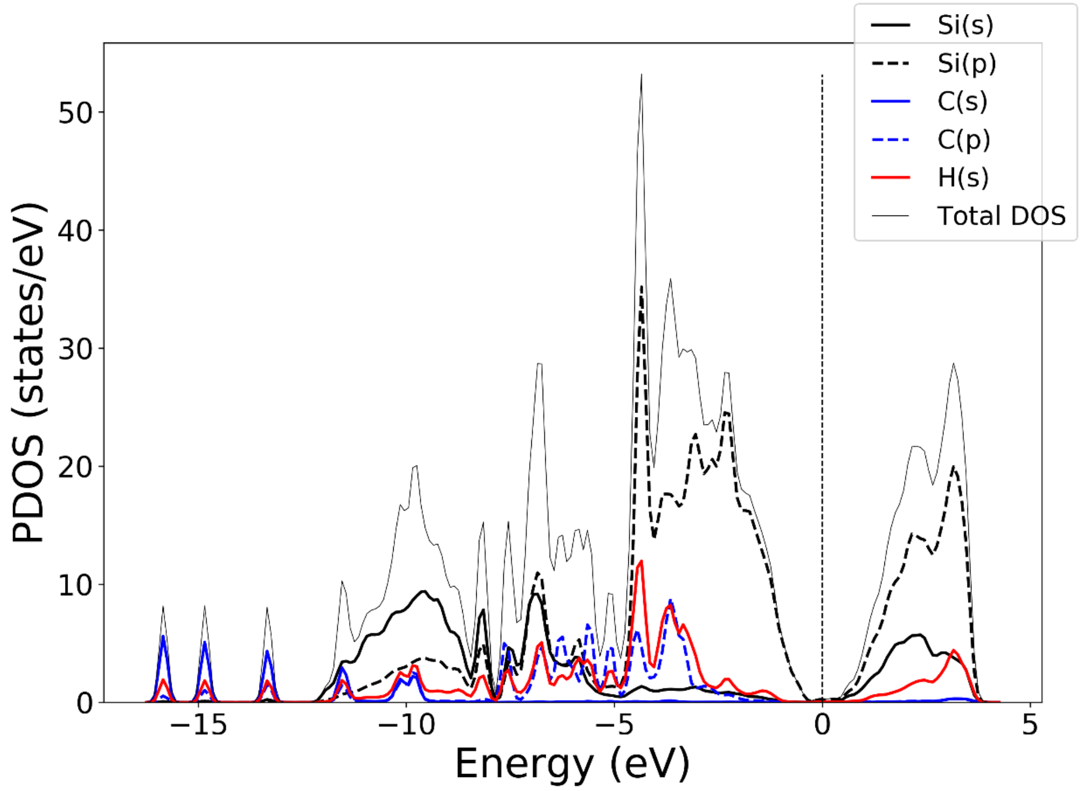

Figure S4. Projected density of states (PDOS) of C_6 alkyl moiety adsorbed on H−Si(111) slab.

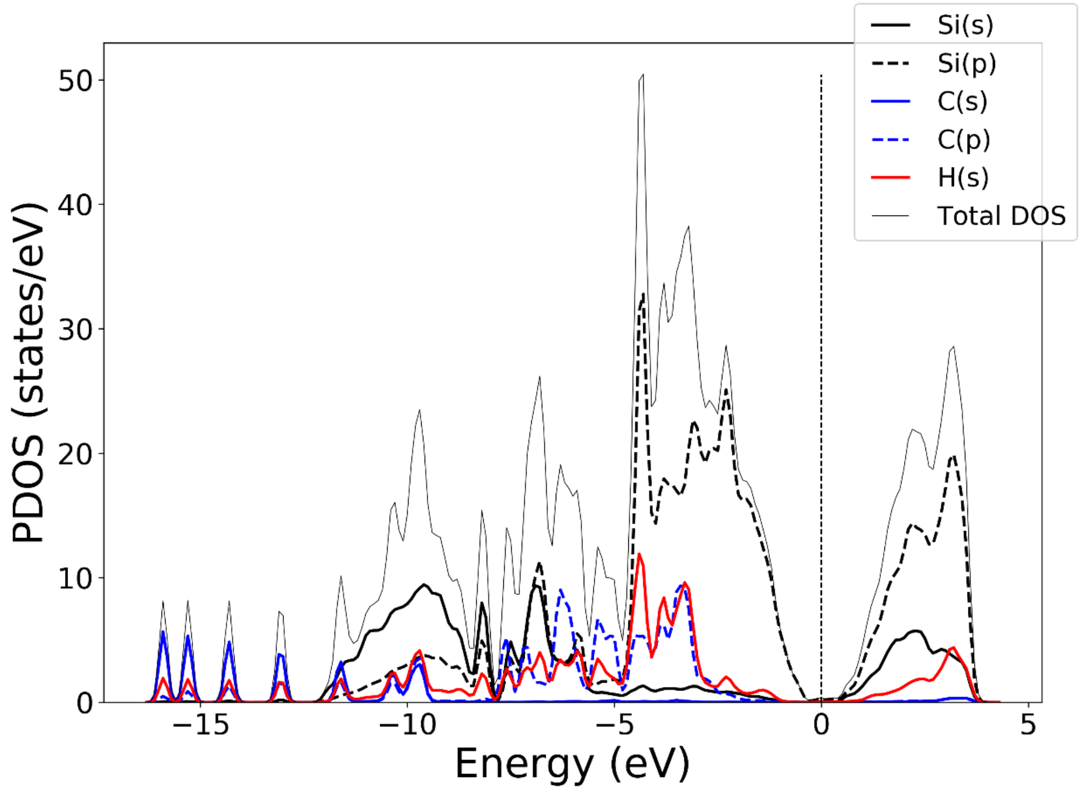

Figure S5. Projected density of states (PDOS) of C_8 alkyl moiety adsorbed on H−Si(111) slab.

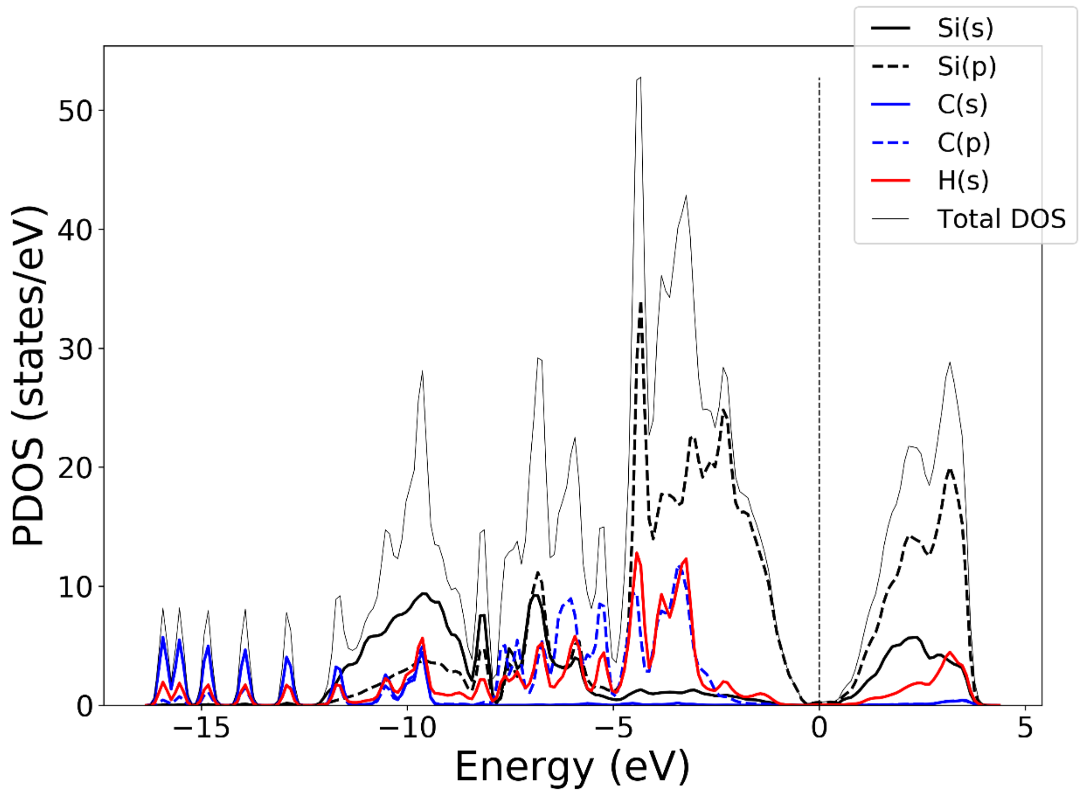

Figure S6. Projected density of states (PDOS) of C_{10} alkyl moiety adsorbed on H−Si(111) slab.

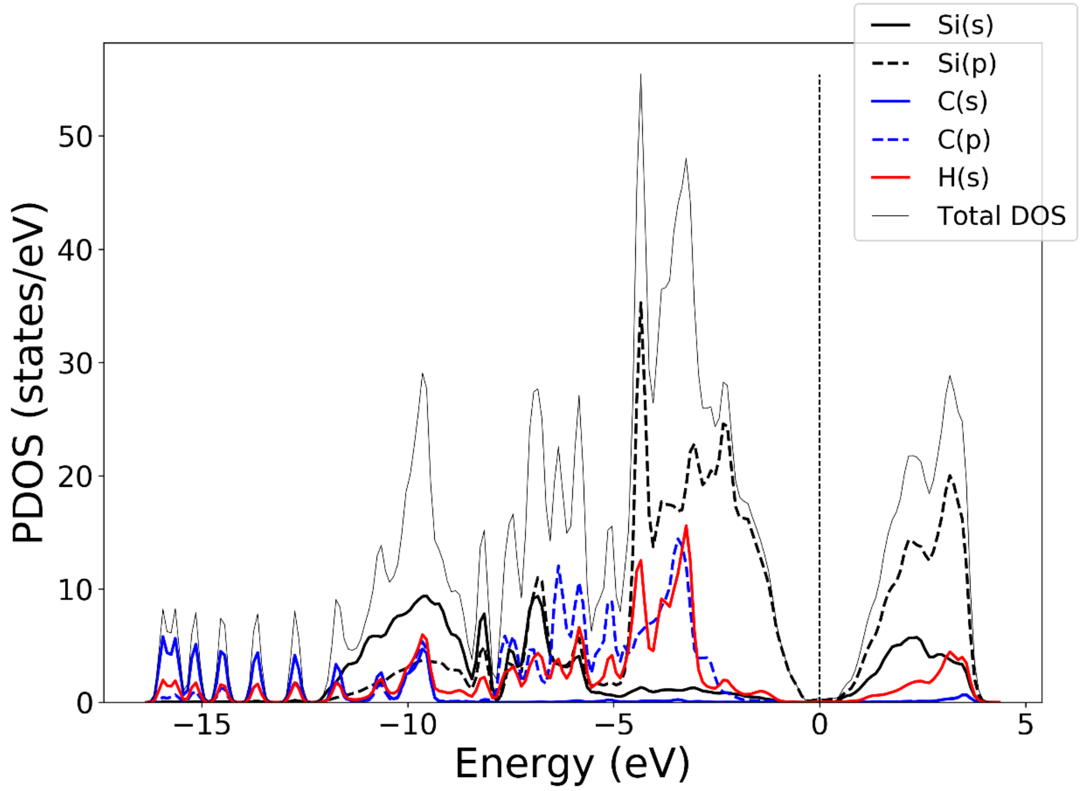

Figure S7. Projected density of states (PDOS) of C_{12} alkyl moiety adsorbed on H−Si(111) slab.

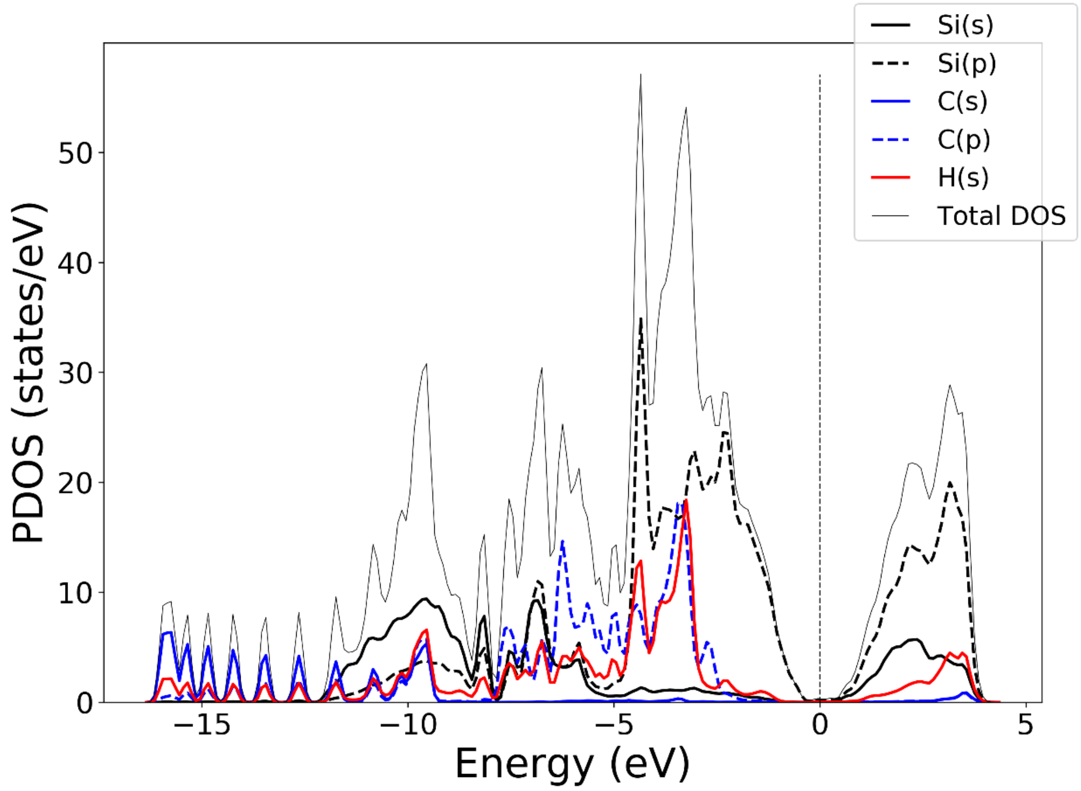

Figure S8. Projected density of states (PDOS) of C_{14} alkyl moiety adsorbed on H–Si(111) slab.

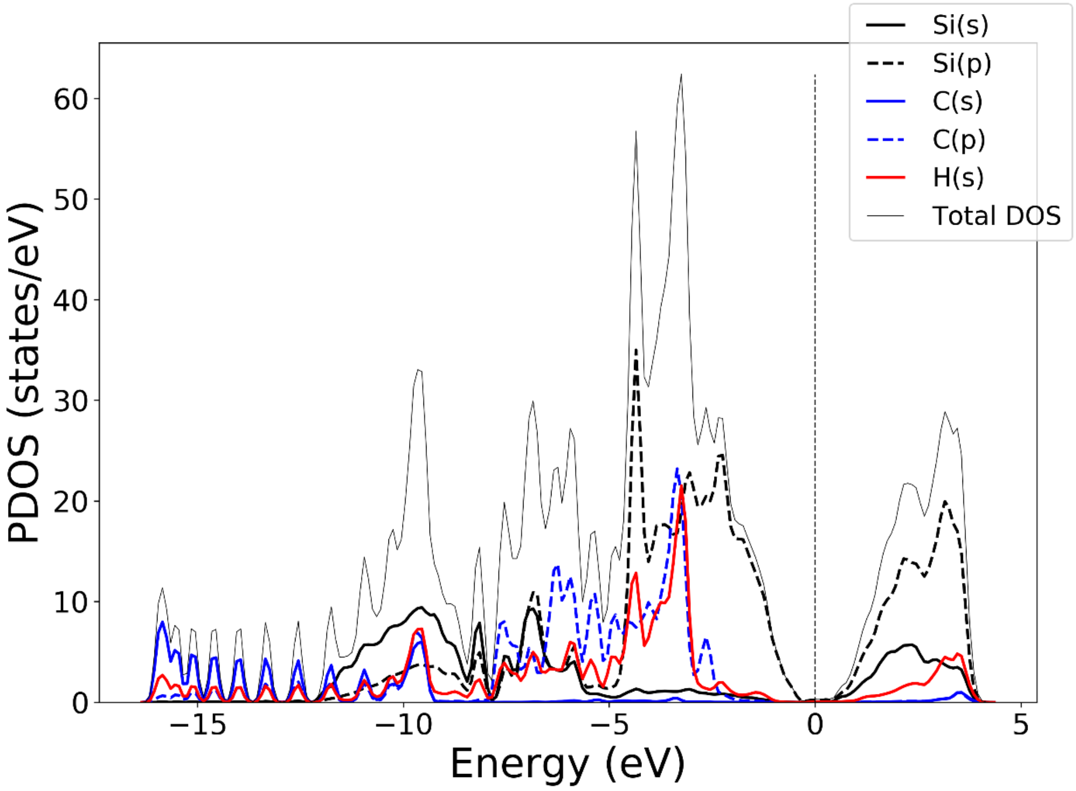

Figure S9. Projected density of states (PDOS) of C_{16} alkyl moiety adsorbed on H−Si(111) slab.

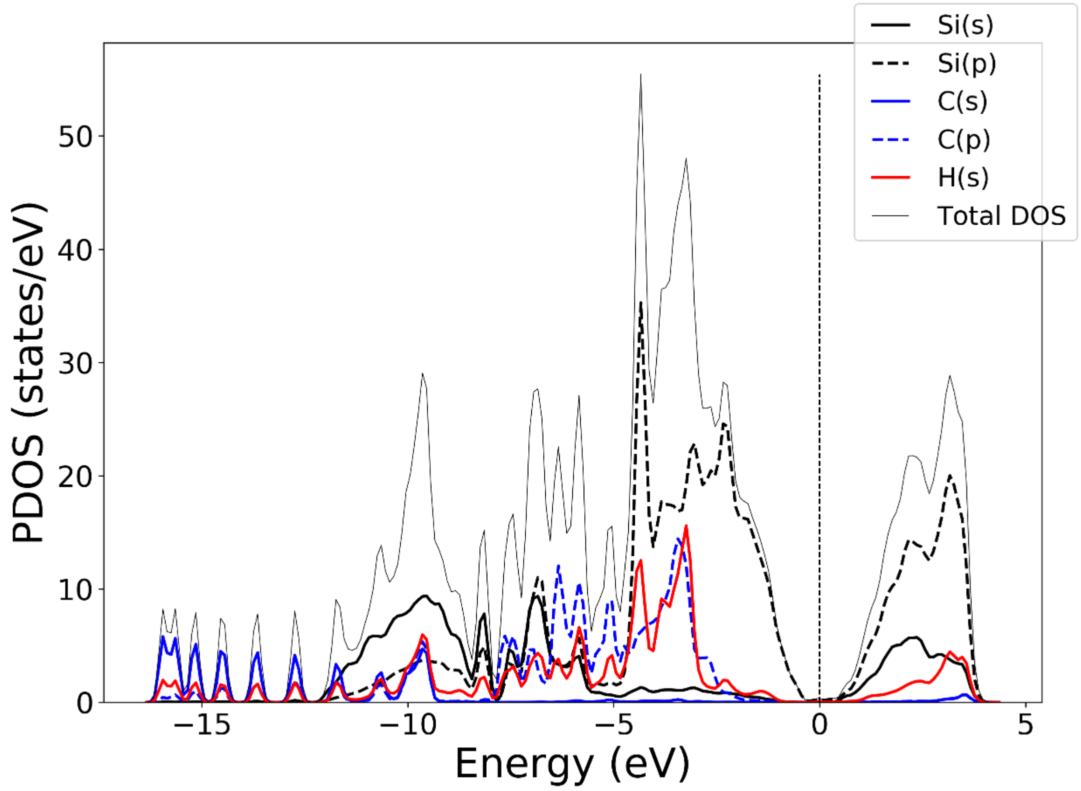

Figure S10. Projected density of states (PDOS) of C_{18} alkyl moiety adsorbed on H−Si(111) slab.

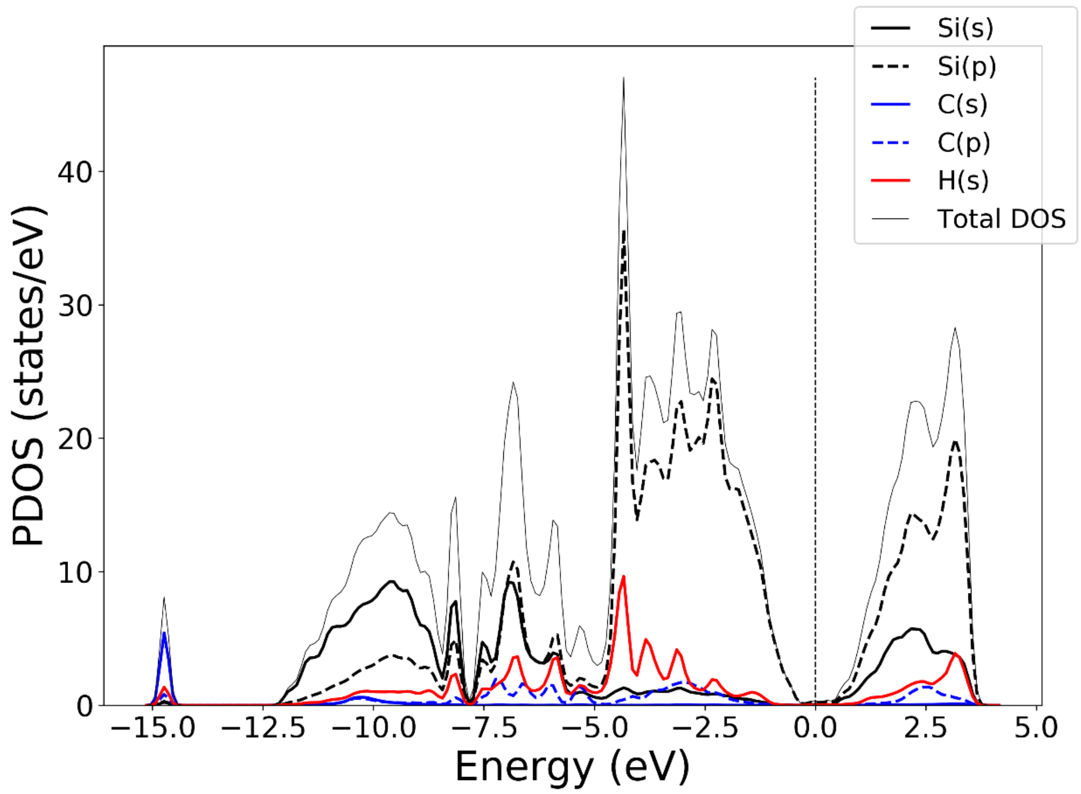

Figure S11. Projected density of states (PDOS) of C_2 alkenyl moiety adsorbed on H−Si(111) slab.

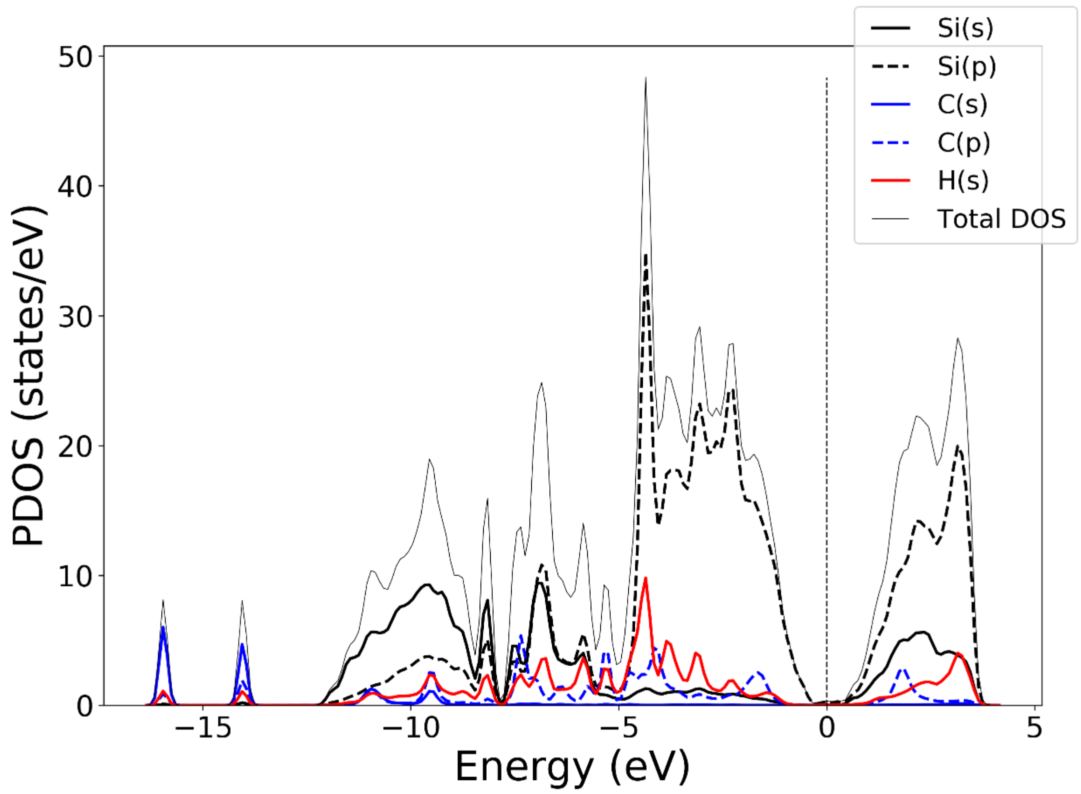

Figure S12. Projected density of states (PDOS) of C_4 alkenyl moiety adsorbed on H−Si(111) slab.

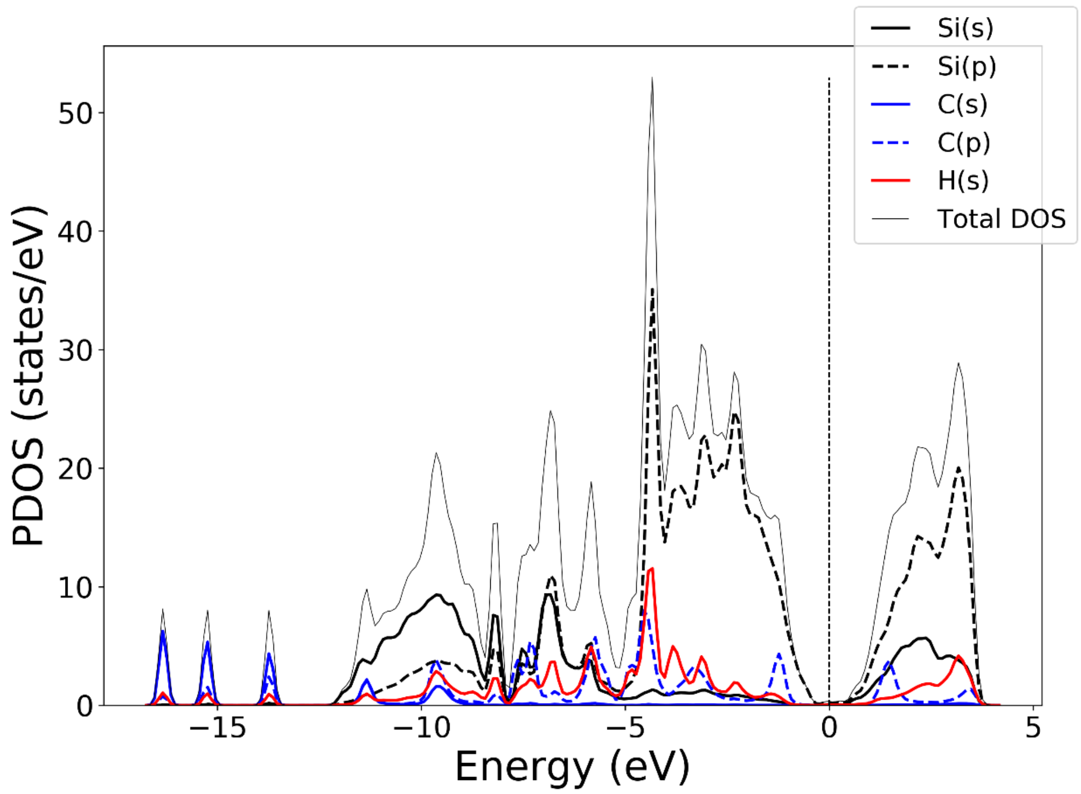

Figure S13. Projected density of states (PDOS) of C_6 alkenyl moiety adsorbed on H−Si(111) slab.

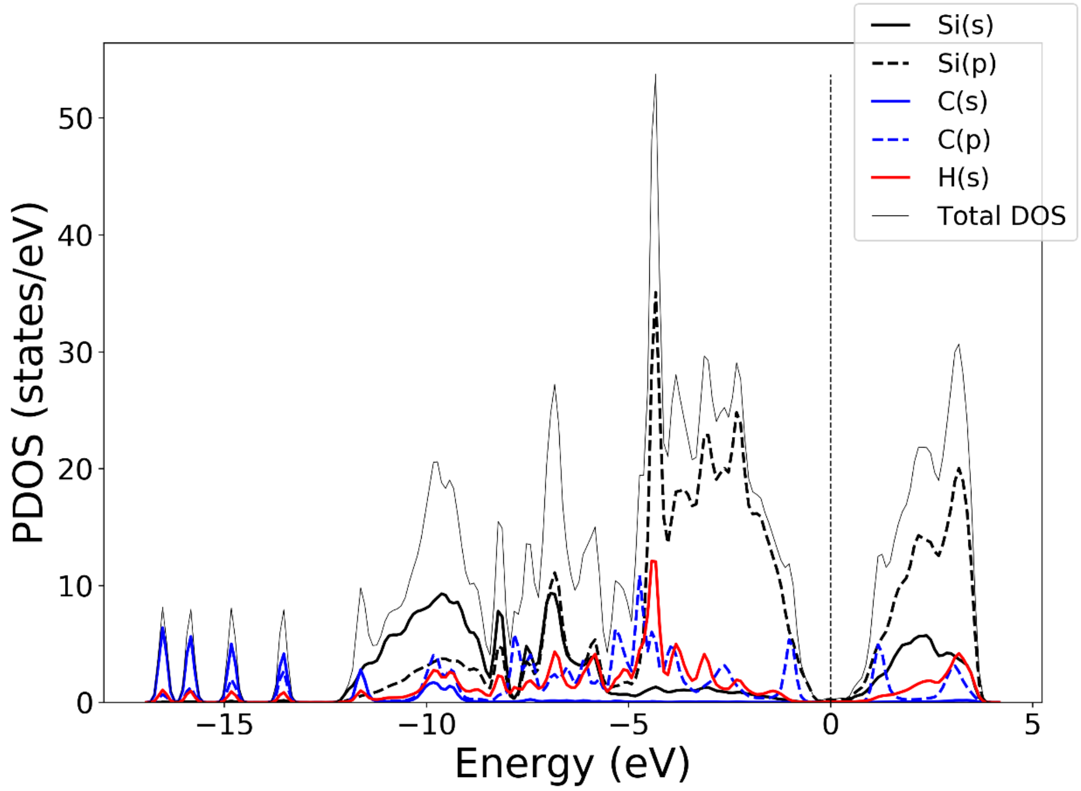

Figure S14. Projected density of states (PDOS) of C_8 alkenyl moiety adsorbed on H−Si(111) slab.

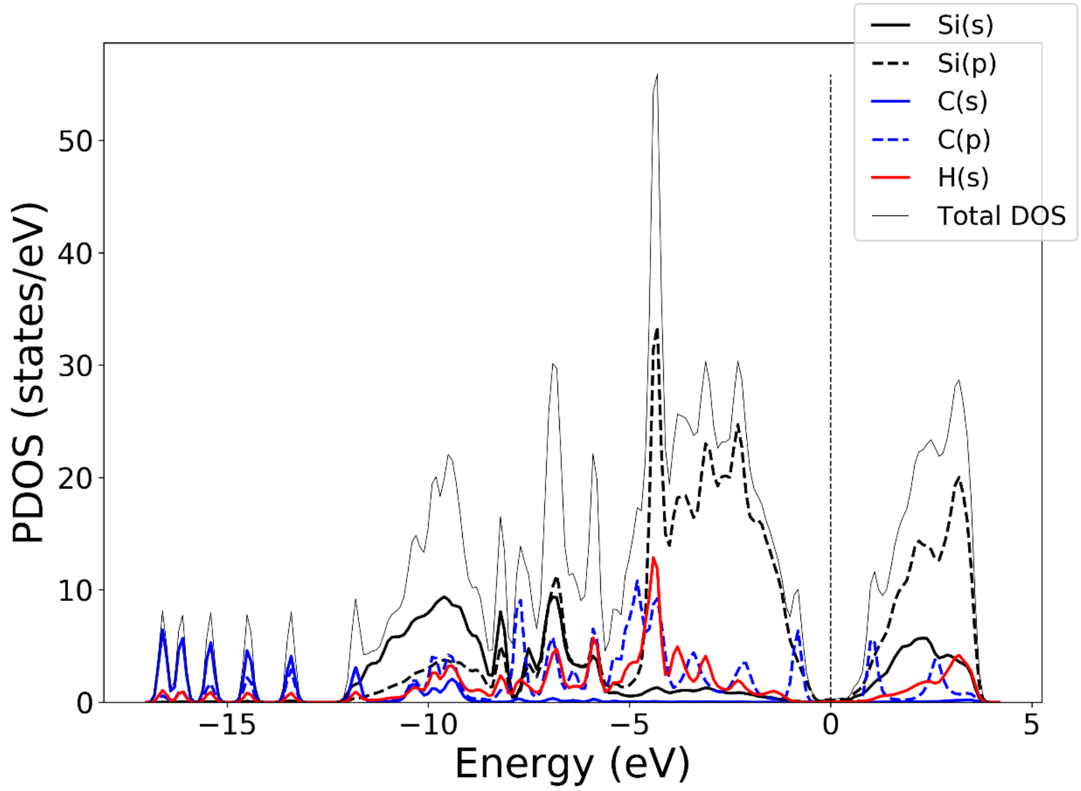

Figure S15. Projected density of states (PDOS) of C_{10} alkenyl moiety adsorbed on H−Si(111) slab.

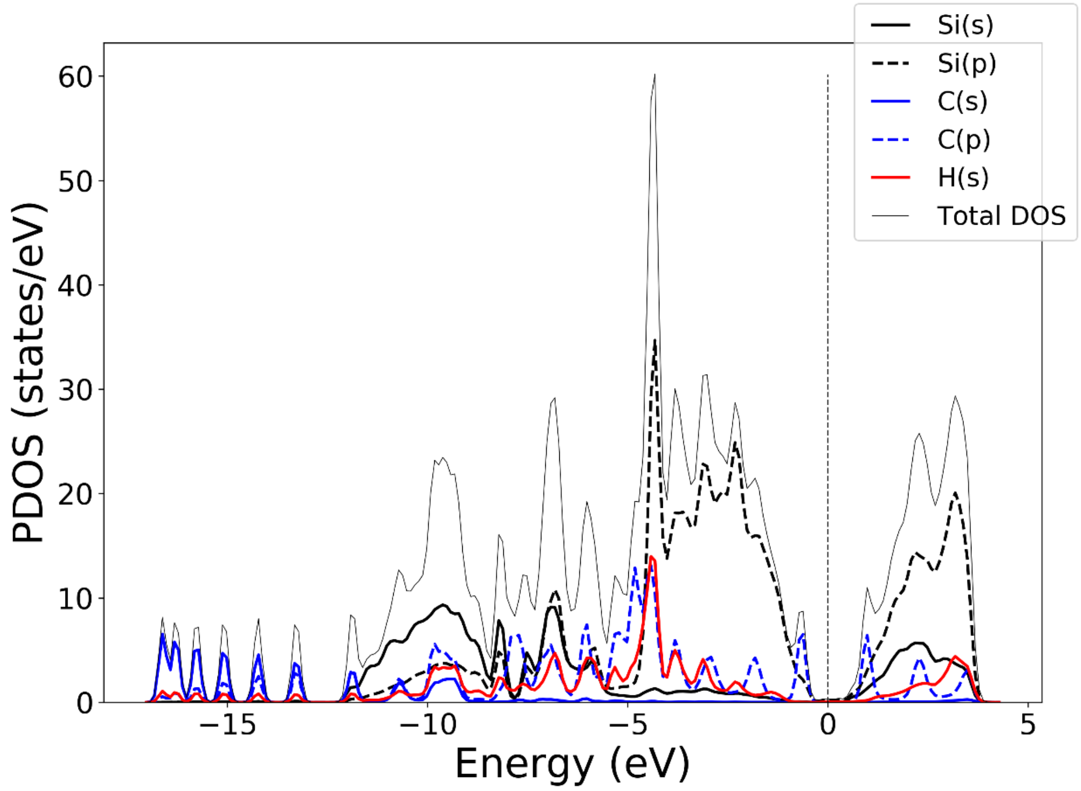

Figure S16. Projected density of states (PDOS) of C_{12} alkenyl moiety adsorbed on H−Si(111) slab.

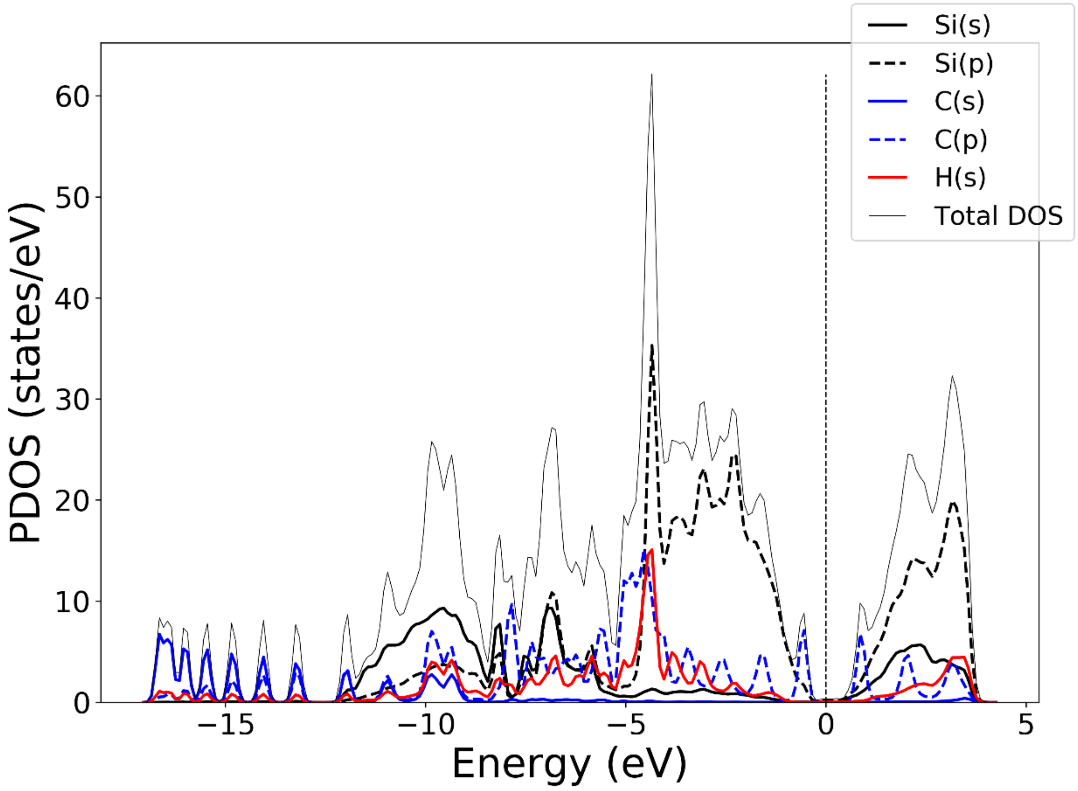

Figure S17. Projected density of states (PDOS) of C_{14} alkenyl moiety adsorbed on H−Si(111) slab.

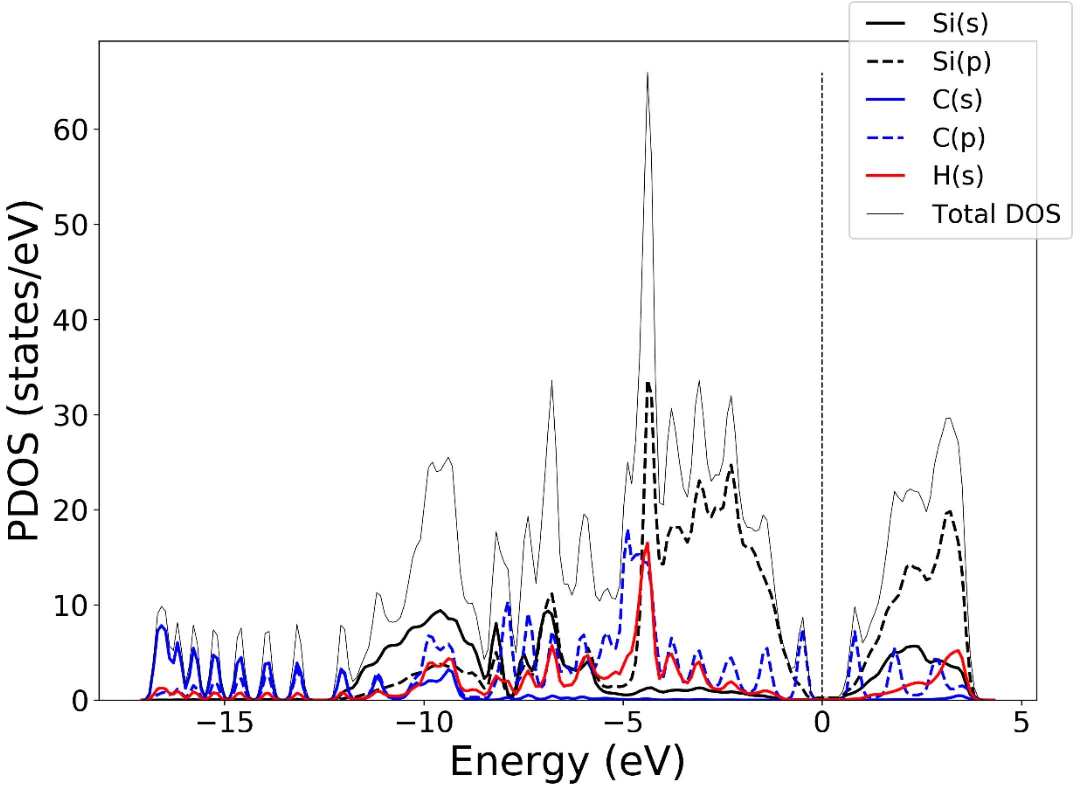

Figure S18. Projected density of states (PDOS) of C_{16} alkenyl moiety adsorbed on H−Si(111) slab.

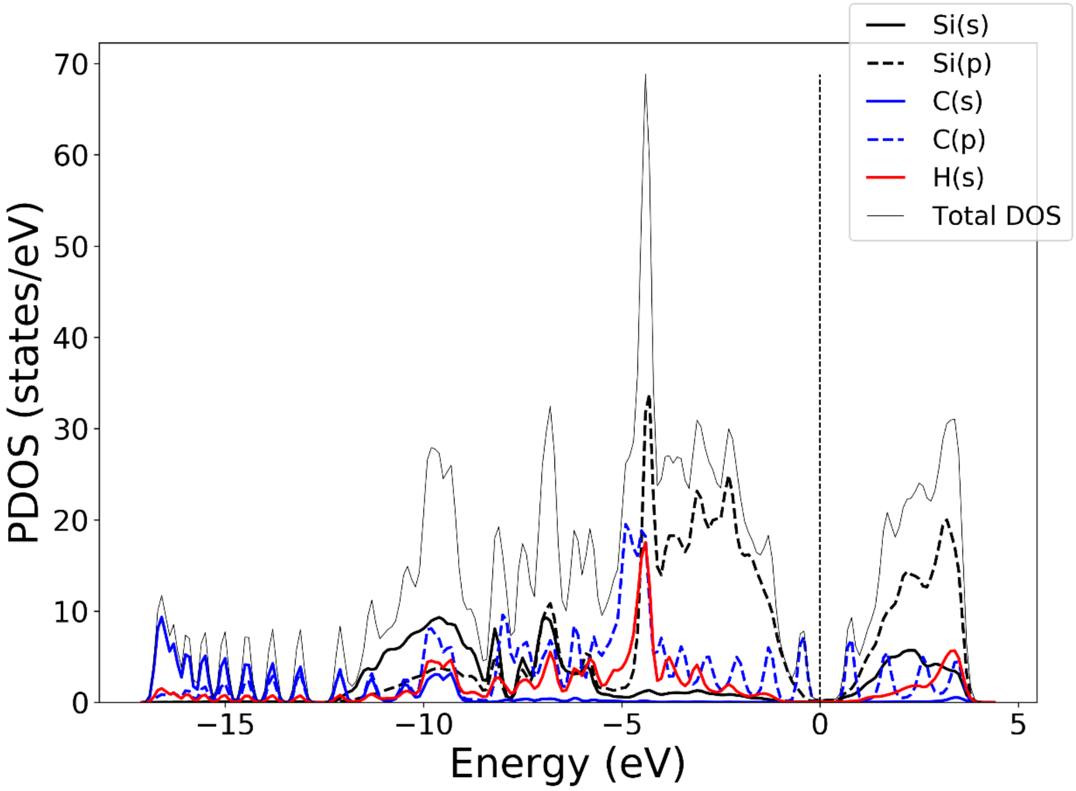

Figure S19. Projected density of states (PDOS) of C_{18} alkenyl moiety adsorbed on H−Si(111) slab.

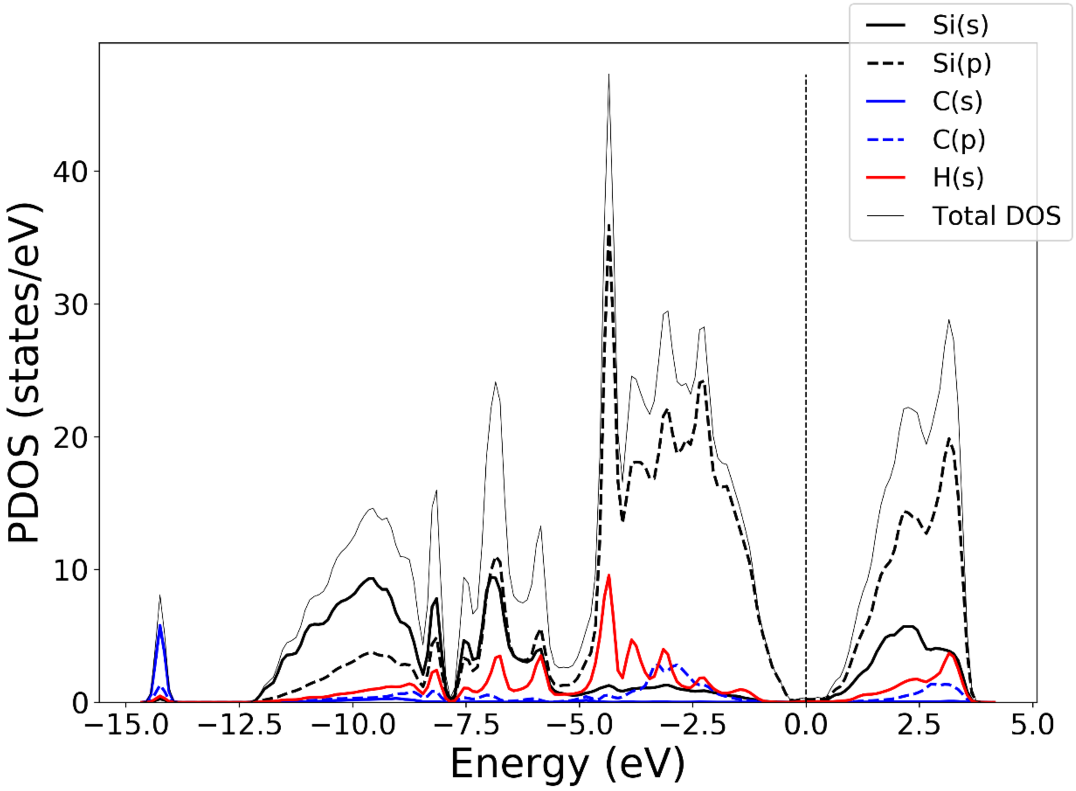

Figure S20. Projected density of states (PDOS) of C_2 1-alkynyl moiety adsorbed on H−Si(111) slab.

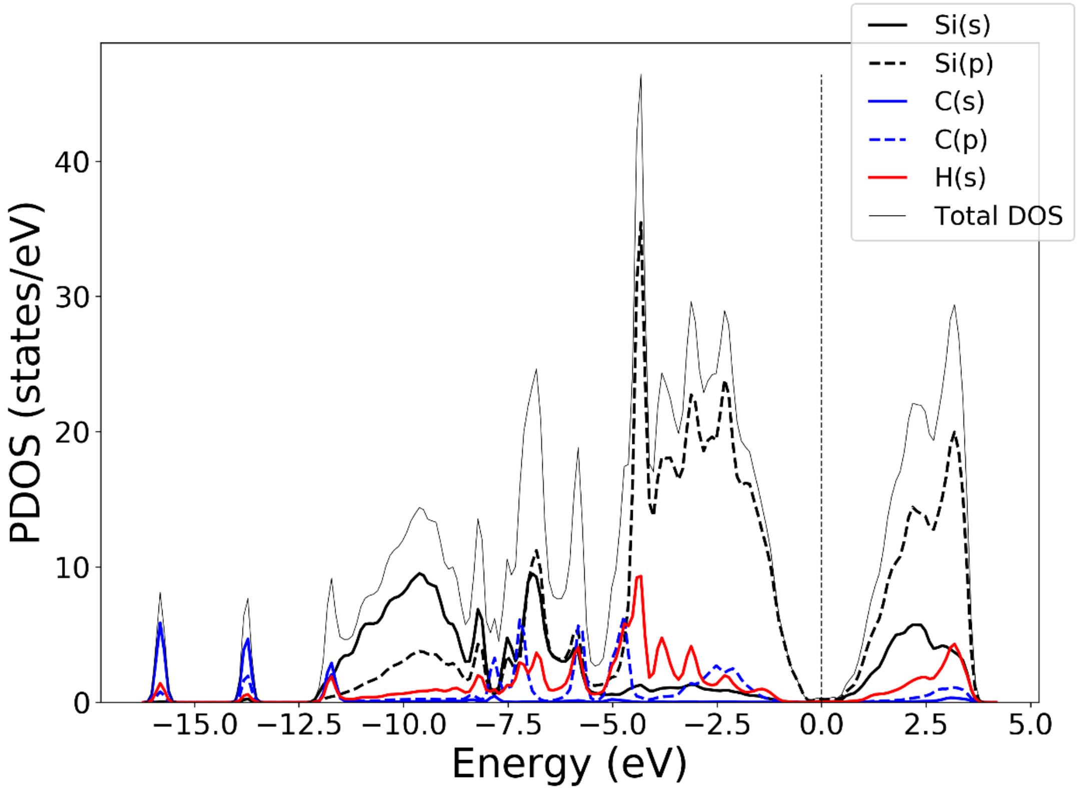

Figure S21. Projected density of states (PDOS) of C_4 1-alkynyl moiety adsorbed on H−Si(111) slab.

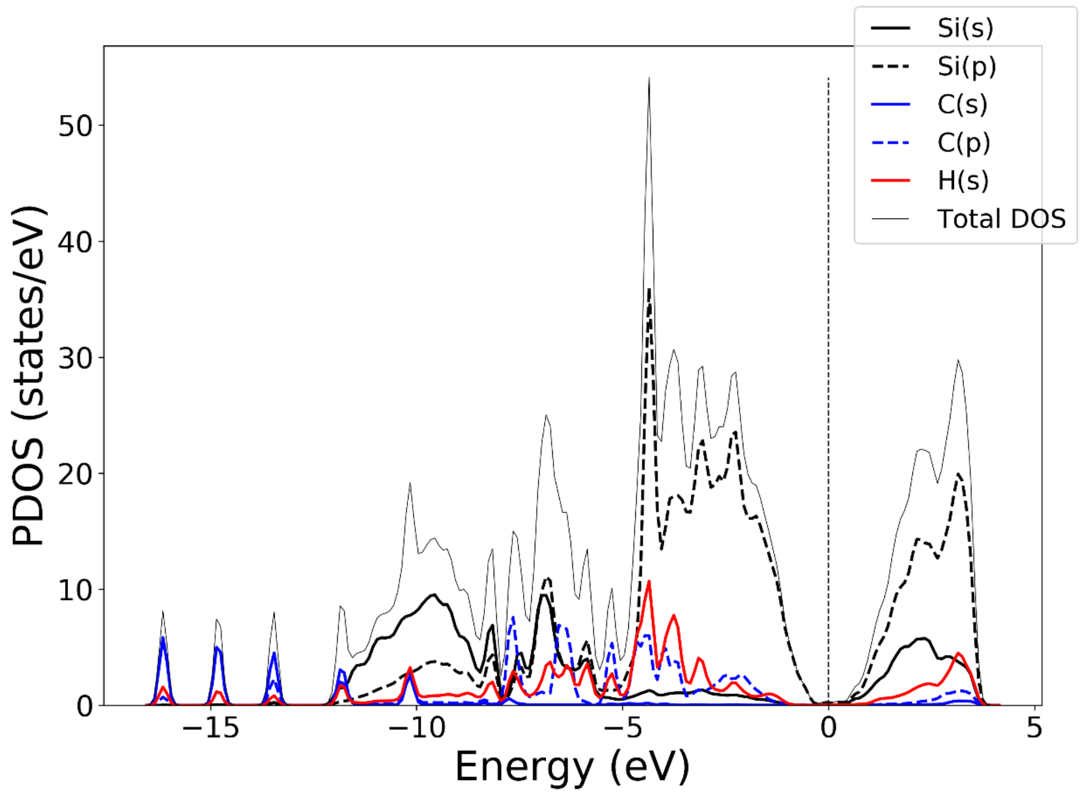

Figure S22. Projected density of states (PDOS) of C_6 1-alkynyl moiety adsorbed on H−Si(111) slab.

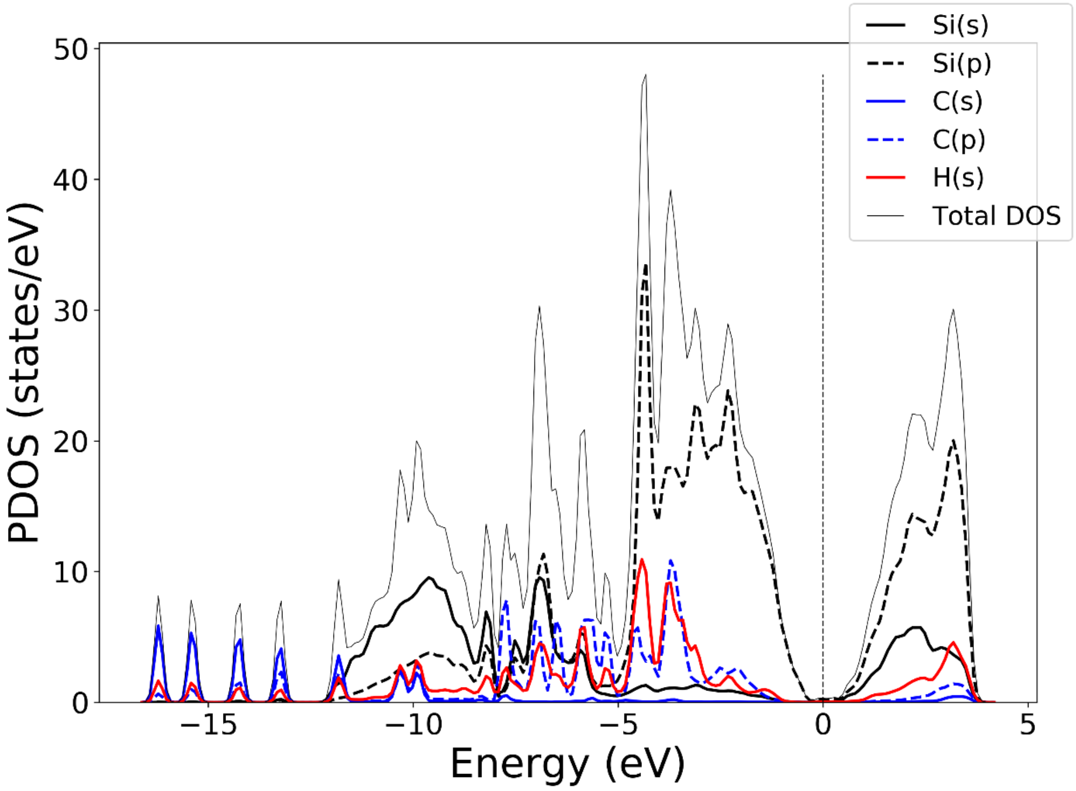

Figure S23. Projected density of states (PDOS) of C_8 1-alkynyl moiety adsorbed on H−Si(111) slab.

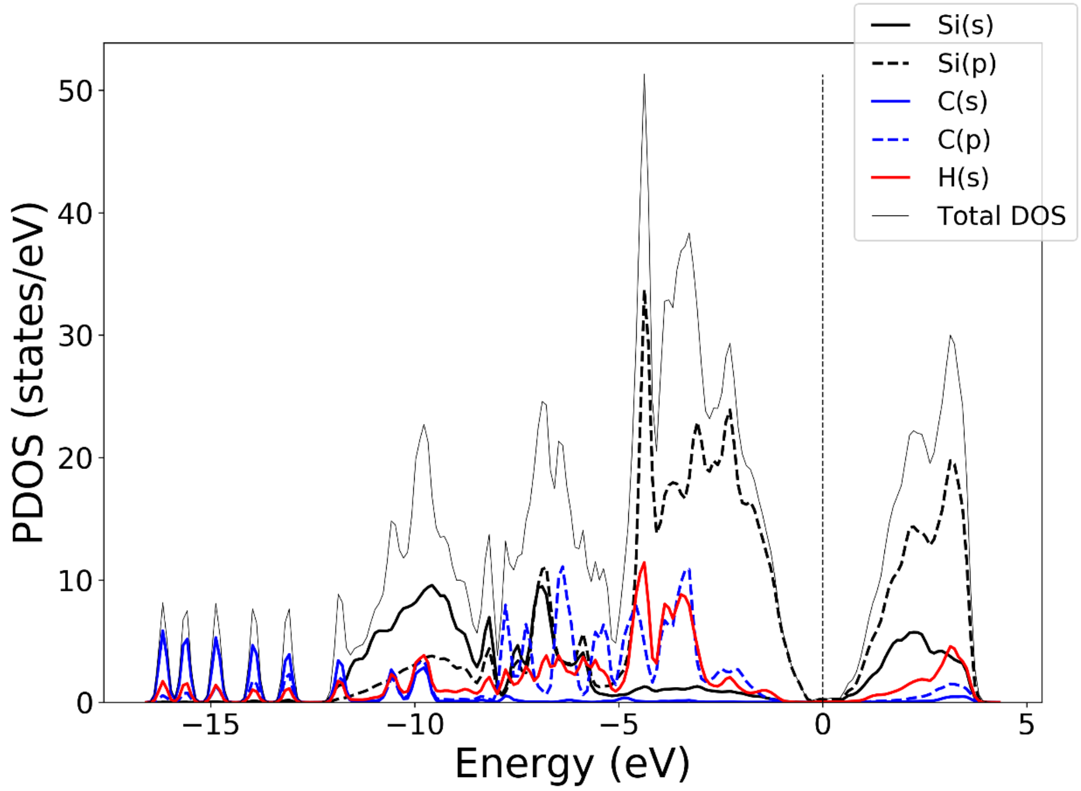

Figure S24. Projected density of states (PDOS) of C_{10} 1-alkynyl moiety adsorbed on H−Si(111) slab.

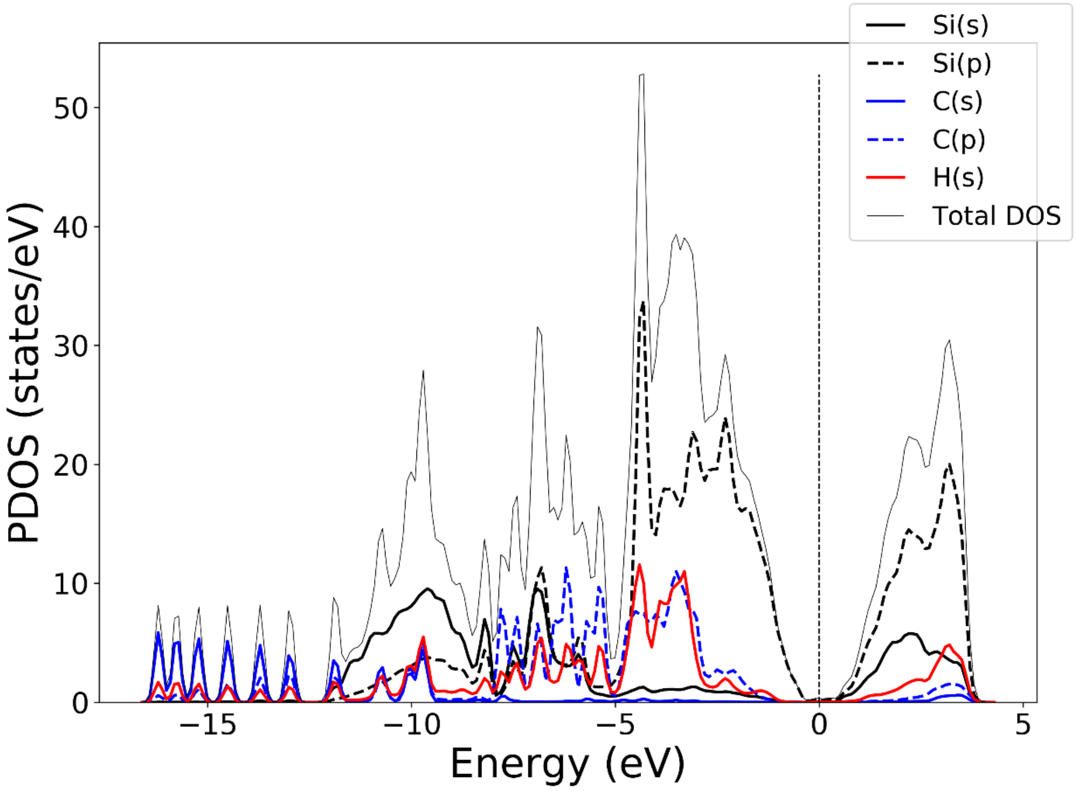

Figure S25. Projected density of states (PDOS) of C_{12} 1-alkynyl moiety adsorbed on H−Si(111) slab.

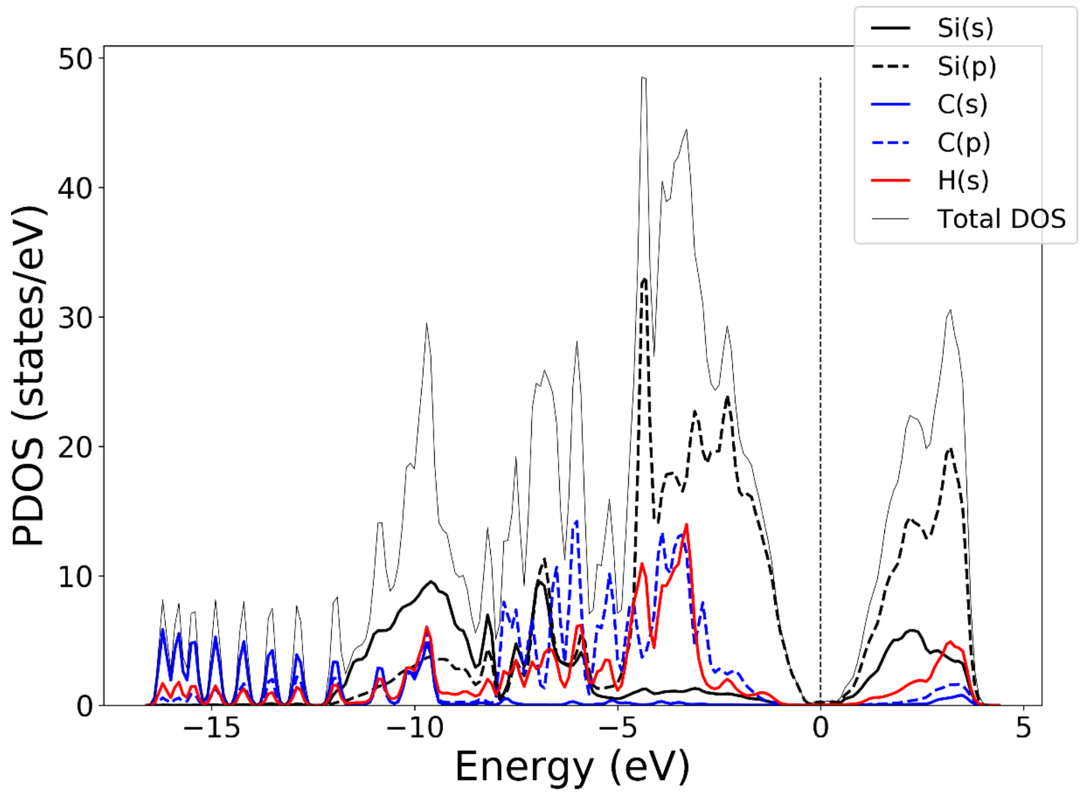

Figure S26. Projected density of states (PDOS) of C_{14} 1-alkynyl moiety adsorbed on H−Si(111) slab.

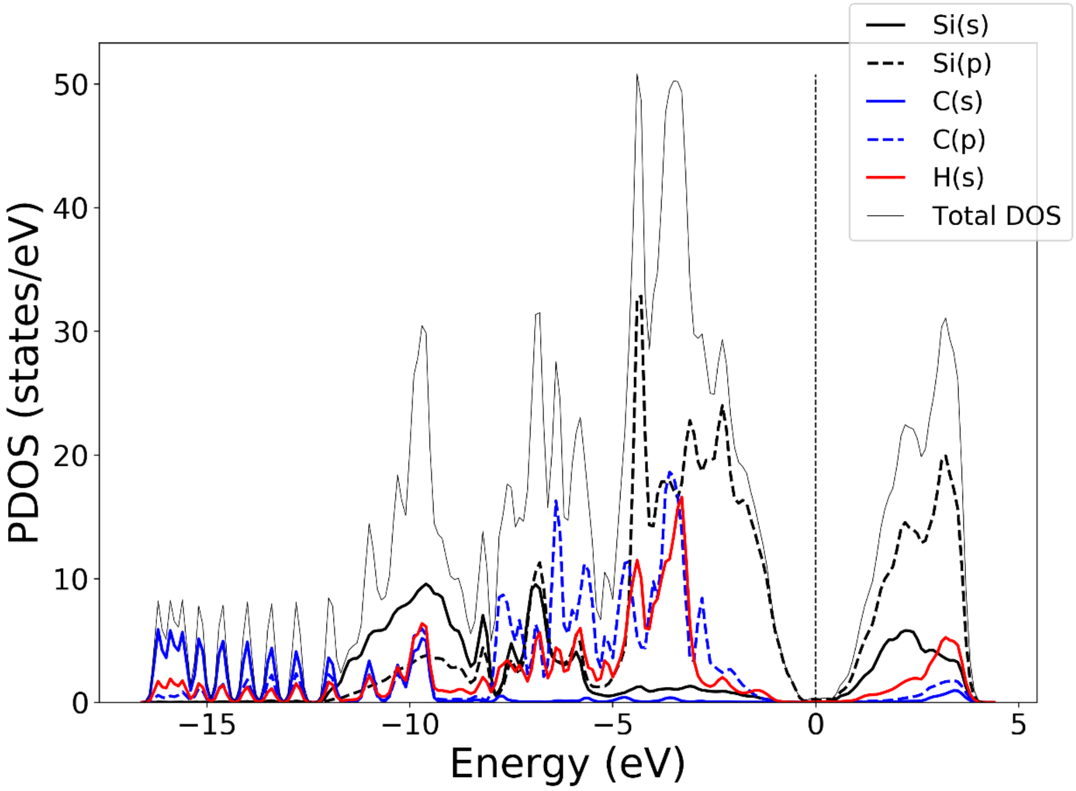

Figure S27. Projected density of states (PDOS) of C_{16} 1-alkynyl moiety adsorbed on H−Si(111) slab.

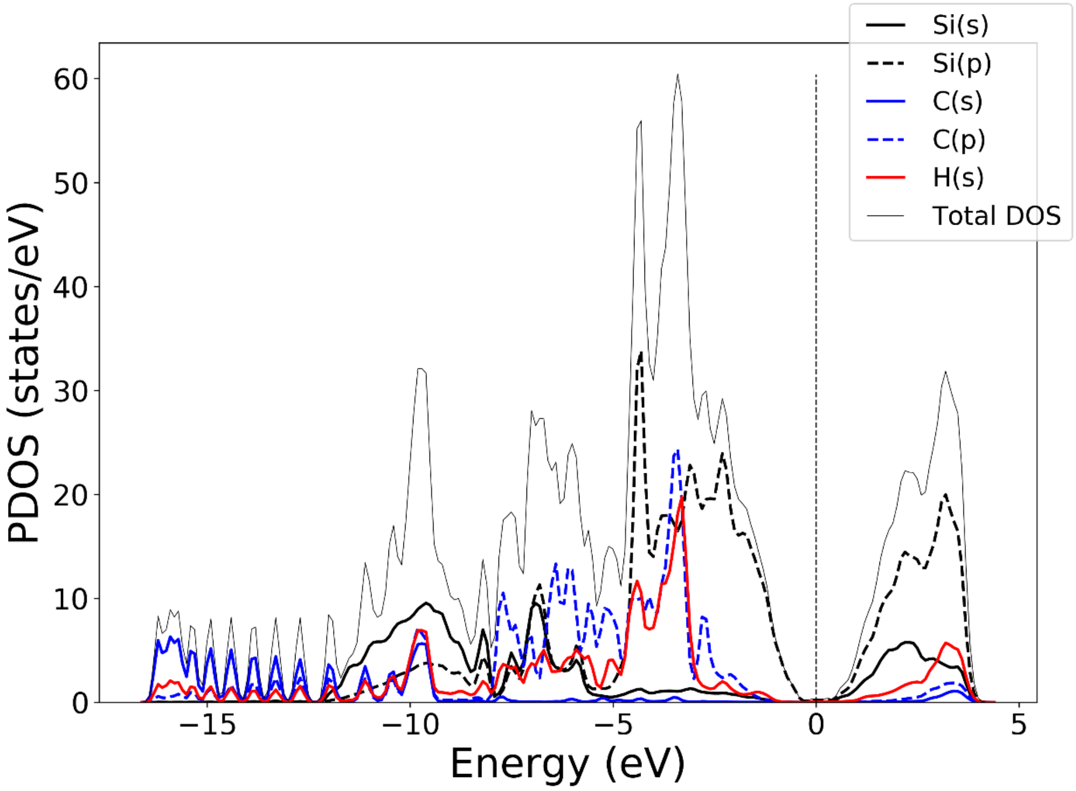

Figure S28. Projected density of states (PDOS) of C_{18} 1-alkynyl moiety adsorbed on H−Si(111) slab.

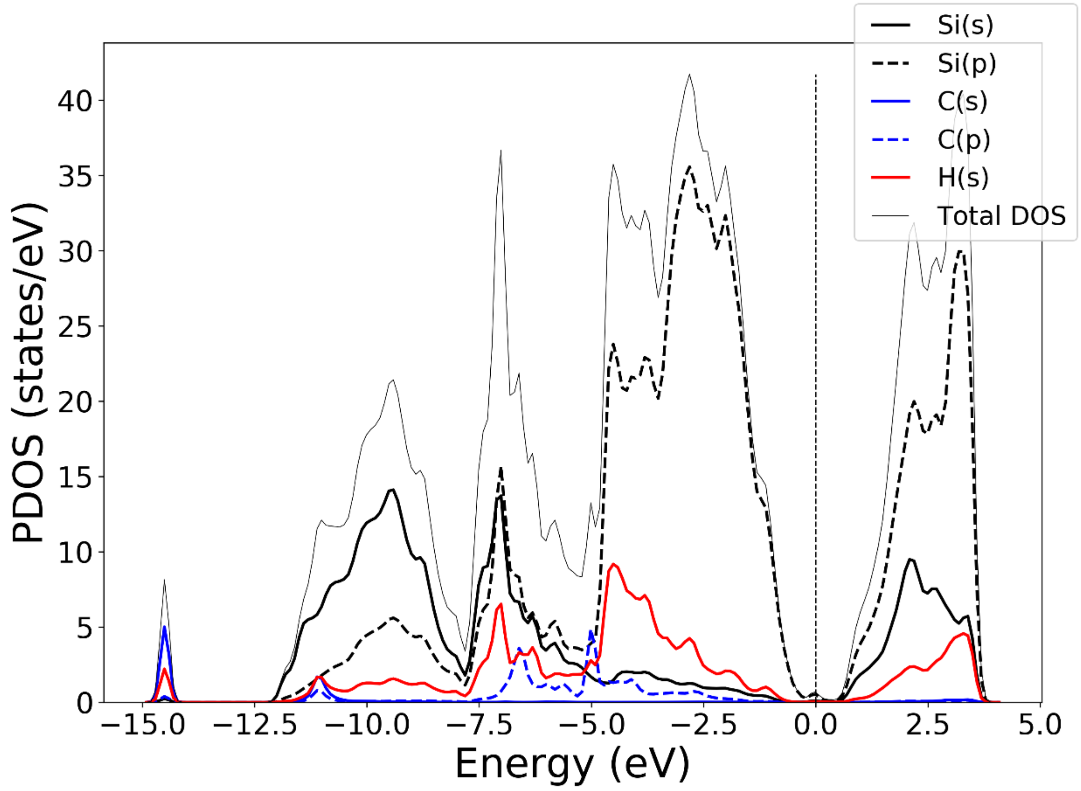

Figure S29. Projected density of states (PDOS) of C_2 alkyl moiety adsorbed on H−Si(110) slab.

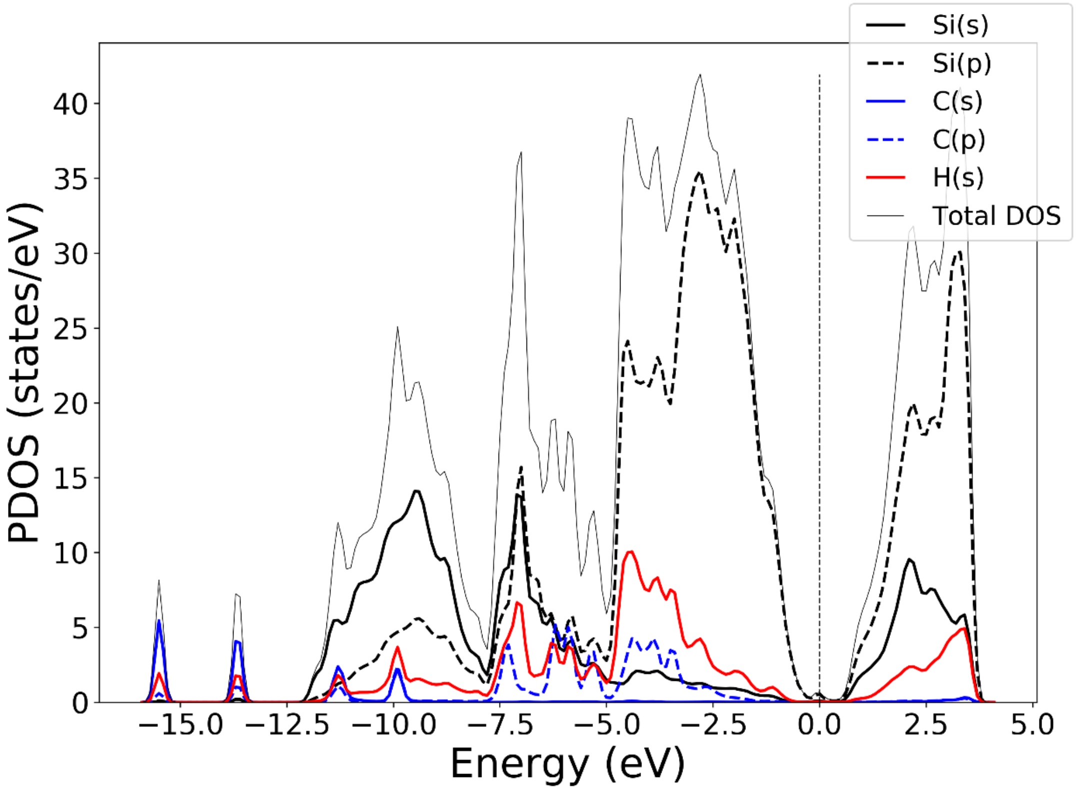

Figure S30. Projected density of states (PDOS) of C_4 alkyl moiety adsorbed on H−Si(110) slab.

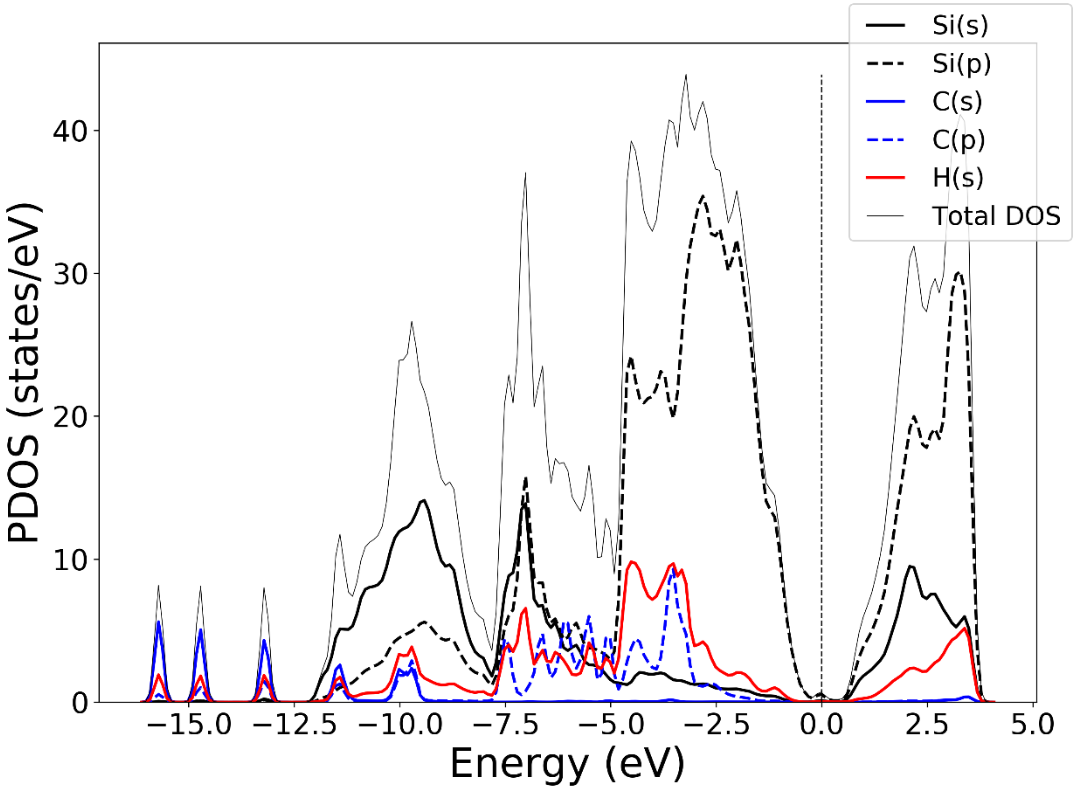

Figure S31. Projected density of states (PDOS) of C_6 alkyl moiety adsorbed on H−Si(110) slab.

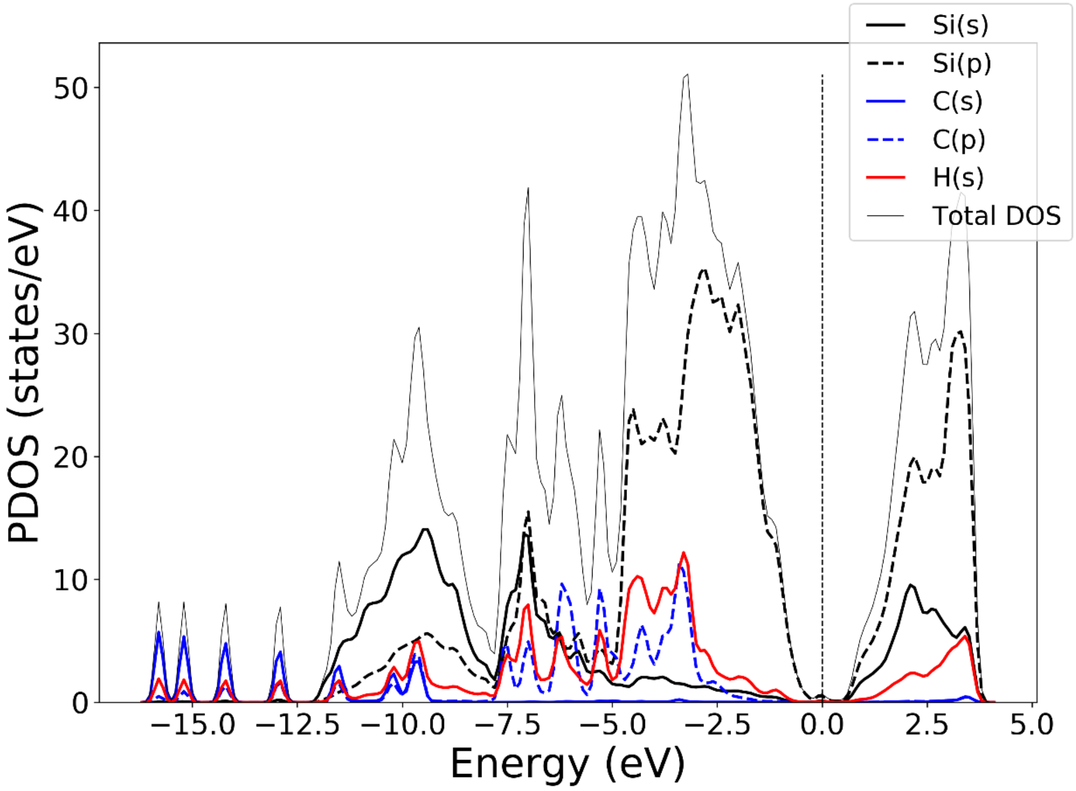

Figure S32. Projected density of states (PDOS) of C_8 alkyl moiety adsorbed on H−Si(110) slab.

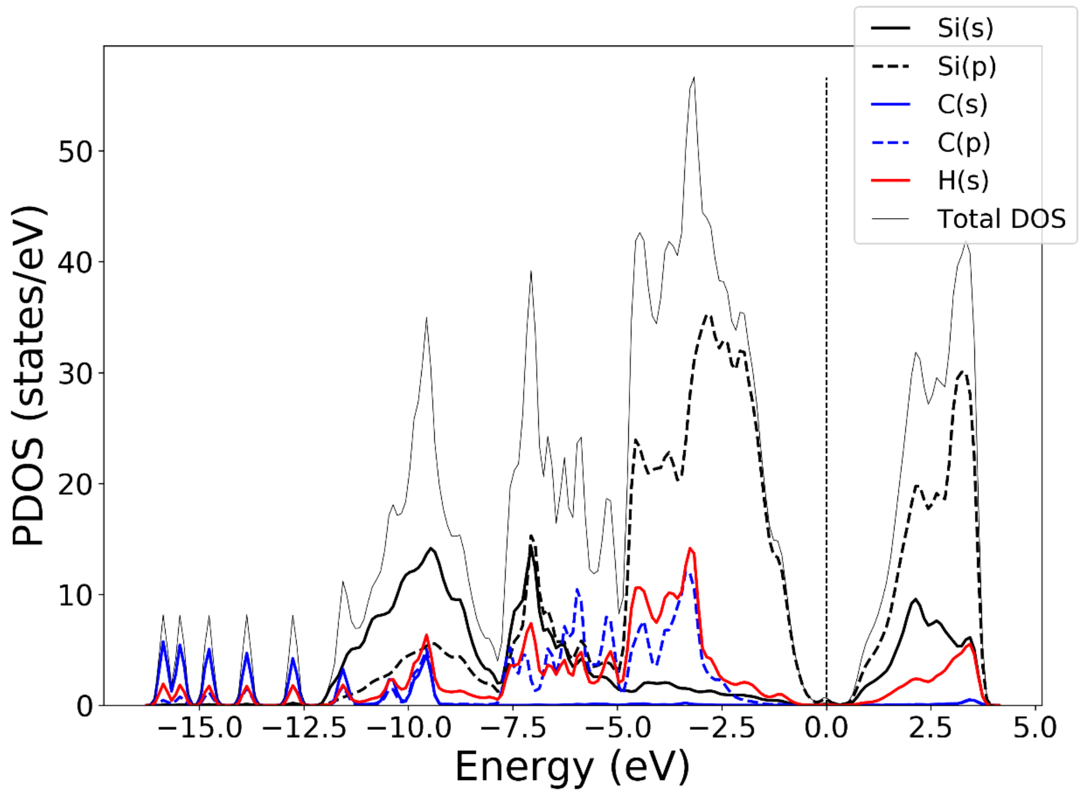

Figure S33. Projected density of states (PDOS) of C_{10} alkyl moiety adsorbed on H−Si(110) slab.

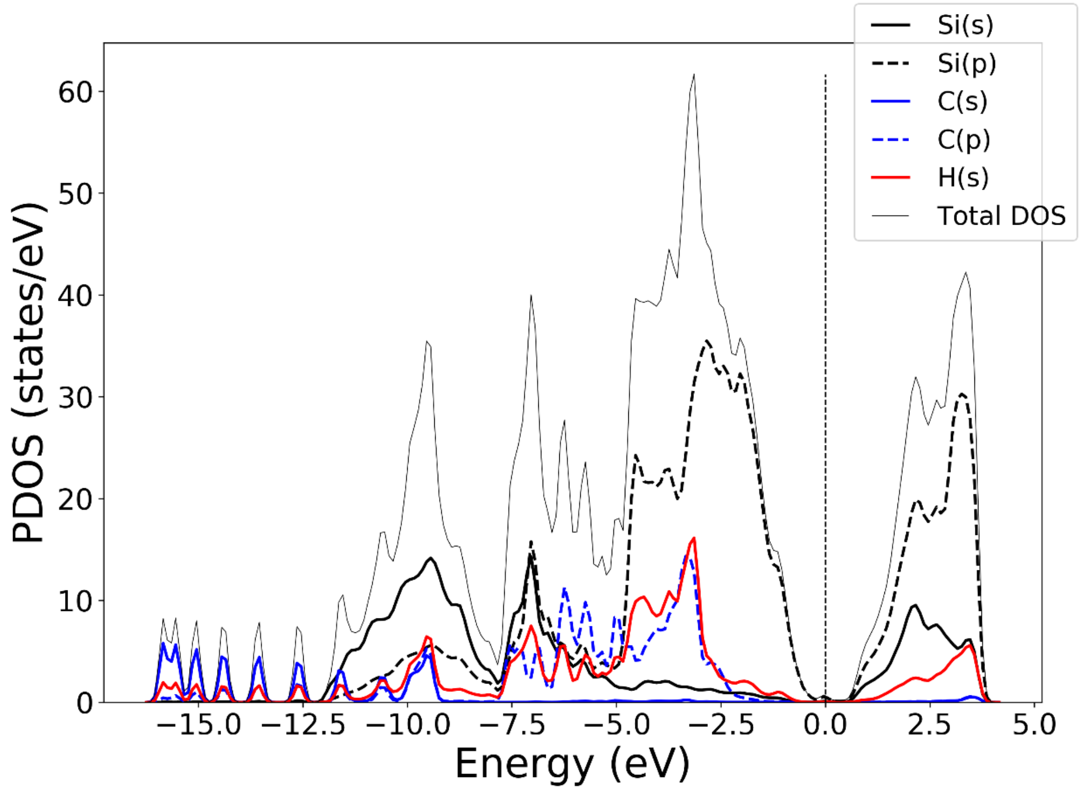

Figure S34. Projected density of states (PDOS) of C_{12} alkyl moiety adsorbed on H−Si(110) slab.

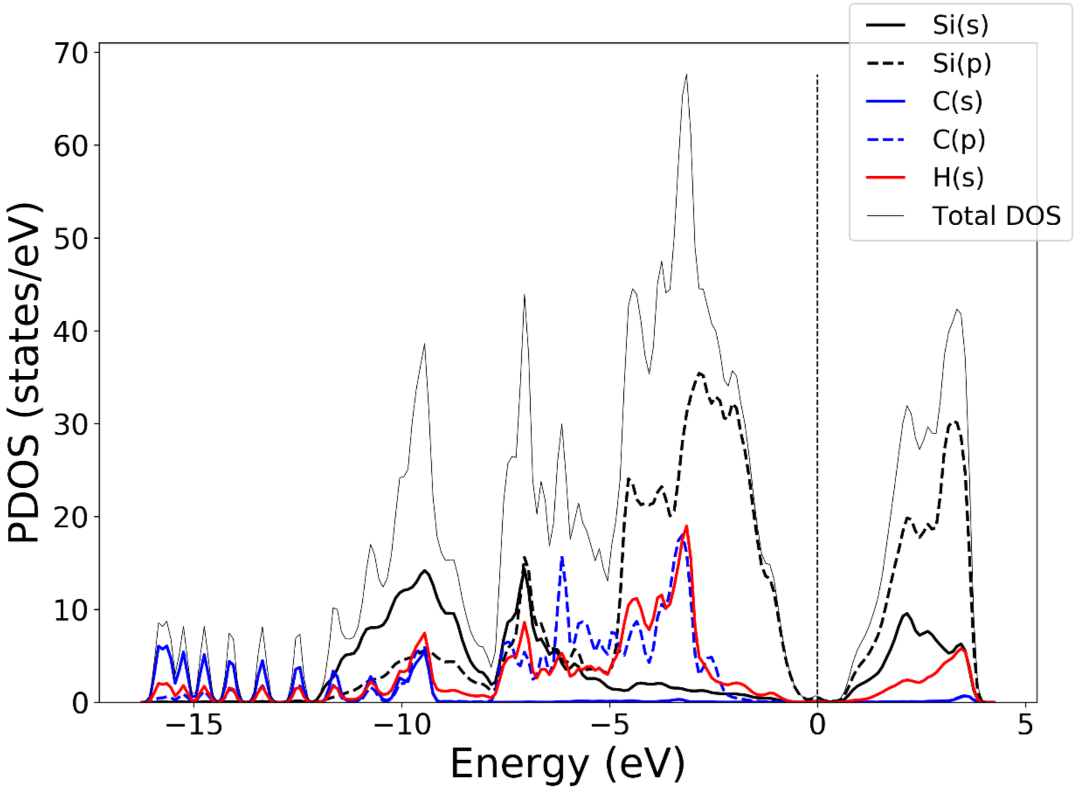

Figure S35. Projected density of states (PDOS) of C_{14} alkyl moiety adsorbed on H−Si(110) slab.

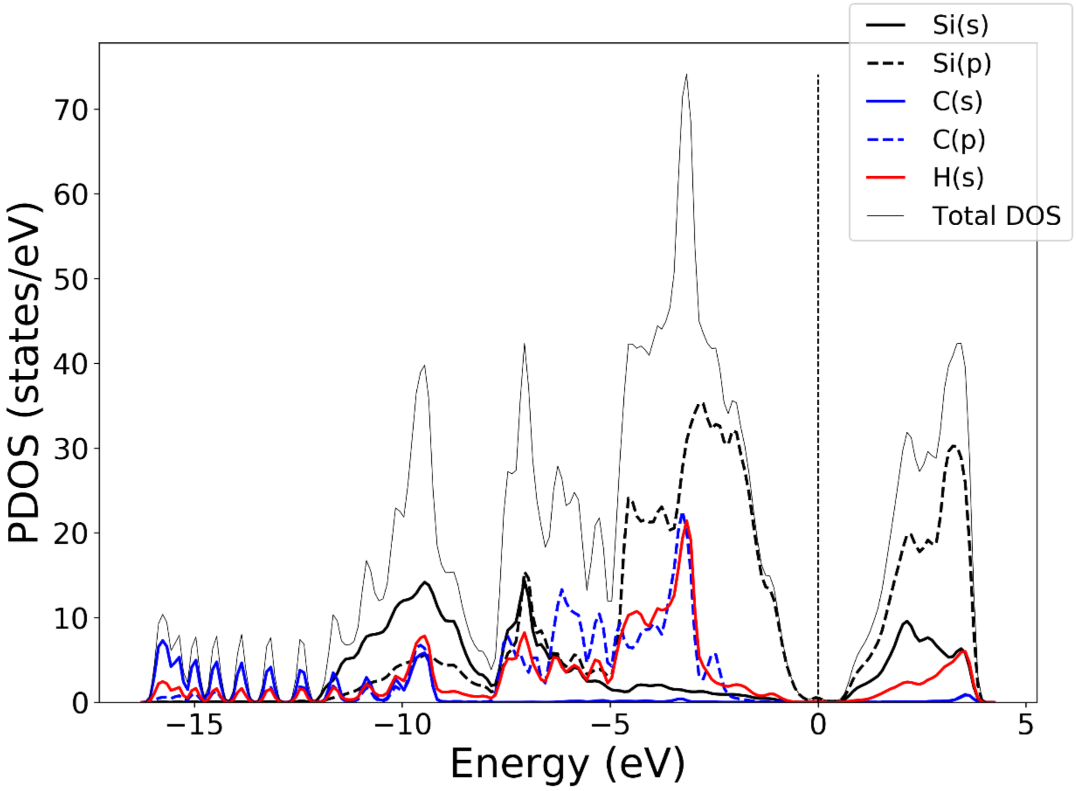

Figure S36. Projected density of states (PDOS) of C_{16} alkyl moiety adsorbed on H−Si(110) slab.

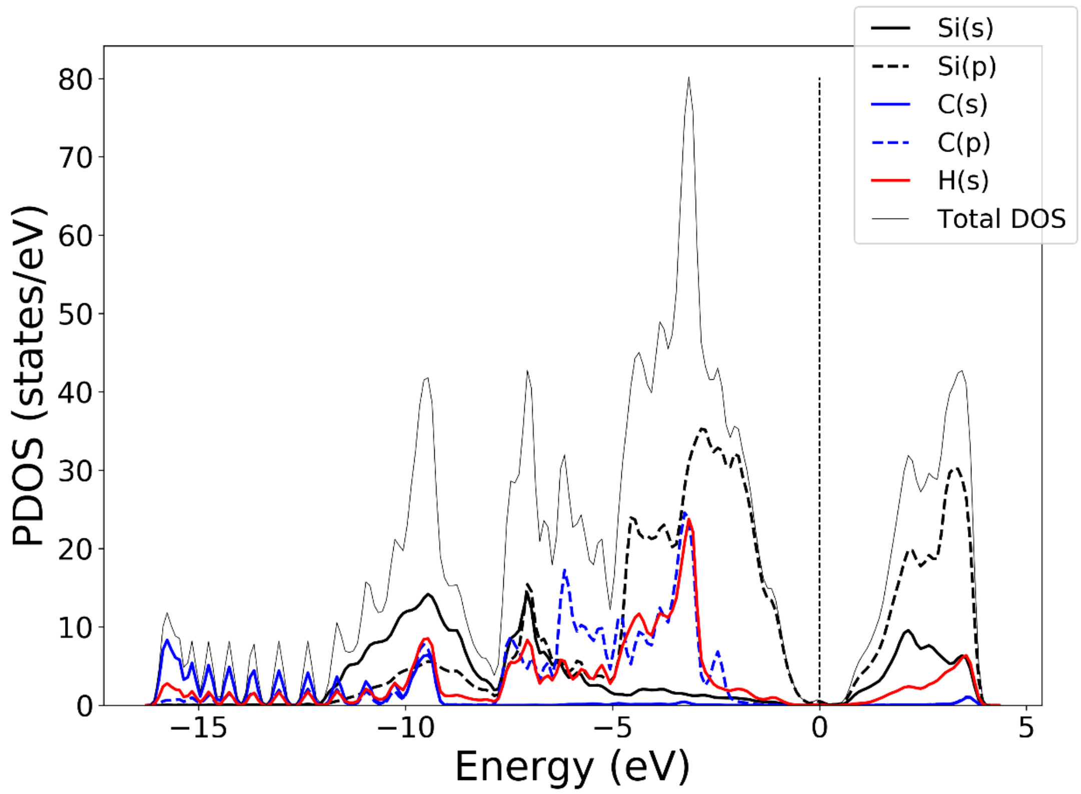

Figure S37. Projected density of states (PDOS) of C_{18} alkyl moiety adsorbed on H−Si(110) slab.

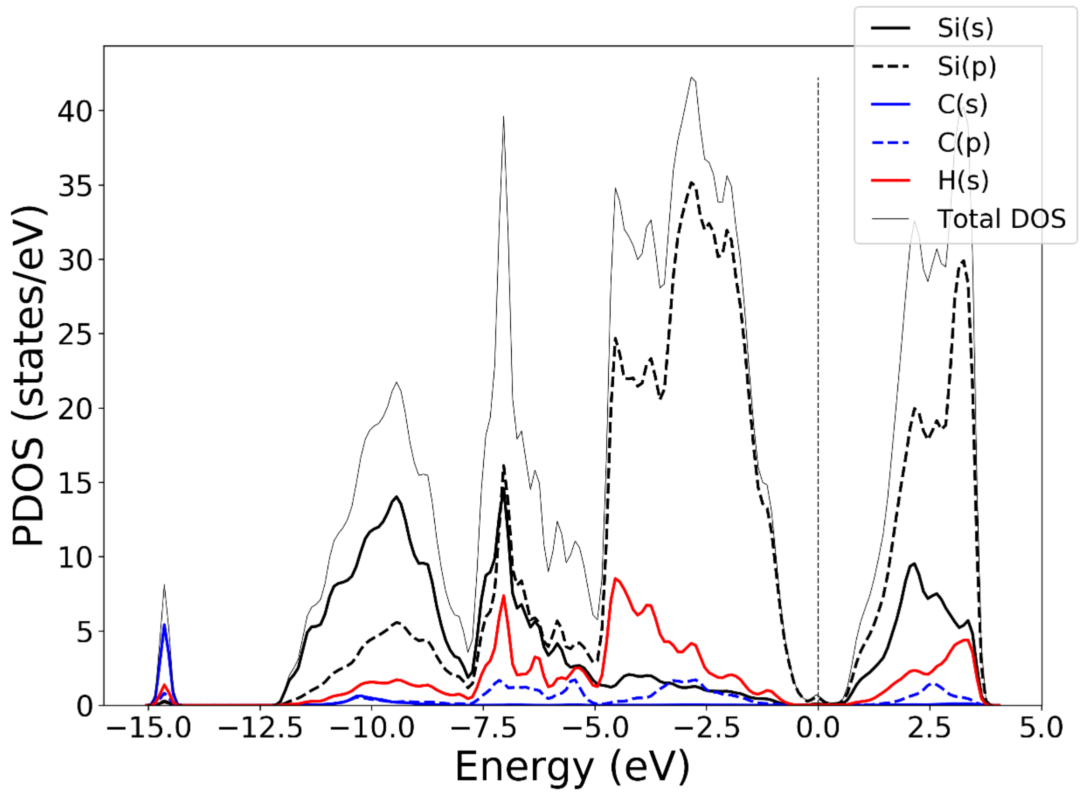

Figure S38. Projected density of states (PDOS) of C_2 alkenyl moiety adsorbed on H−Si(110) slab.

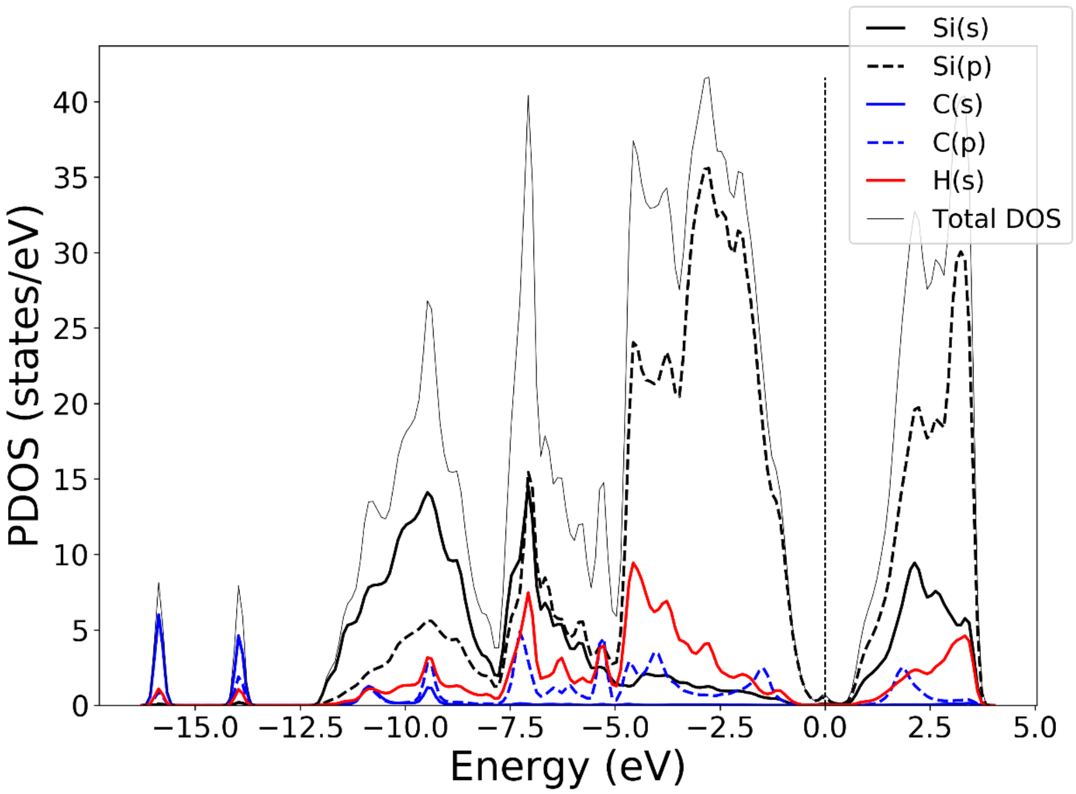

Figure S39. Projected density of states (PDOS) of C_4 alkenyl moiety adsorbed on H−Si(110) slab.

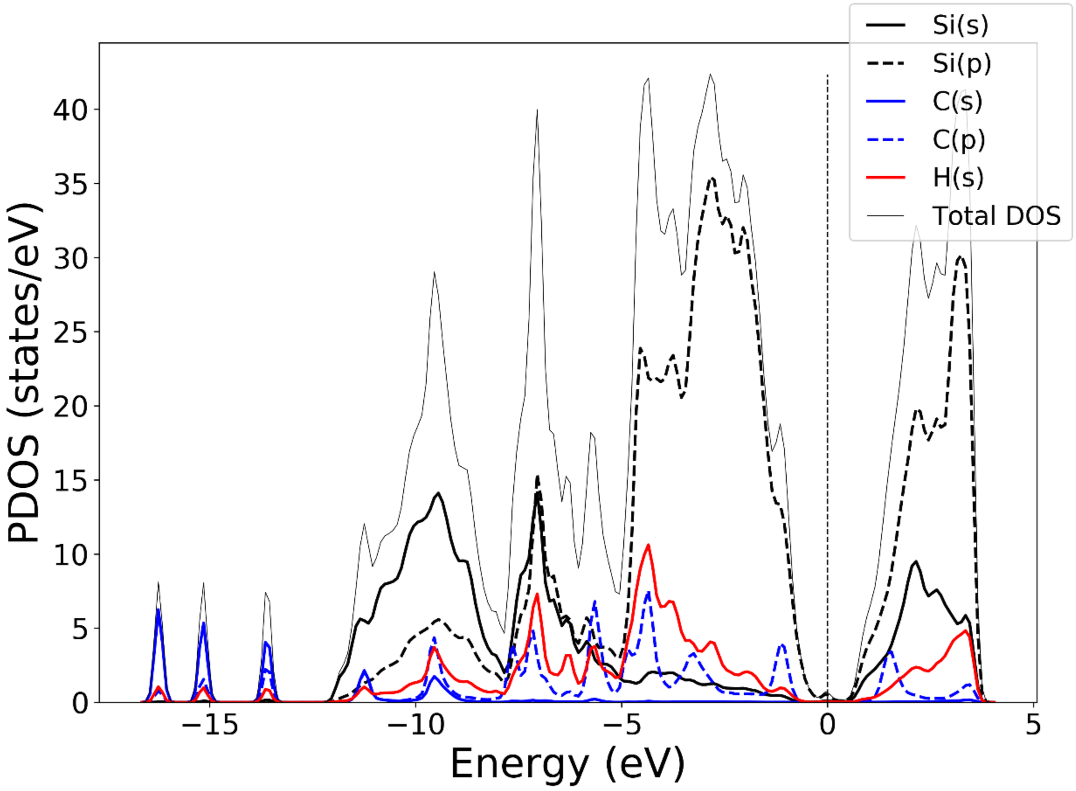

Figure S40. Projected density of states (PDOS) of C_6 alkenyl moiety adsorbed on H−Si(110) slab.

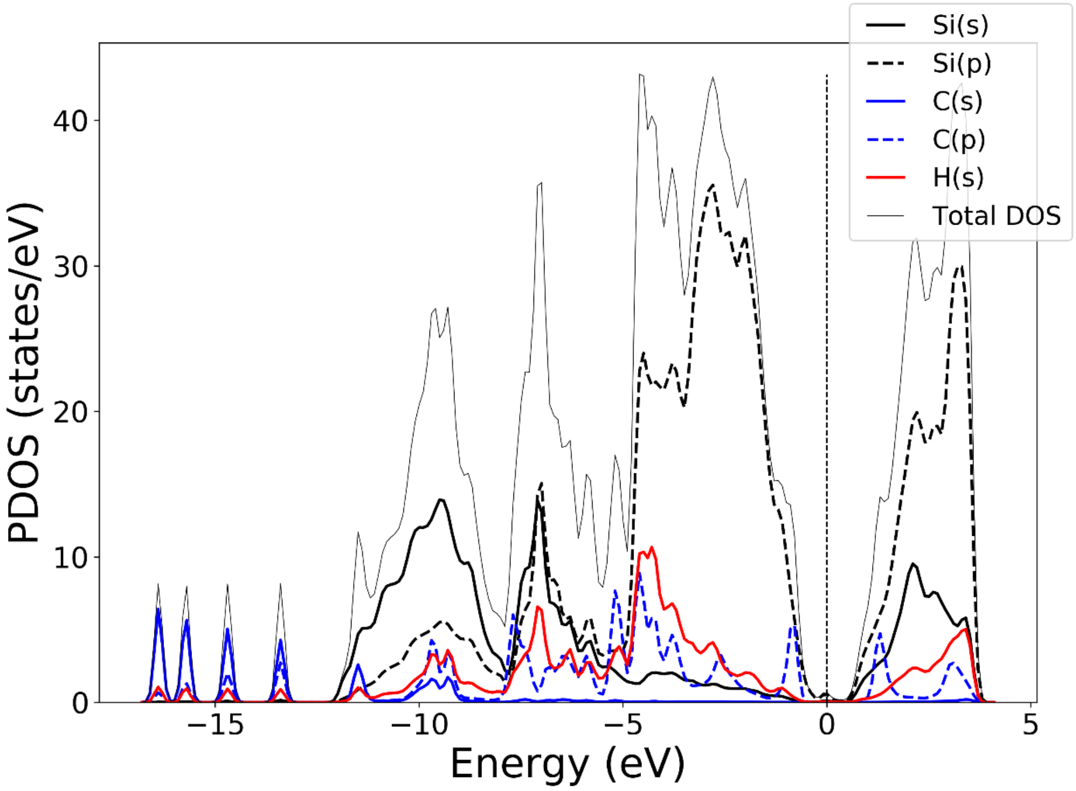

Figure S41. Projected density of states (PDOS) of C_8 alkenyl moiety adsorbed on H−Si(110) slab.

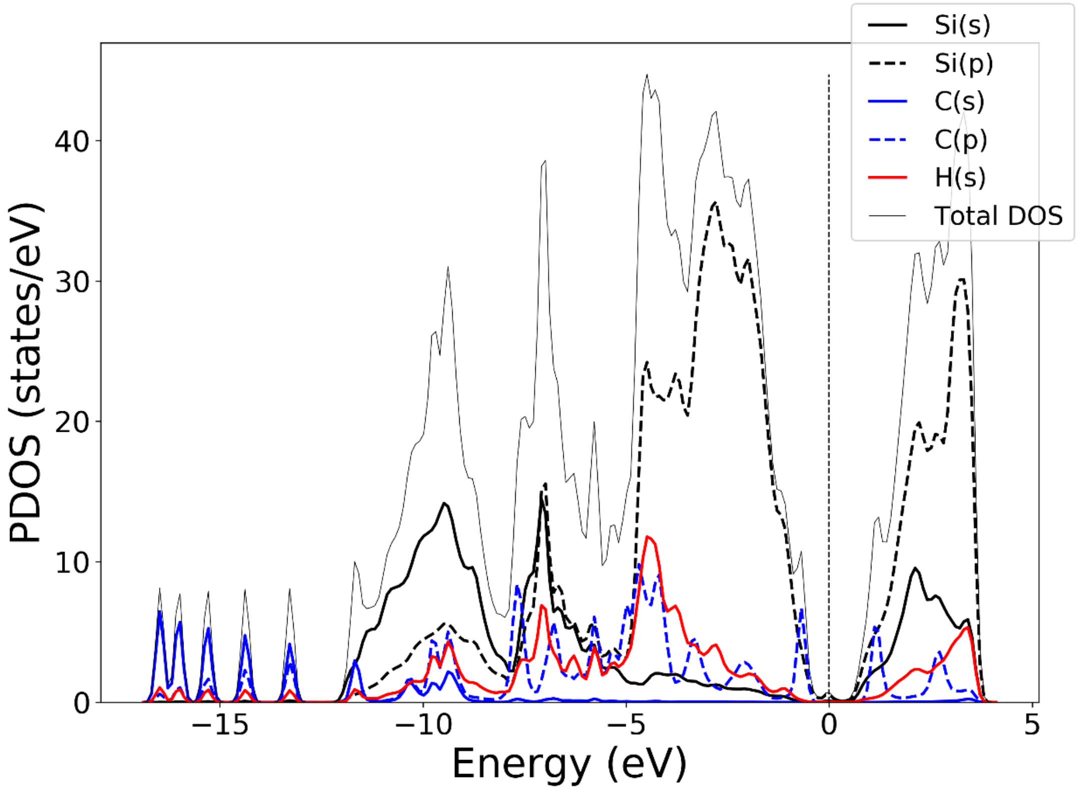

Figure S42. Projected density of states (PDOS) of C_{10} alkenyl moiety adsorbed on H−Si(110) slab.

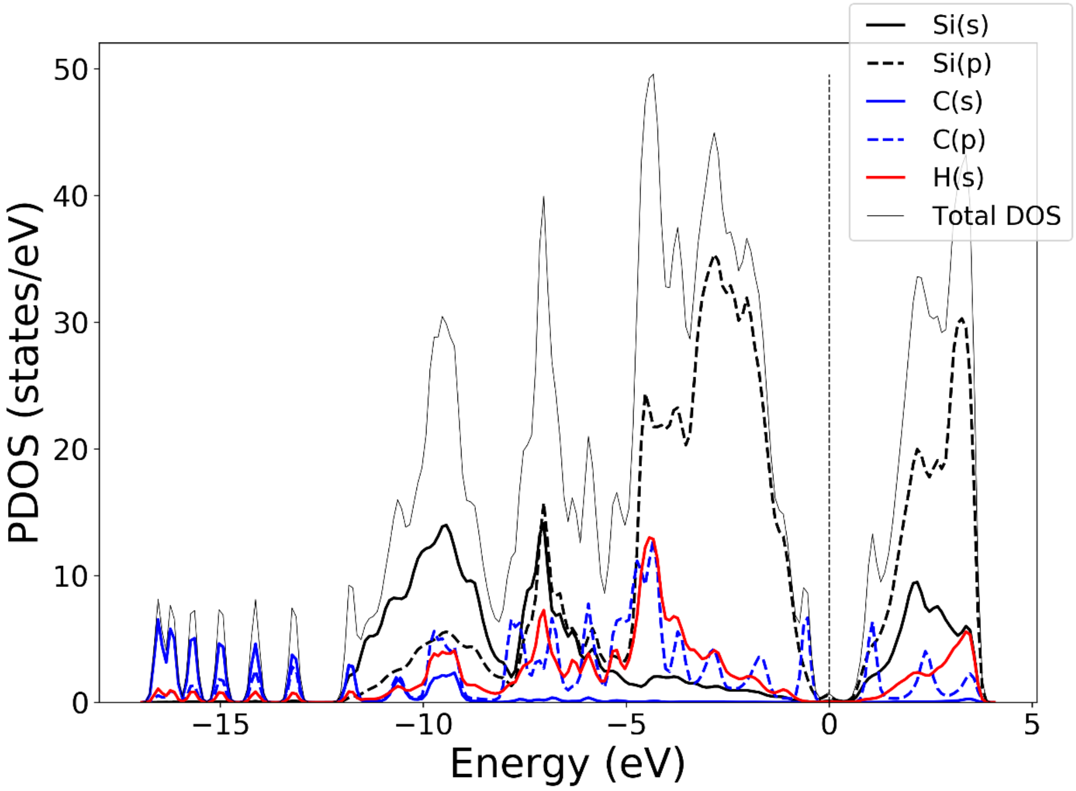

Figure S43. Projected density of states (PDOS) of C_{12} alkenyl moiety adsorbed on H−Si(110) slab.

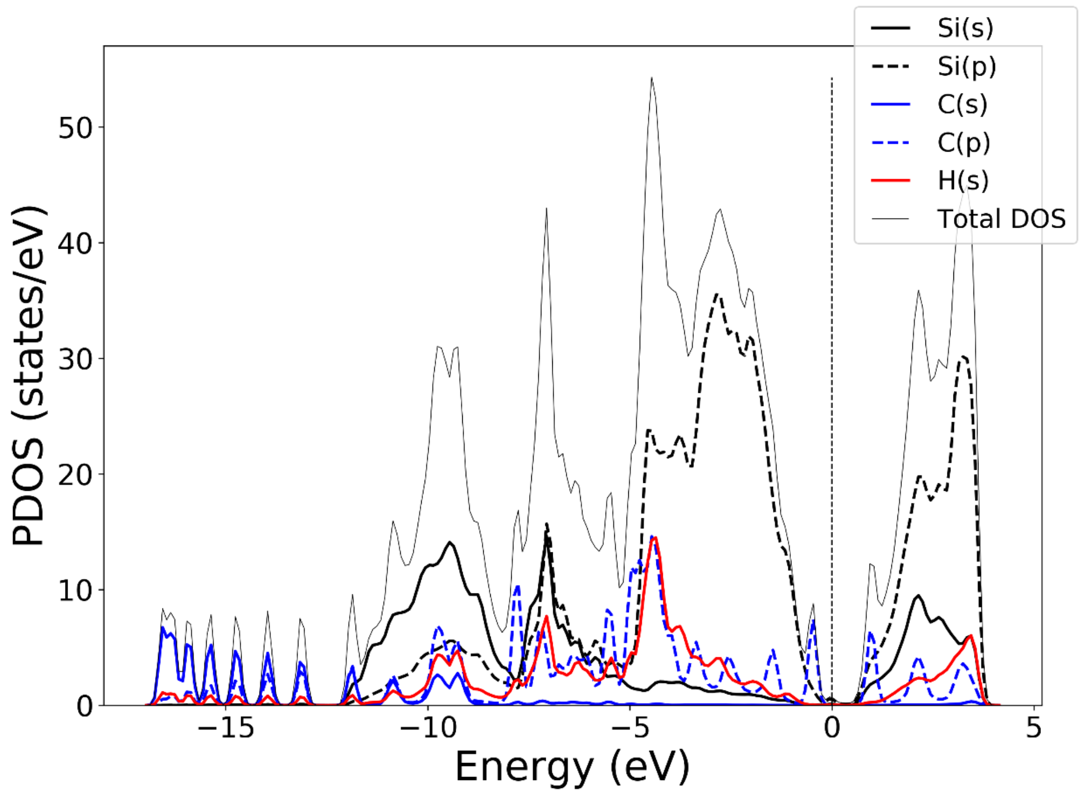

Figure S44. Projected density of states (PDOS) of C_{14} alkenyl moiety adsorbed on H−Si(110) slab.

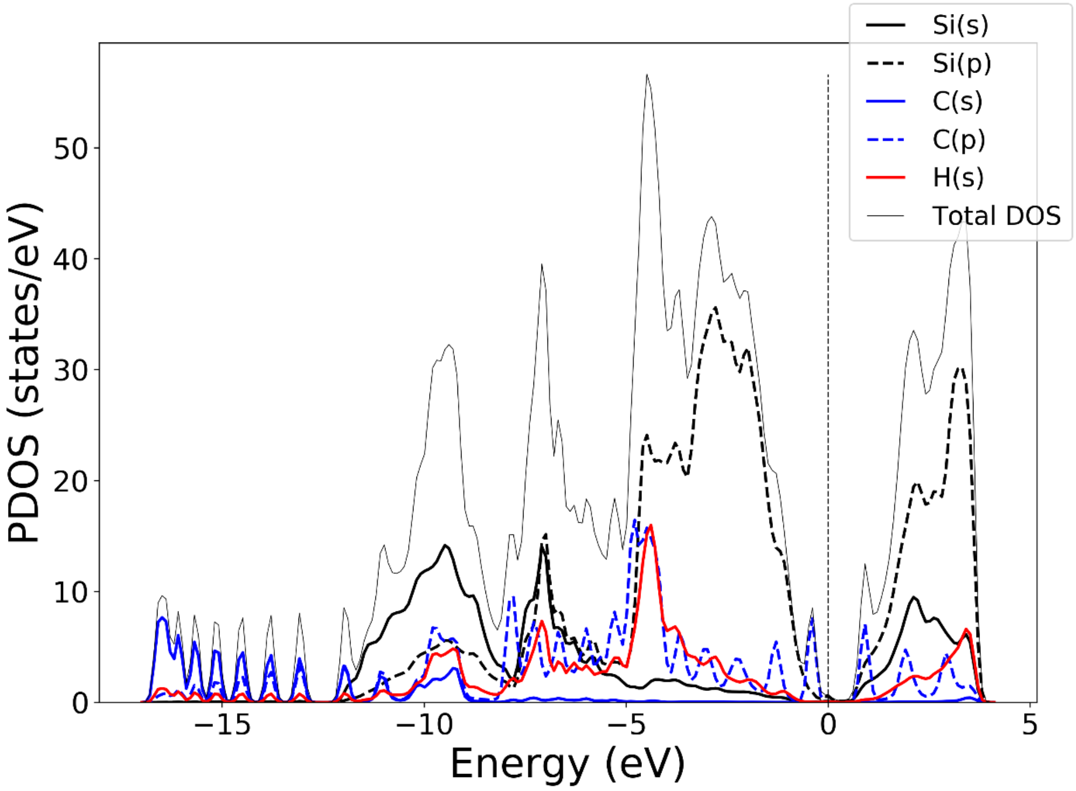

Figure S45. Projected density of states (PDOS) of C_{16} alkenyl moiety adsorbed on H−Si(110) slab.

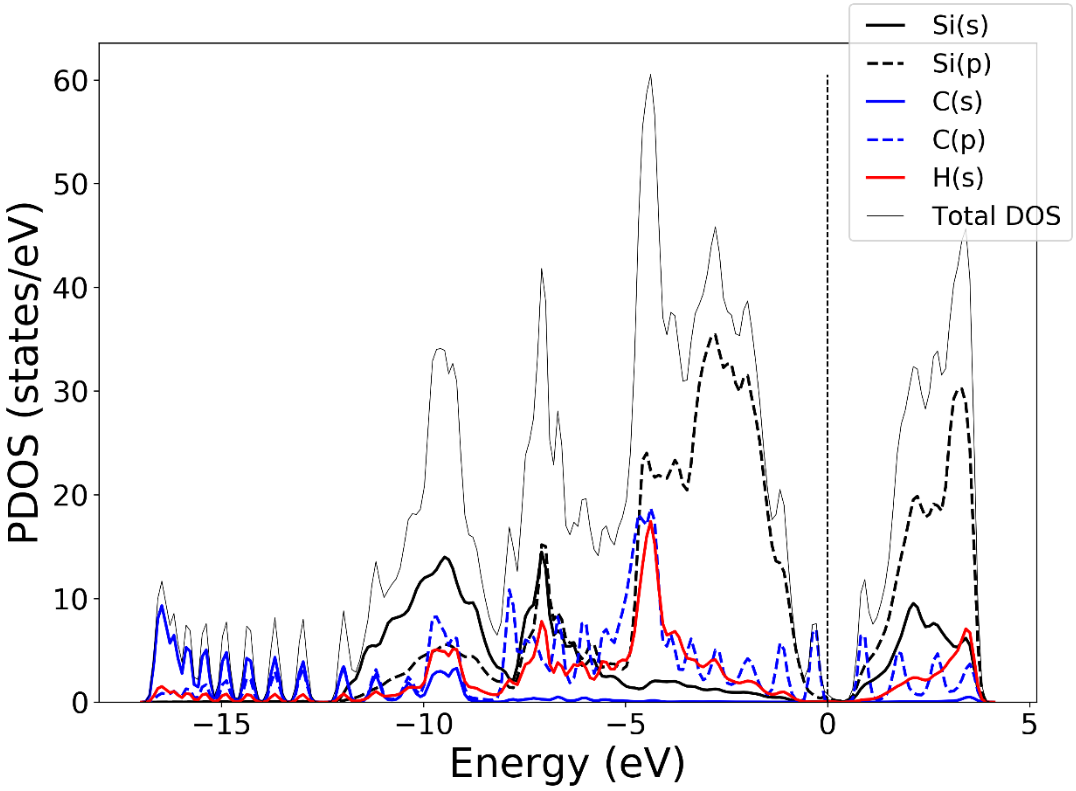

Figure S46. Projected density of states (PDOS) of C_{18} alkenyl moiety adsorbed on H−Si(110) slab.

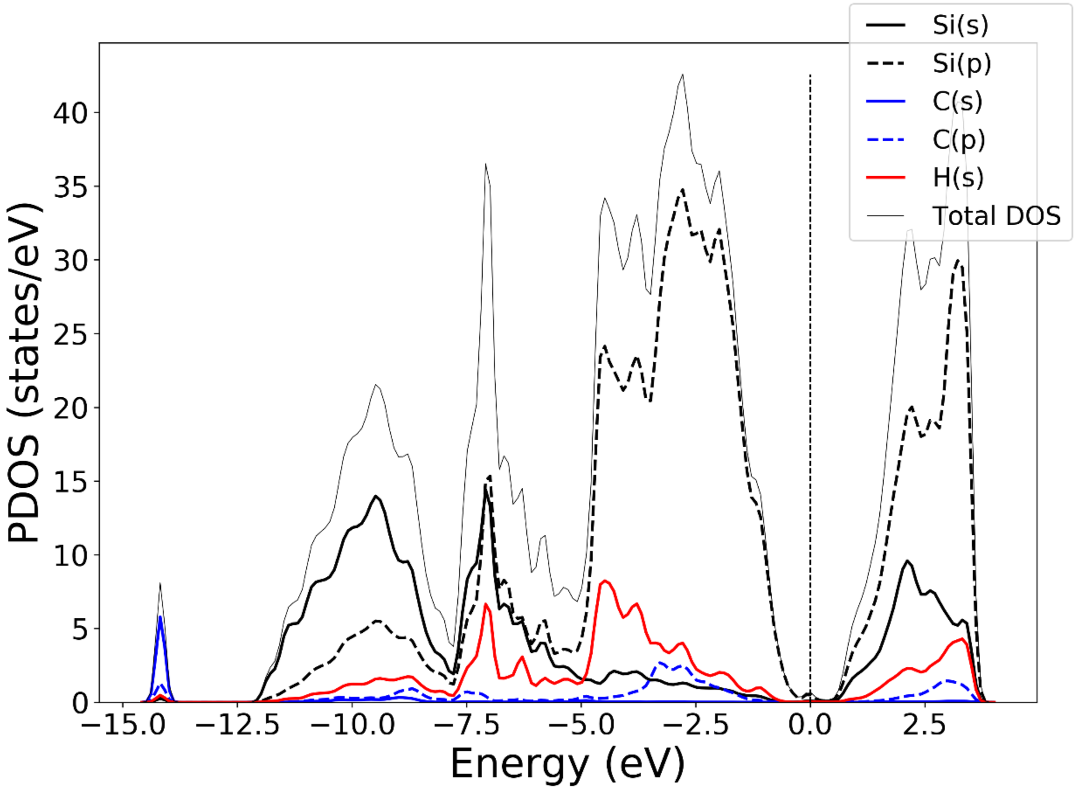

Figure S47. Projected density of states (PDOS) of C_2 1-alkynyl moiety adsorbed on H−Si(110) slab.

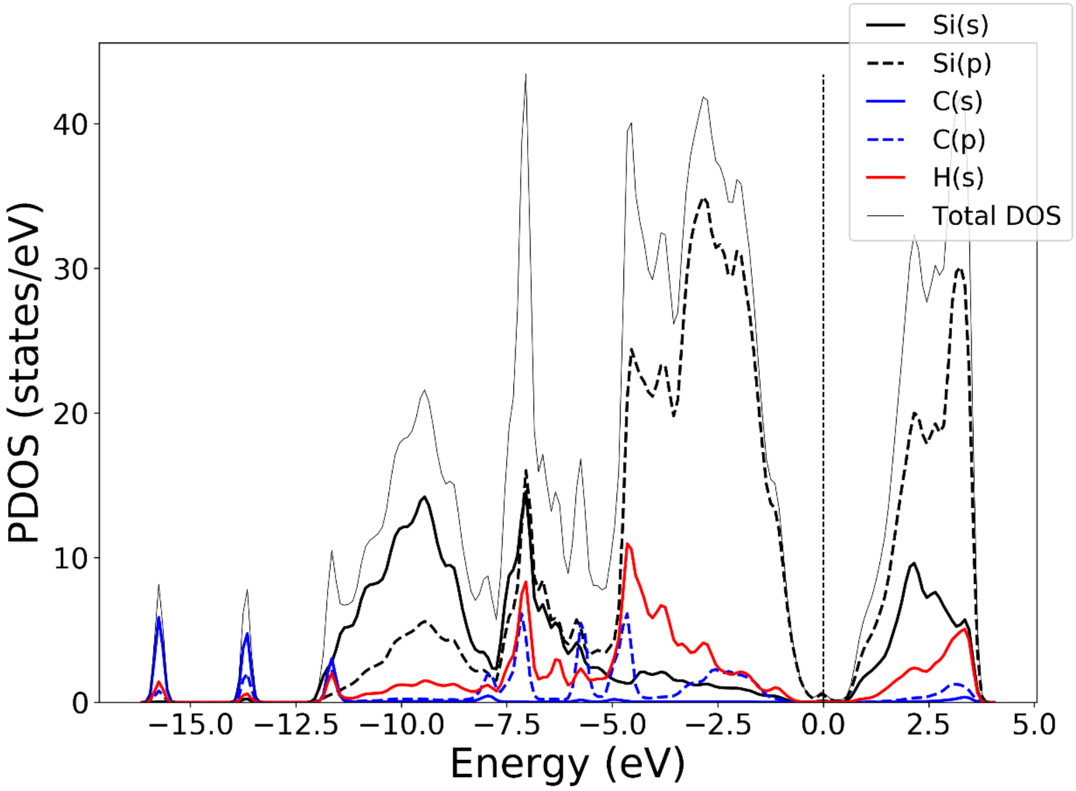

Figure S48. Projected density of states (PDOS) of C_4 1-alkynyl moiety adsorbed on H−Si(110) slab.

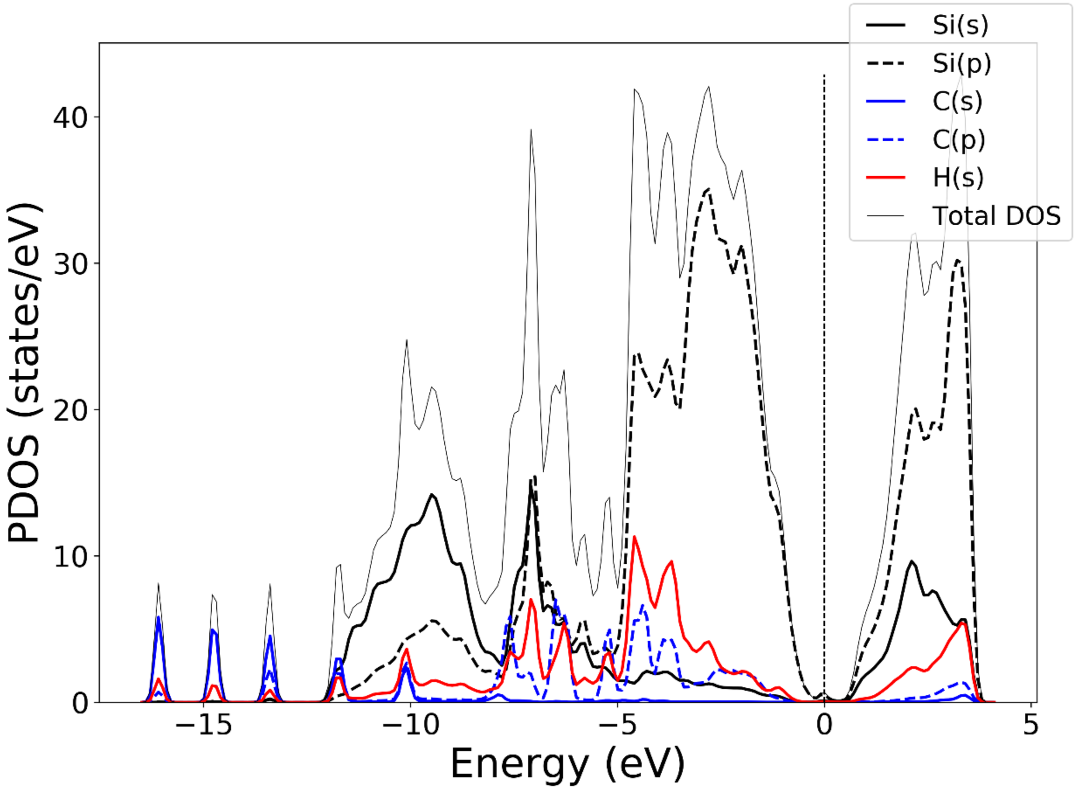

Figure S49. Projected density of states (PDOS) of C_6 1-alkynyl moiety adsorbed on H−Si(110) slab.

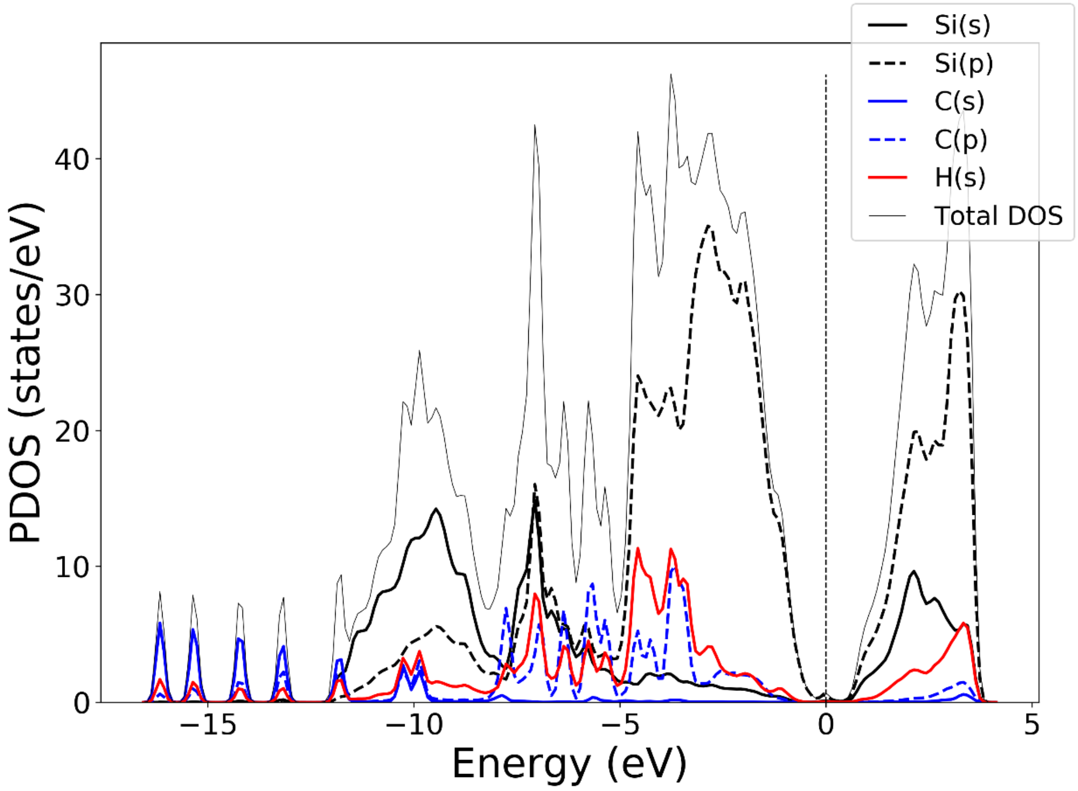

Figure S50. Projected density of states (PDOS) of C_8 1-alkynyl moiety adsorbed on H−Si(110) slab.

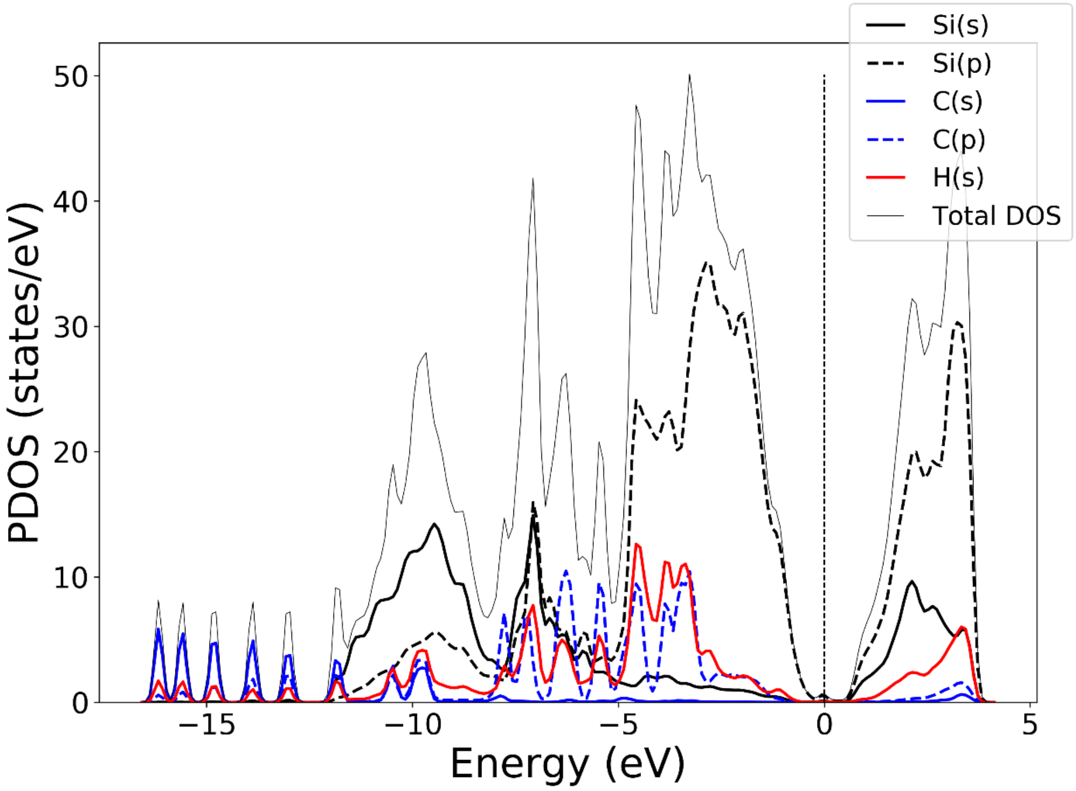

Figure S51. Projected density of states (PDOS) of C_{10} 1-alkynyl moiety adsorbed on H−Si(110) slab.

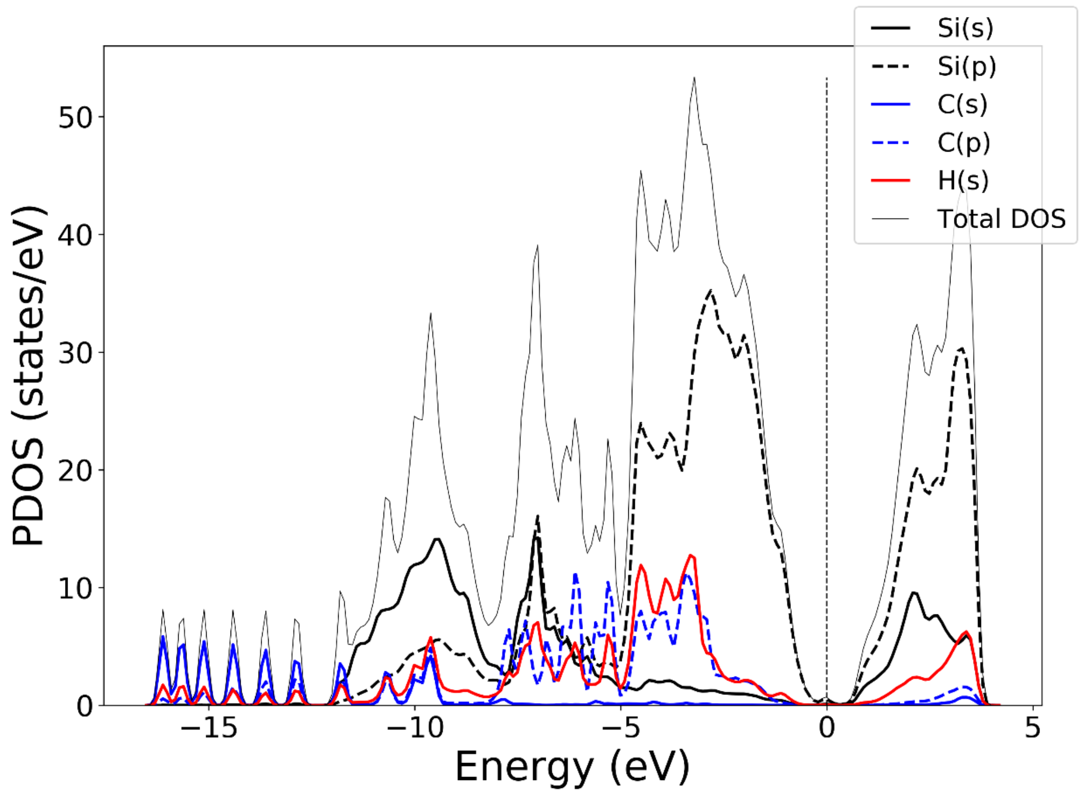

Figure S52. Projected density of states (PDOS) of C_{12} 1-alkynyl moiety adsorbed on H−Si(110) slab.

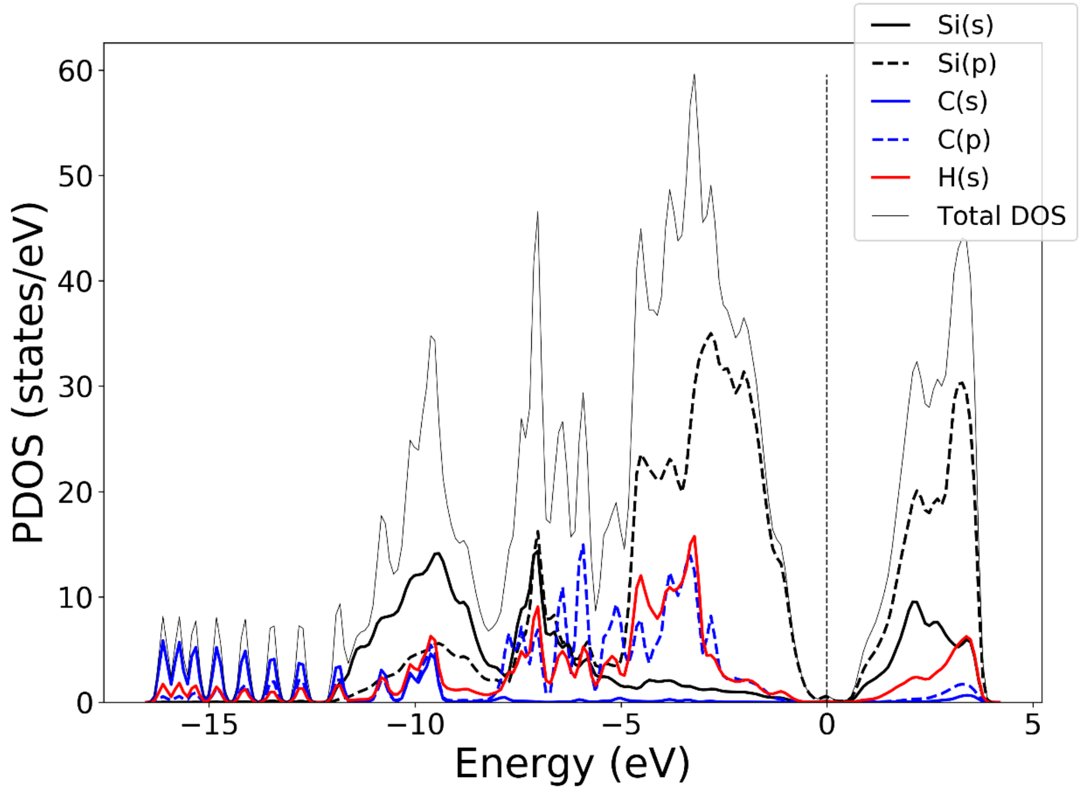

Figure S53. Projected density of states (PDOS) of C_{14} 1-alkynyl moiety adsorbed on H−Si(110) slab.

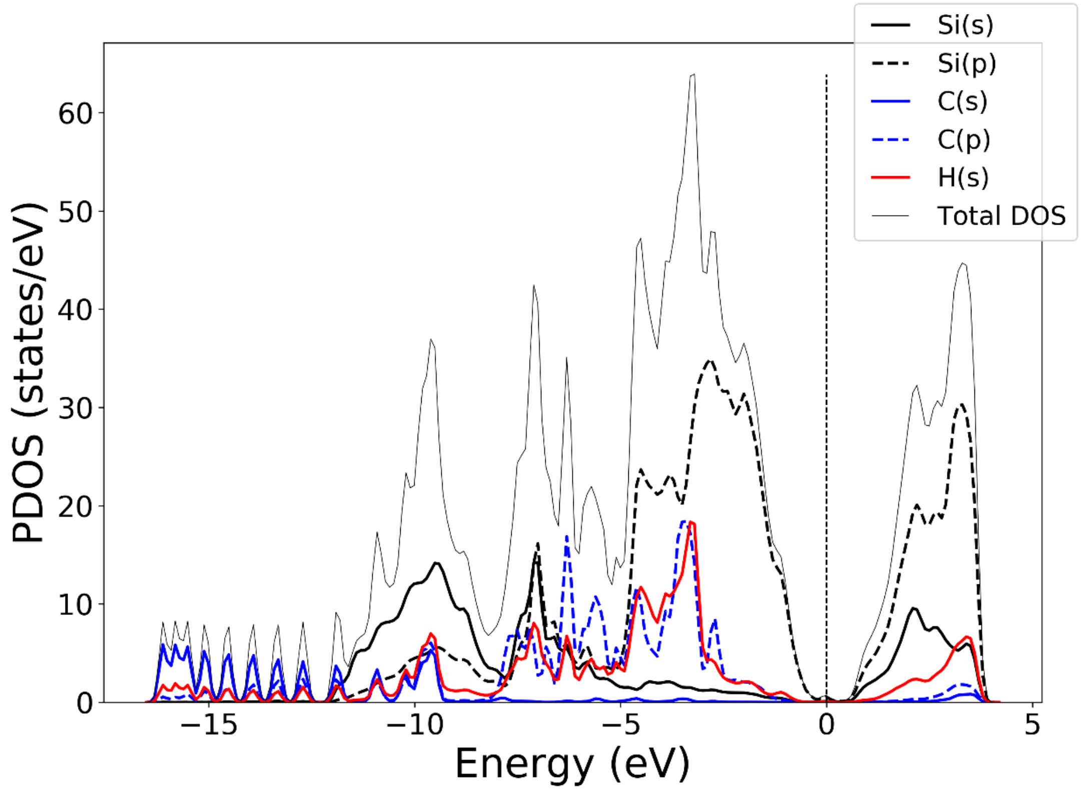

Figure S54. Projected density of states (PDOS) of C_{16} 1-alkynyl moiety adsorbed on H−Si(110) slab.

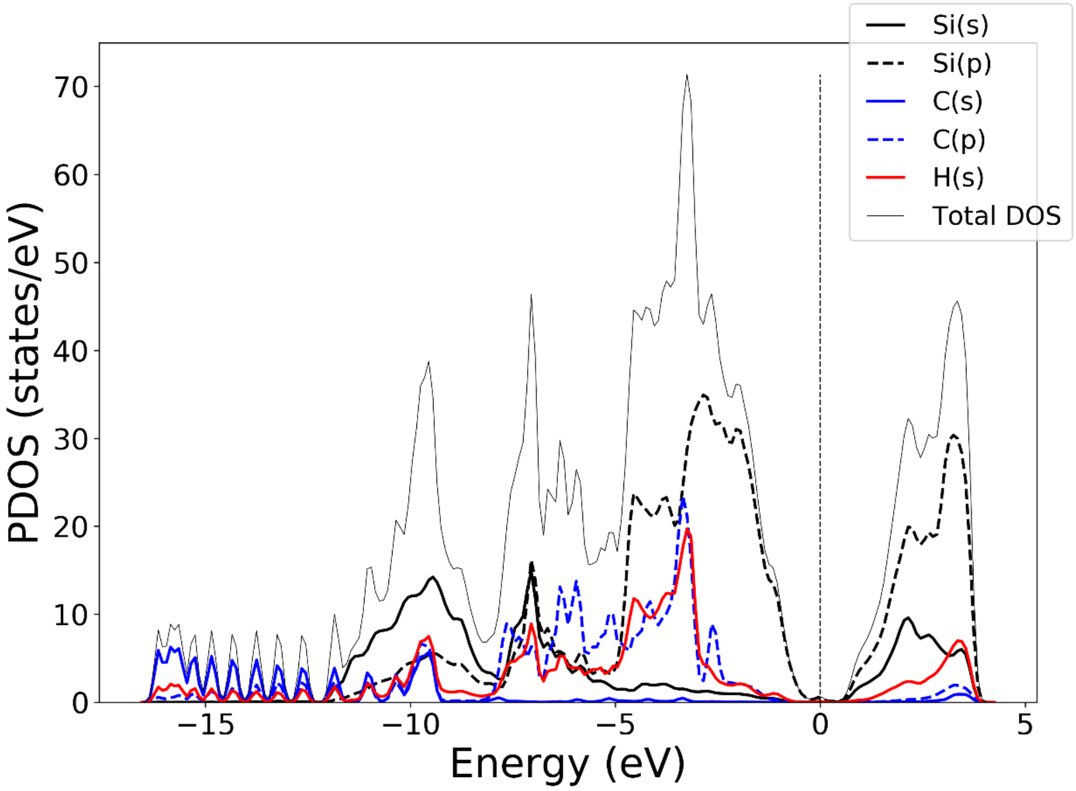

Figure S55. Projected density of states (PDOS) of C_{18} 1-alkynyl moiety adsorbed on H−Si(110) slab.

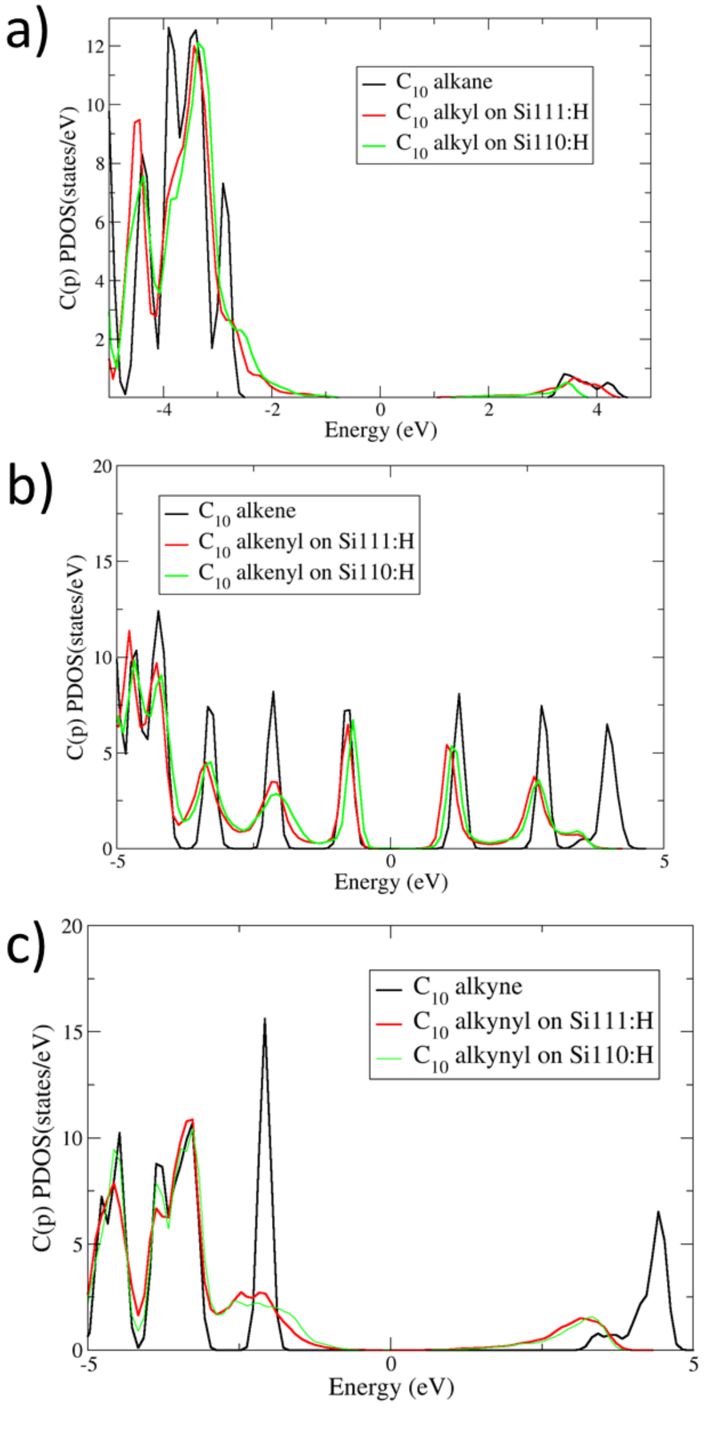

Figure S56. Comparison of projected density of states (PDOS) of a) alkyl, b) alkenyl and c) 1-alkynyl C_{10} moieties adsorbed on H−Si(111) and H−Si(110) slabs. HOMO and LUMO energies have been determined by comparison with the PDOS of the corresponding isolated molecules.

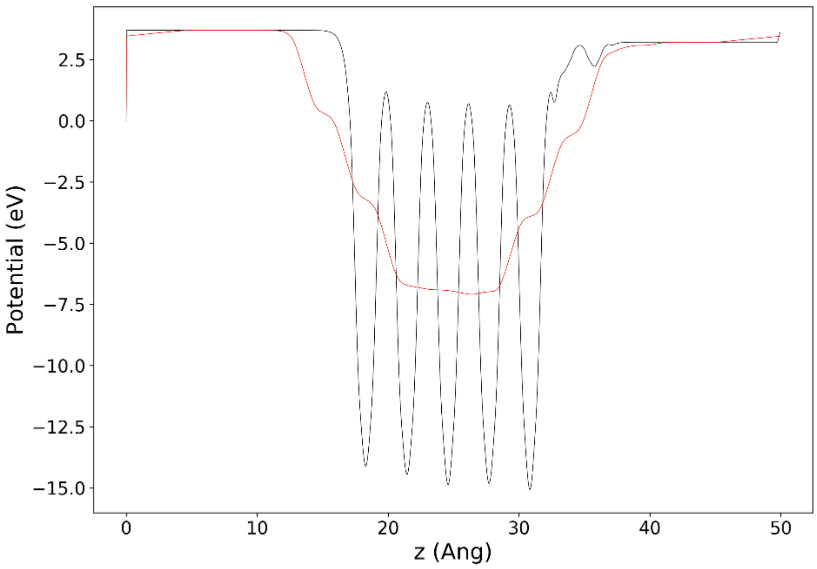

Figure S57. Plane-averaged electrostatic energy calculated on 1 (black) and 5 (red) Å step of C_2 alkyl moiety adsorbed on H−Si(111) slab. We estimated the vacuum level of each adsorption configuration as the constant plane-averaged electrostatic energy in the vacuum gap far away from the top atomic layer.

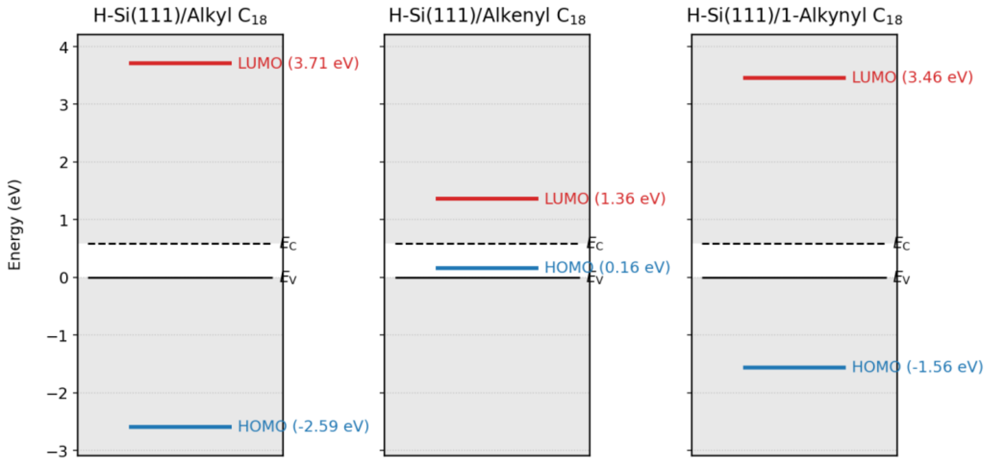

Figure S58. Energy levels schematics of alkyl, alkenyl and 1-alkynyl C_{18} moieties chemisorbed on H-Si(111) surface.

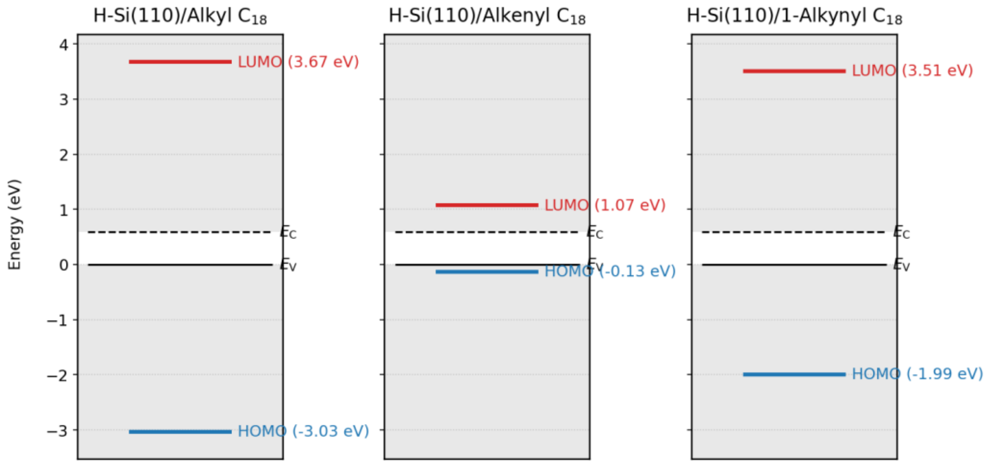

Figure S59. Energy levels schematics of alkyl, alkenyl and 1-alkynyl C_{18} moieties chemisorbed on H-Si(110) surface.

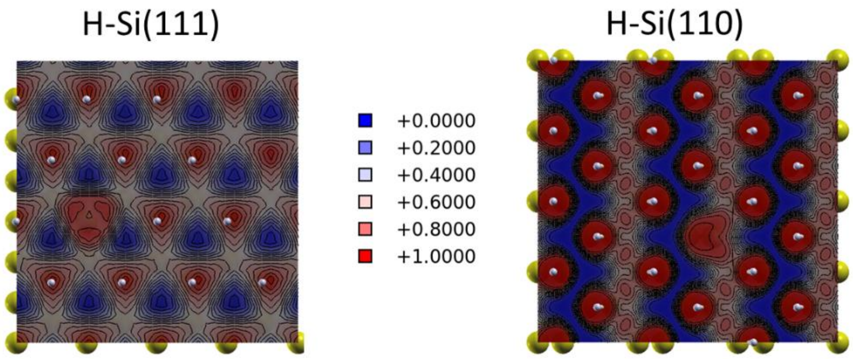

Figure S60. Electron localization function (ELF) of H−Si(111) (left) and H−Si(110) (right) slabs upon removal of one hydrogen atom (dangling bond).

Table S1. ΔP_z values as a function of supercell size (i.e., surface coverage) for the Si(110) orientation, considering the three types of C4 molecules: alkyl, alkenyl, and 1-alkynyl. A ~100% variation in the supercell area results in a maximum change in the dipole of about 12%.

	C_4 alkyl on Si(110)		C_4 alkenyl on Si(110)		C_4 1-alkynyl on Si(110)	
Surface Coverage (nm^{-2})	0.79	0.39	0.79	0.39	0.79	0.39
Surface Area ($Å^2$)	127	254	127	254	127	254
ΔP_z (D)	0.642	0.695	0.712	0.762	1.213	1.381
Change (%)	7.5		6.5		12.2	
